# Supplementary material for: Epigenetic regulation of neural stem cell aging in the mouse hippocampus by Setd8 downregulation
Source: EMBO J. 2025 Jun 3;44(13):3645–68. doi: 10.1038/s44318-025-00455-8 (PMC12218407; doi:10.1038/s44318-025-00455-8)
Supplement: Supplementary file 1 — Appendix [file 44318_2025_455_MOESM1_ESM.pdf]

Appendix

Title:

Epigenetic Regulation of Neural Stem Cell Aging in the Mouse Hippocampus by Setd8 Downregulation

Running Title

Setd8 Downregulation Drives NSC Aging

Shuzo Matsubara, Kanae Matsuda-Ito, Haruka Sekiryu, Hiroyoshi Doi, Takumi Nakagawa, Naoya Murao, Hisanobu Oda, Kinichi Nakashima, Taito Matsuda

Contents

1 Appendix Figures

Appendix Figure S1 3
Appendix Figure S2 5
Appendix Figure S3 6
Appendix Figure S4 7
Appendix Figure S5 9
Appendix Figure S6 11
Appendix Figure S7 12
Appendix Figure S8 13
Appendix Figure S9 14

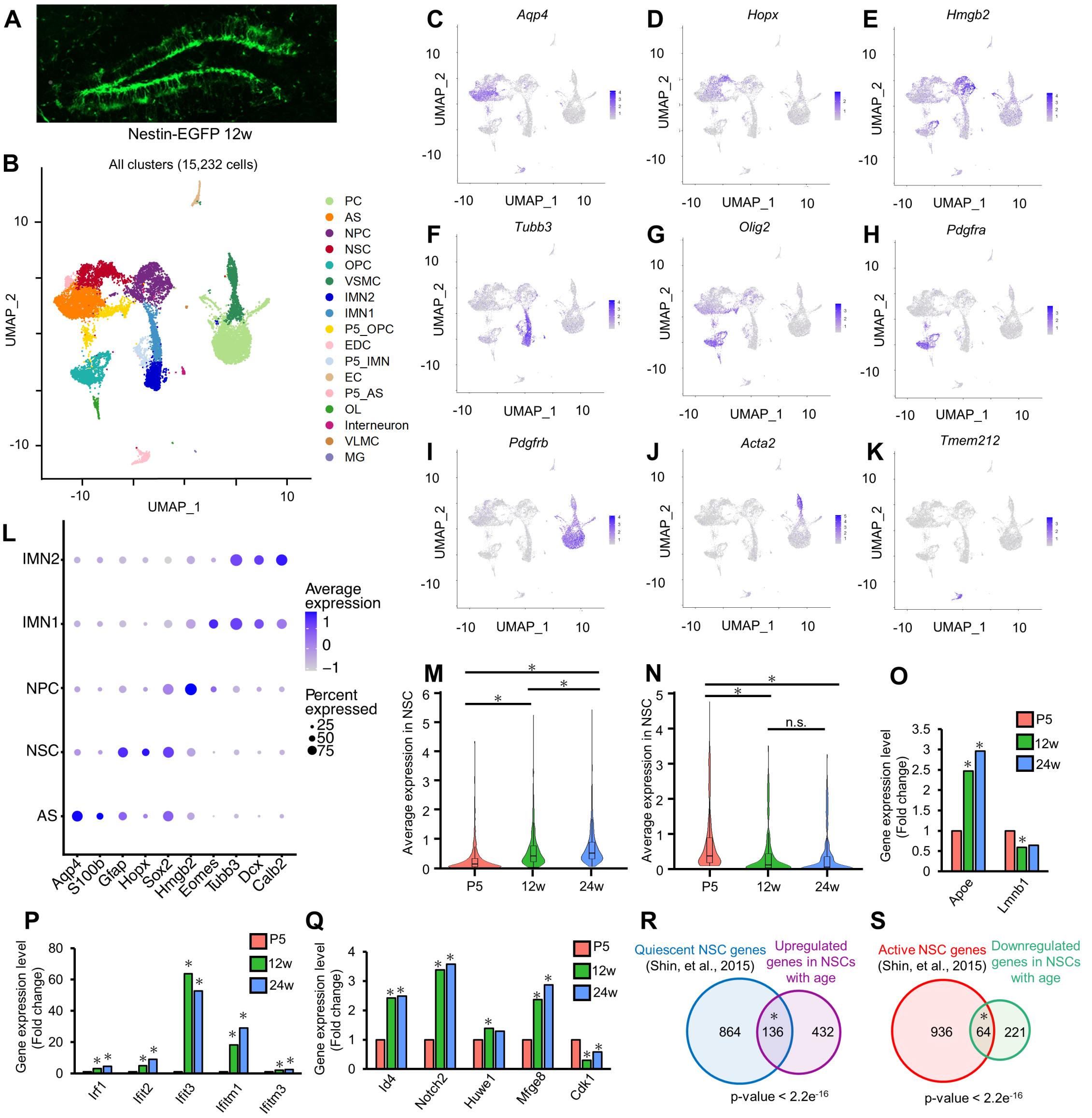

Appendix Figure S1: Clustering results using data for scRNA-seq of Nestin-EGFP+ cells isolated from DGs at P5, 12w, and 24w.

(A) Immunostaining of DG in Nestin-EGFP mice using antibody against GFP (green).

(B) UMAP plot showing all cells including 17 clusters from Nestin-FGFP+ cells including pericytes (PC), astrocytes (AS), neural progenitor cells (NPC), neural stem cells (NSC), oligodendrocyte progenitor cells (OPC), vascular smooth muscle cells (VSMC), immature neurons 2 (IMN2), immature neurons 1 (IMN1), P5-specific oligodendrocyte progenitor cells (P5_OPC), entrepreneurship development cells (EDC), P5-specific immature neurons (P5_IMN), enterochromaffin cells (EC), P5-specific astrocytes (P5_AS), oligodendrocytes (OL), interneurons, vascular and leptomeningeal cells

(VLMC), and microglia (MG).

(C-K) Feature plot showing expression of marker genes, *Aqp4* (AS), *Hopx* (NSC), *Hmgb2* (NPC), *Tubb3* (IMN1 and IMN2), *Olig2* (OPC), *Tmem212* (EDC), *Pdgfrb* (PC), *Acta2* (VSMC), and *Pecam1* (EC).

(L) Heatmap showing expression of marker genes in re-clustered cell populations.

(M) Violin plot showing average expression of upregulated DEGs in NSCs at P5, 12w, and 24w from scRNA-seq dataset. *$p < 0.05$ by ANOVA with Tukey post hoc tests compared with P5.

(N) Violin plot showing average expression of downregulated DEGs in NSCs at P5, 12w, and 24w scRNA-seq dataset. *$p < 0.05$ by ANOVA with Tukey post hoc tests compared with P5.

(O) Bar graph showing fold change of average gene expression related to cellular senescence in NSCs at 12w and 24w compared to P5. *$q < 0.05$ by FindMarker in Seurat package with MAST compared with P5.

(P) Bar graph showing fold change of interferon signaling gene expression in each cluster compared to P5. *$q < 0.05$ by FindMarker in Seurat package with MAST compared with P5.

(Q) Bar graph showing fold change of quiescence-associated gene expression in each cluster compared to P5. *$q < 0.05$ by FindMarker in Seurat package with MAST compared with P5.

(R) Venn diagram showing significant overlap between upregulated DEGs in aged NSCs and NSC quiescence-associated genes reported by Shin et al., in 2015 (Fisher's test).

(S) Venn diagram showing significant overlap between downregulated DEGs in aged NSCs and NSC activation-associated genes, reported by Shin et al., in 2015 (Fisher's test).

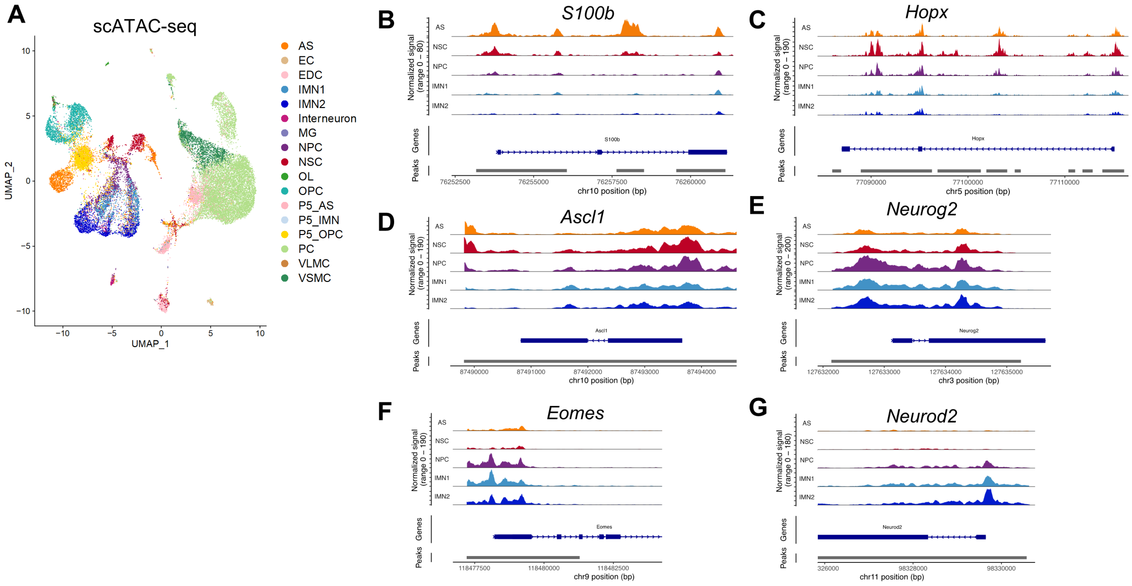

Appendix Figure S2: Clustering results using data for scATAC-seq of the Nestin-EGFP+ cells isolated from DGs at P5, 12w, and 24w.

(A) UMAP plot showing 17 clusters from Nestin-FGFP+ cells including pericytes (PC), astrocytes (AS), neural progenitor cells (NPC), neural stem cells (NSC), oligodendrocyte progenitor cells (OPC), vascular smooth muscle cells (VSMC), immature neurons 2 (IMN2), immature neurons 1 (IMN1), P5-specific oligodendrocyte progenitor cells (P5_OPC), ependymal cells (EDC), P5-specific immature neurons (P5_IMN), endothelial cells (EC), P5-specific astrocytes (P5_AS), oligodendrocytes (OL), interneurons, vascular leptomeningeal cells (VLMC), and microglia (MG).

(B-G) Alignment data for scATAC-seq in NSCs *in vivo* (P5,12w, and 24w) at marker gene loci (*S100β*, *Hopx*, *Ascl1*, *Neurog2*, *Eomes*, and *Neurod2*), showing chromatin openness in the five indicated clusters.

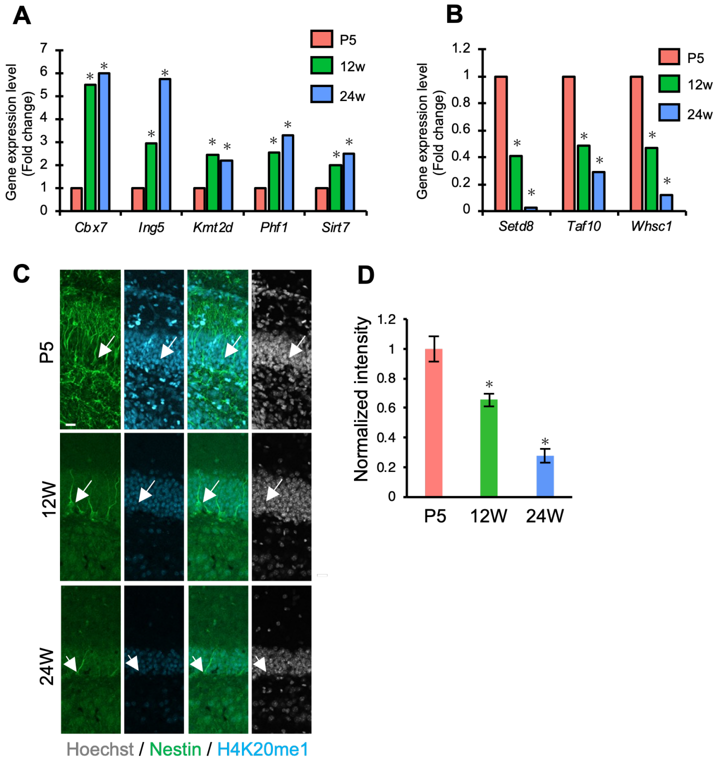

Appendix Figure S3: Setd8 expression and H4K20me1 level during aging process.

(A) Bar graph showing fold change of average expression of upregulated DEGs overlapped with epigenetic regulators compared to P5. *$q < 0.05$ by FindMarker in Seurat package with MAST compared with P5.

(B) Bar graph showing fold change of average expression of downregulated DEGs overlapped with epigenetic regulators compared to P5. *$q < 0.05$ by FindMarker in Seurat package with MAST compared with P5.

(C) Representative images of staining for EGFP (green), H4K20me1 (cyan), and Hoechst (gray) in the hippocampal DG at P5, 12w, and 24w. Arrows indicate Nestin+ NSCs. Scale bar, 20 μm.

(D) Bar graph showing H4K20me1 fluorescence intensity normalized by background in NSCs, with the fluorescence intensity at P5 set to 1 (n = 30 cells from three mice per group). *$p < 0.01$ by Wilcoxon rank-sum test.

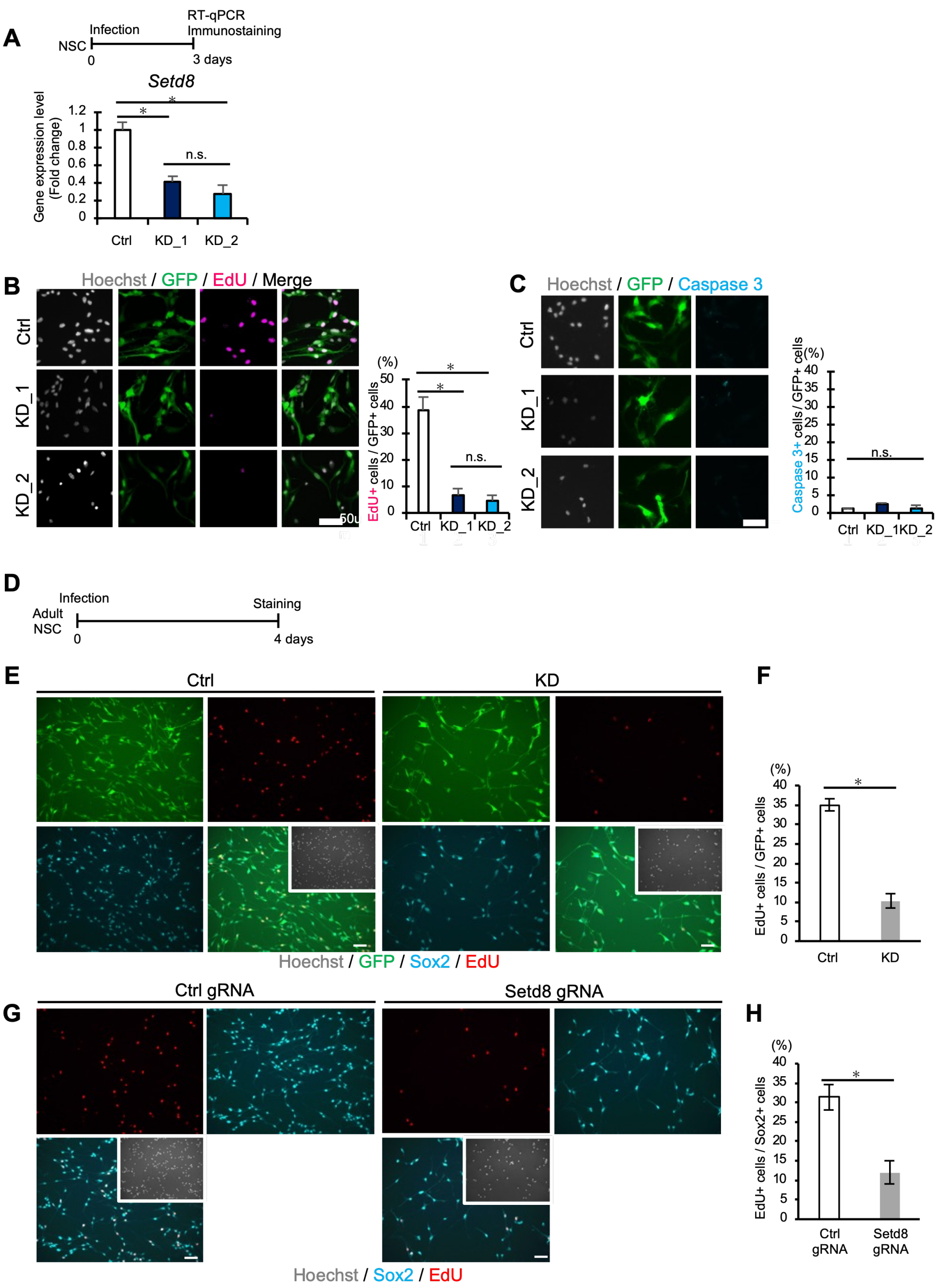

Appendix Figure S4: Setd8 downregulation impairs cultured NSC proliferation.

(A) Experimental scheme for investigating Setd8 function of NSCs *in vitro* (n = 5). Bar graph showing the expression levels of *Setd8* knockdown (KD) 1, KD 2, or Ctrl virus-infected mouse NSCs compared to Ctrl by RT-qPCR (n = 3). **p* < 0.05 by ANOVA with Tukey post hoc tests. n.s., not significant.

(B) Representative images of GFP (green), EdU (magenta), and Hoechst (gray) staining

in Setd8-KD or Ctrl virus-infected cultured mouse NSCs. Scale bar, 50 µm. Right bar graph showing quantification of EdU+ cells in GFP+ cells (n = 5). **p* < 0.05 by ANOVA with Tukey post hoc tests. n.s., not significant.

(C) Representative images of GFP (green), active Caspase 3 (cyan), and Hoechst (gray) staining in Setd8-KD or Ctrl virus-infected cultured mouse NSCs. Scale bar, 50 µm. Right bar graph showing quantification of active Caspase 3+ cells in GFP+ cells (n = 5). n.s., not significant.

(D) Experimental scheme for assessing the effect of *Setd8* KD (E and F) or KO (G and H) on proliferation of adult hippocampal NSCs *in vitro*.

(E) Representative images of staining for GFP (green), Sox2 (cyan), EdU (red), and Hoechst (gray) at 3 days after Ctrl or *Setd8* KD viral infection. Scale bar, 50 µm.

(F) Proportion of GFP+ EdU+ cells among GFP+ cells (E) at 3 days after Ctrl or *Setd8* KD viral infection (n = 4 each). **p* < 0.01 by Student's t test.

(G) Representative images of staining for Sox2 (cyan), EdU (red), and Hoechst (gray) at 3 days after infection with a vector containing both Cas9 and *Setd8* gRNA, or a control vector, followed by puromycin selection.

(H) Proportion of EdU+ Sox2+ cells among Sox2+ cells (G) at 3 days after Cas9 and gRNA viral infection (n = 4 each). **p* < 0.01 by Student's t test.

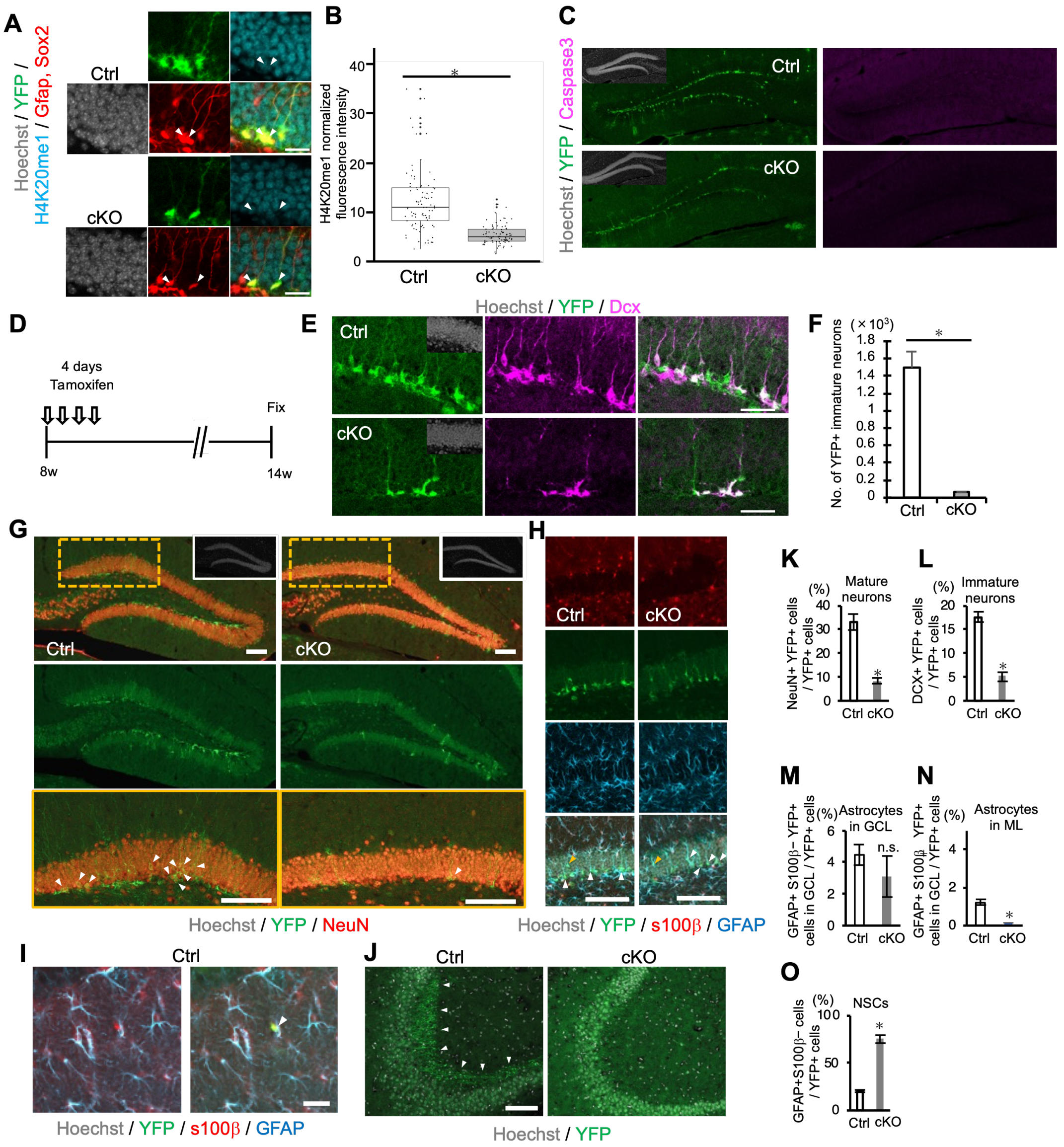

Appendix Figure S5: Conditional knockout of *Setd8* impairs neurogenesis in the hippocampus.

(A) Representative confocal images of staining for YFP (green), H4K20me1 (cyan), Sox2 (red), Gfap (red), and Hoechst (gray) in Ctrl and cKO. Arrowheads indicate soma of NSCs. Scale bars, 20 µm.

(B) Box plots showing H4K20me1 fluorescence intensity in NSCs normalized by background intensity in Ctrl and cKO mice (86 cells in three mice per group). **p* < 0.05 by Student's t test.

(C) Representative tile scan confocal images of YFP (green), active Caspase 3 (magenta), and Hoechst (gray; insets) in the DG of 8-week-old Ctrl and cKO mice one day after last

tamoxifen administration. Scale bars, 100 μm.

(D) Experimental scheme for investigating Setd8 function in adult neurogenesis. six weeks after last administration of tamoxifen to 8-weeks-old mice, we performed immunostaining DGs of Ctrl and cKO.

(E) Representative images of YFP (green), Dcx (magenta), and Hoechst (gray; insets) in the DG of 14-week-old Ctrl and cKO mice. Scale bars, 40 μm.

(F) Quantification of YFP+ and Dcx+ newly generated neurons in Ctrl and cKO mice (n = 4 animals per group). *$p < 0.01$ by Student's t test.

(G) Representative images of staining for YFP (green), NeuN (red), and Hoechst (gray; insets) of the DGs in Ctrl (n = 4 animals) and *Setd8* cKO (n = 4 animals). Arrowheads indicate YFP-labeled mature neurons generated from NSCs. Bottom panels show higher magnification of dashed boxes in top panels. Scale bar, 100 μm.

(H and I) Representative images of staining for YFP (green), S100β (red), GFAP (cyan), and Hoechst (gray) of the DGs in Ctrl (n = 4 animals) and *Setd8* cKO (n = 4 animals). White and yellow arrowheads indicate YFP+ GFAP+ S100β– NSCs in the SGZ (H) and YFP+ GFAP+ S100β– astrocytes in the granular cell layer (GCL) (H), respectively. White arrowheads indicate YFP+ GFAP+ S100β+ astrocytes in the molecular layer (ML) (I). Scale bar, 50 μm.

(J) Representative images of staining for projection of YFP+ newly generated neurons into the CA3 region. White arrowheads highlight YFP+ cell fiber. Scale bar, 100 μm.

(K-O) Proportion of YFP+ NeuN+ mature neurons (K), YFP+ DCX+ immature neurons (L), and YFP+ GFAP+ S100β– astrocytes in the GCL (M), YFP+ GFAP+ S100β+ astrocytes in the ML (N), or YFP+ GFAP+ S100β– NSCs (O) among YFP+ cells in the DG (n = 4 animals per group). *$p < 0.05$ by Student's t test.

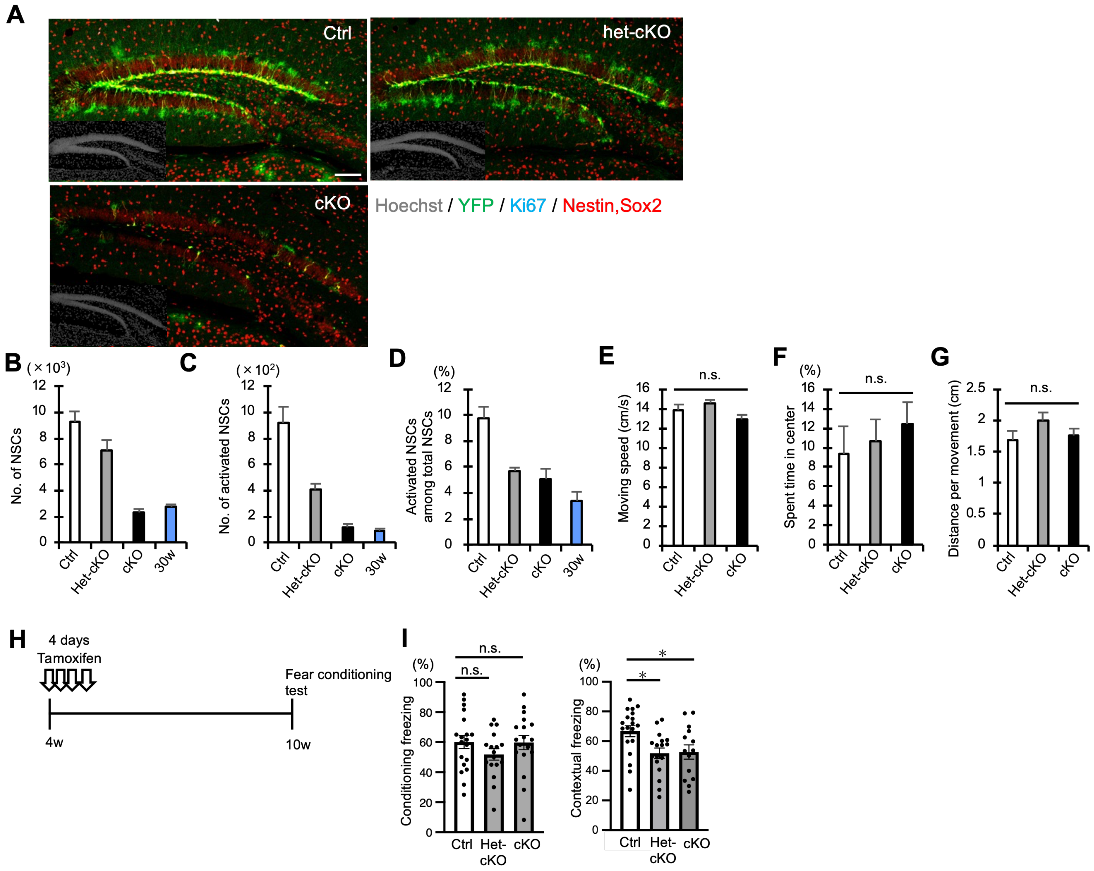

Appendix Figure S6: *Setd8* deletion induces premature aging of NSCs in the DG without affecting locomotor function.

(A) Representative tile scan confocal images (upper) of staining for YFP (green), Ki67 (cyan), Nestin (red), Sox2 (red), and Hoechst (gray; insets) DGs in Ctrl, het-cKO, and cKO mice. Scale bar, 100 µm.

(B) Quantification of NSCs expressing YFP in Ctrl, het-cKO, and cKO mice and NSCs in 30-week-old WT (30w) mice (n = 4 animals per group).

(C) Quantification of activated NSCs expressing YFP in Ctrl, het-cKO, and cKO mice and activated NSCs in 30-week-old WT (30w) mice (n = 4 animals per group).

(D) Bar graph showing the proportion of activated NSCs in total NSCs in Ctrl, het-cKO, and cKO mice and 30-week-old WT (30w) mice (n = 4 animals per group).

(E-G) Bar graphs showing moving speed at sampling (E), time spent in center (F), and distance per movement (G) (Ctrl (n = 14 animals), het-cKO (n = 18 animals), and cKO (n = 13 animals)). n.s., not significant, $p \geq 0.05$ by ANOVA with Tukey post hoc tests.

(H) Experimental scheme for contextual fear conditioning test at young adult age (10w).

(I) Quantification of freezing rate in the conditioning (left graph) or testing (right graph) phase. *$p < 0.05$ by ANOVA with Tukey post-hoc tests. n.s., not significant.

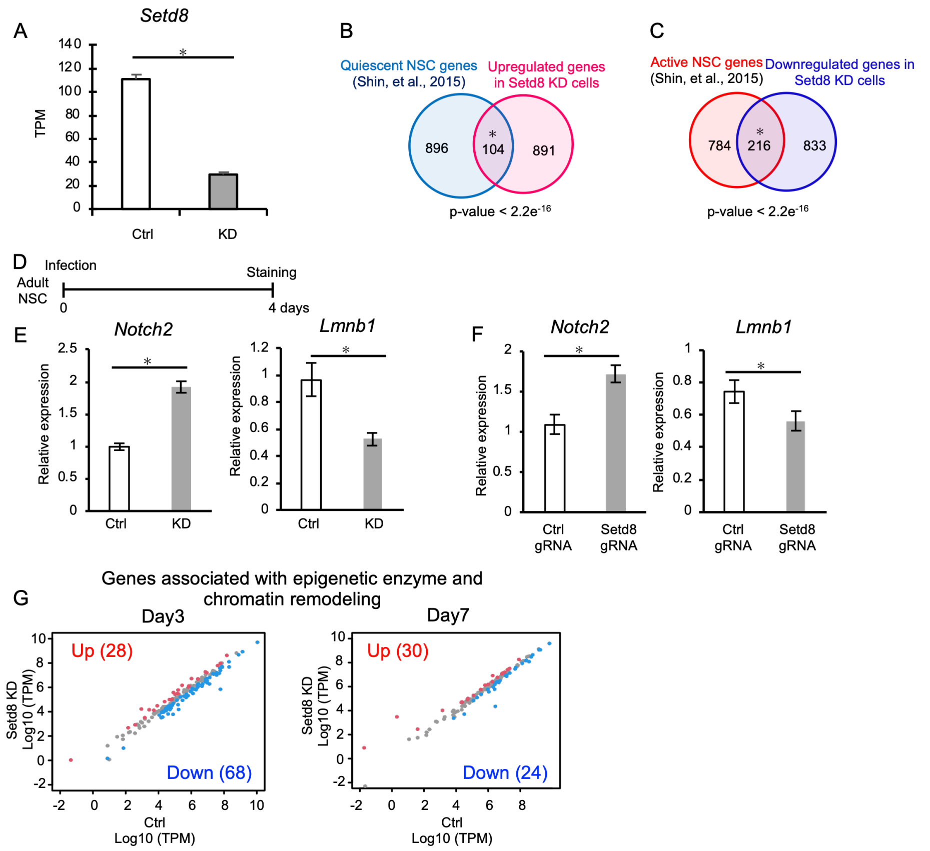

Appendix Figure S7: Altered transcriptomic characteristics in NSCs by Setd8 downregulation.

(A) Bar graph showing TPM of *Setd8* in Ctrl (white) and KD (gray). *$q < 0.01$.

(B) Venn diagram showing significant overlap between upregulated DEGs by Setd8-KD and NSC quiescence-associated genes reported by Shin *et al.*, 2015 (Fisher's test).

(C) Venn diagrams showing significant overlap between downregulated DEGs by Setd8-KD and NSC activation-associated genes reported by Shin *et al.*, 2015 (Fisher's test).

(D) Experimental scheme for assessing the effect of *Setd8* KD (E) or KO (F) on proliferation of adult hippocampal NSCs *in vitro*.

(E,F) qRT-PCR analysis of *Notch2* and *Lmnb1* mRNA levels in *Setd8* KD (E) or KO (F)

NSCs (n = 4). *$p < 0.05$ by Student's t test.

(G) Scatter plots showing the expression levels of genes associated with epigenetic modification and chromatin remodeling (n = 171) in control and *Setd8* KD NSCs at day 3 (left) and day 7 (right)**.** Up- (red) and downregulated (blue) differentially expressed genes are highlighted.

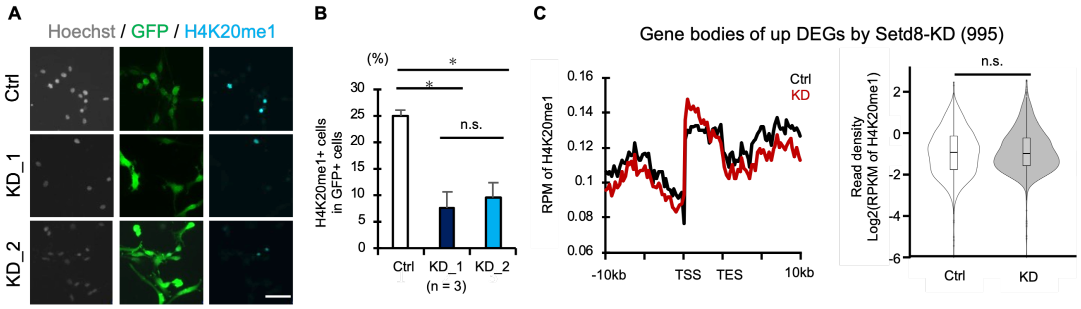

Appendix Figure S8: Effect of Setd8 knockdown on H4K20me1 modification in cultured NSCs.

(A) Representative images of GFP (green), H4K20me1 (cyan), and Hoechst (gray) staining in Setd8-KD or Ctrl virus-infected mouse NSCs. Scale bar, 50 µm.

(B) Bar graph showing quantification of H4K20me1+ cells in GFP+ cells (n = 5). *$p <$ 0.05 by ANOVA with Tukey post hoc tests. n.s., not significant.

(C) Enrichment profile of H4K20me1 around gene bodies of upregulated DEGs by Setd8-KD in Ctrl (black) and KD (red) (left). Violin plot showing no difference in RPKM of H4K20me1 enrichment in gene bodies of upregulated DEGs between Setd8-KD and Ctrl (right). n.s., not significant, $p \geq 0.05$ by Wilcoxon rank-sum test.

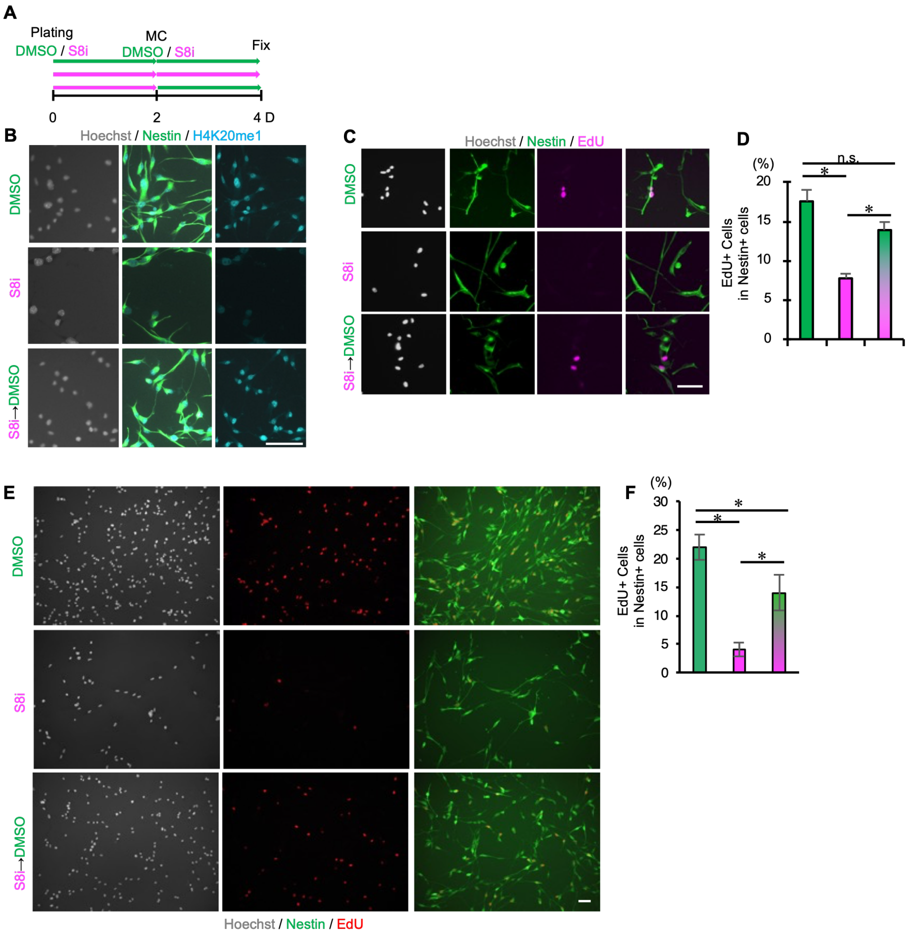

Appendix Figure S9: Effects of Setd8 inhibitor on NSC proliferation.

(A)Schematic representation of the experimental timeline. NSCs derived from embryonic cortex with repeated passages (B and C) and adult hippocampus (E and F) were plated and treated with either DMSO (control) or the Setd8 inhibitor S8i (5 µM) for 2 days, followed by maintenance in either DMSO or S8i for an additional 2 days before fixation.

(B)Representative immunofluorescence images of NSCs stained for Nestin (green) and H4K20me1 (cyan) with Hoechst counterstaining (gray) under different treatment conditions: DMSO, S8i, and S8i withdrawal (DMSO→S8i). Scale bar: 50 µm.

(C)Representative immunofluorescence images showing Nestin (green) and EdU incorporation (magenta) with Hoechst counterstaining (gray) to assess proliferation of NSCs under different treatment conditions. Scale bar: 50 µm.

(D)Quantification of EdU+ cells among Nestin+ NSCs in panel (C). Data are presented as mean ± SEM. Statistical significance was determined using a one-way ANOVA followed by post-hoc tests (*$p < 0.05$, n.s.: not significant).

(E)Representative immunofluorescence images showing Nestin (green) and EdU incorporation (red) with Hoechst counterstaining (gray) to assess proliferation of adult hippocampal NSCs under different treatment conditions. Scale bar: 50 μm.

(F)Quantification of EdU+ cells among Nestin+ adult hippocampal NSCs in panel (E). Data are presented as mean ± SEM. Statistical significance was determined using a one-way ANOVA followed by post-hoc tests (*$p < 0.05$).
