# Supplementary figures and images for: Epigenetic regulation of neural stem cell aging in the mouse hippocampus by Setd8 downregulation (part 1 of 2)
Source: EMBO J. 2025 Jun 3;44(13):3645–68. doi: 10.1038/s44318-025-00455-8 (PMC12218407; doi:10.1038/s44318-025-00455-8)

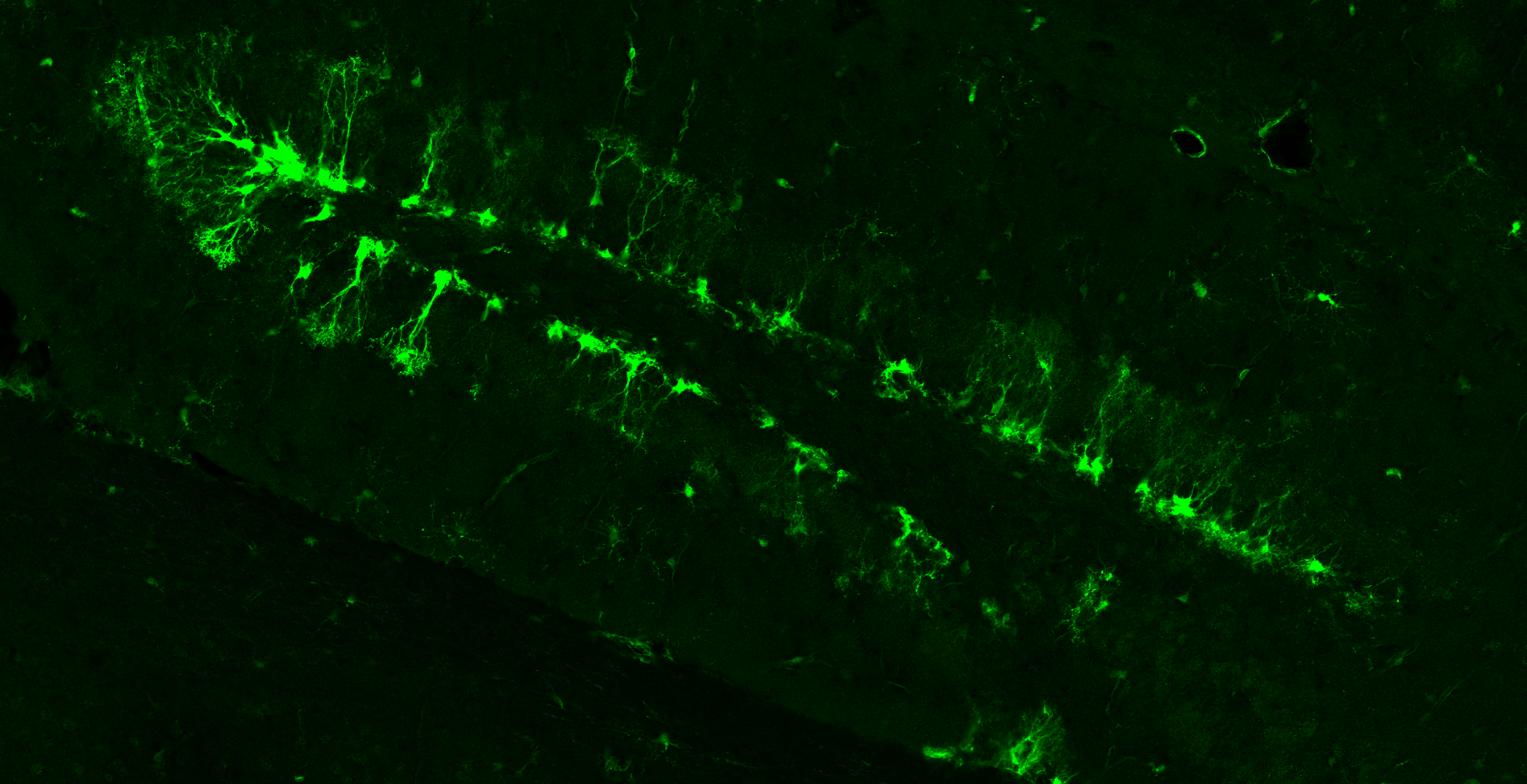

Supplement: Supplementary file 8 — Source data Fig. 3 [file 44318_2025_455_MOESM8_ESM.zip › 3C/24W/original/EGFP.tif]

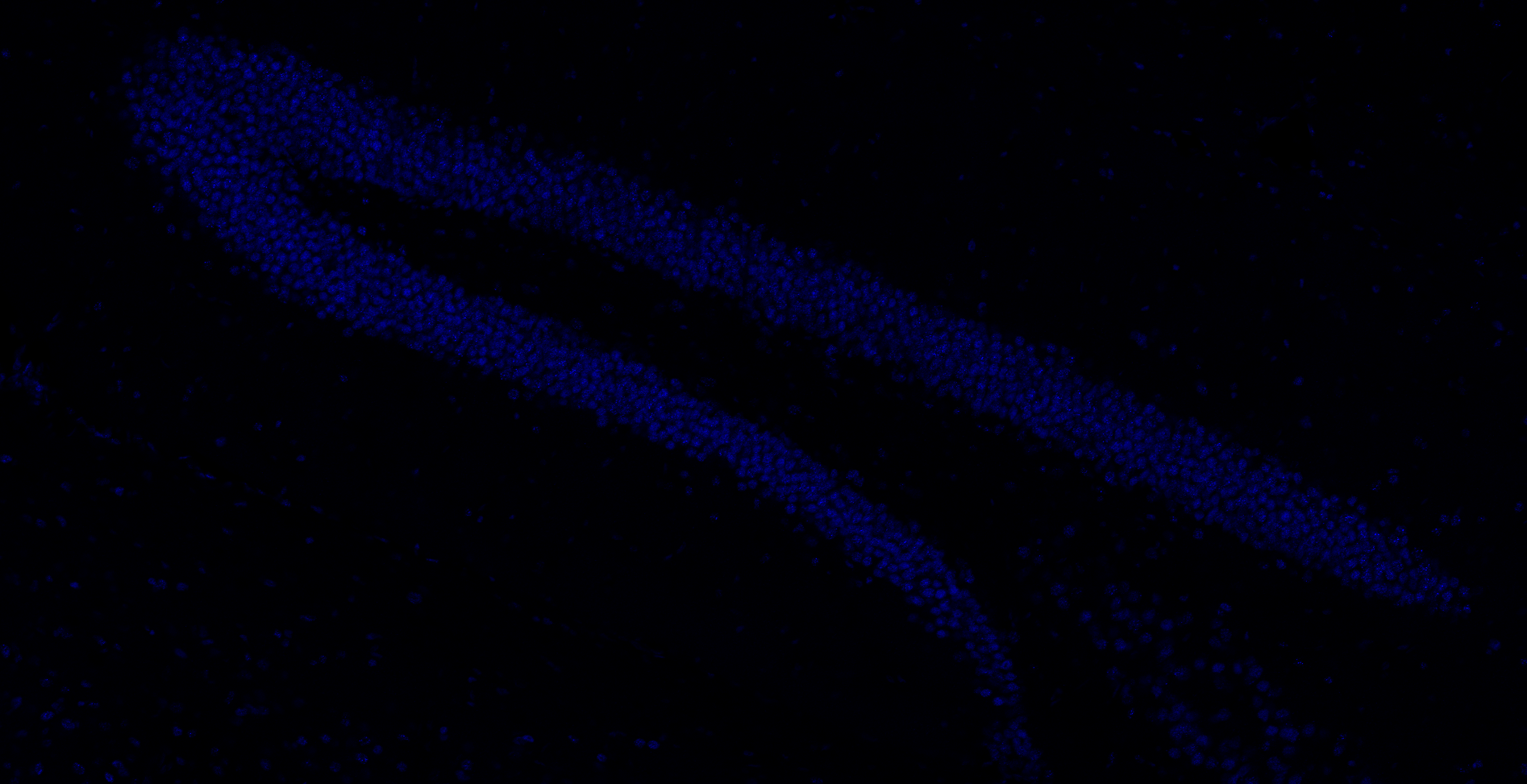

Supplement: Supplementary file 8 — Source data Fig. 3 [file 44318_2025_455_MOESM8_ESM.zip › 3C/24W/original/H4K20me1.tif]

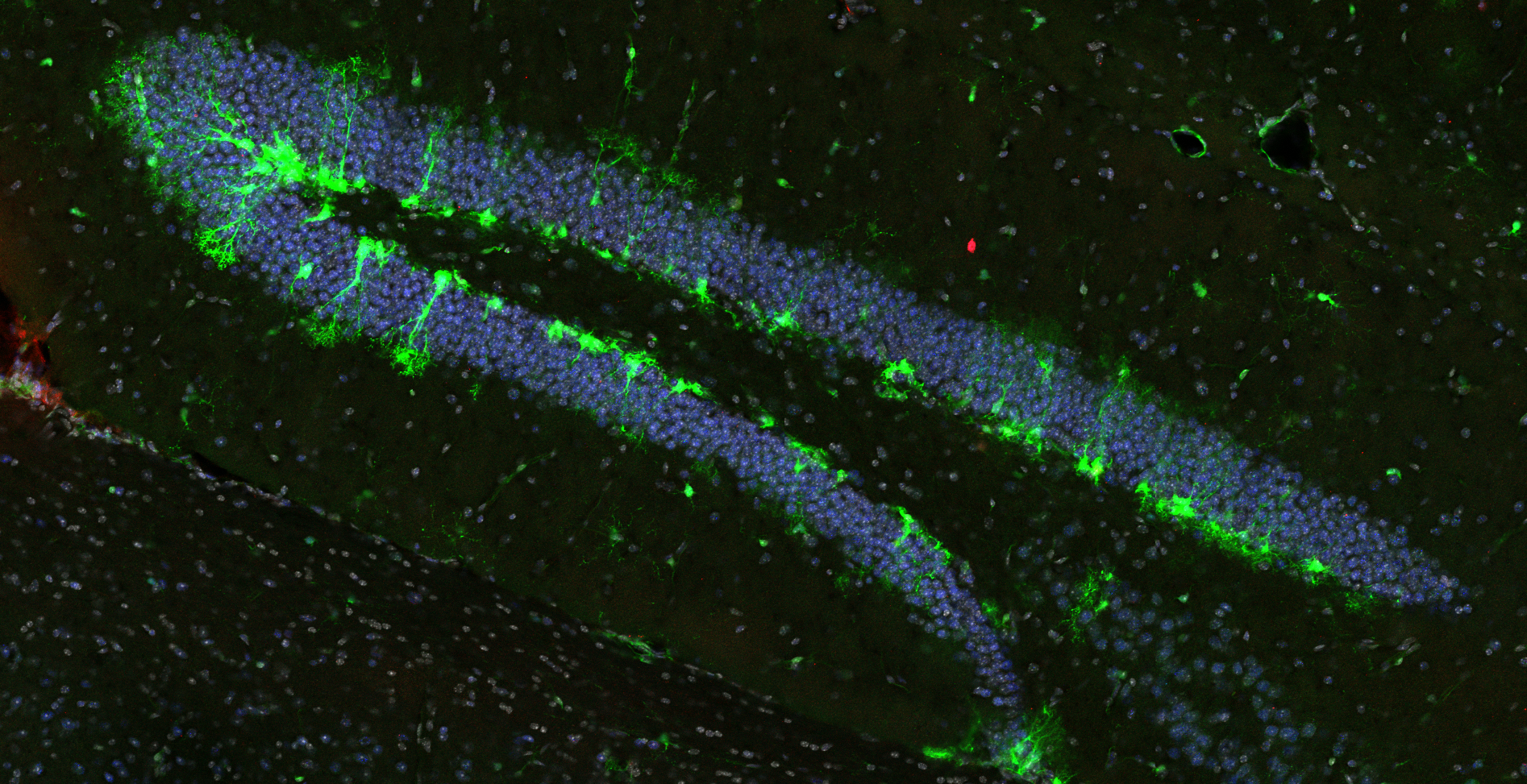

Supplement: Supplementary file 8 — Source data Fig. 3 [file 44318_2025_455_MOESM8_ESM.zip › 3C/24W/original/Marge.tif]

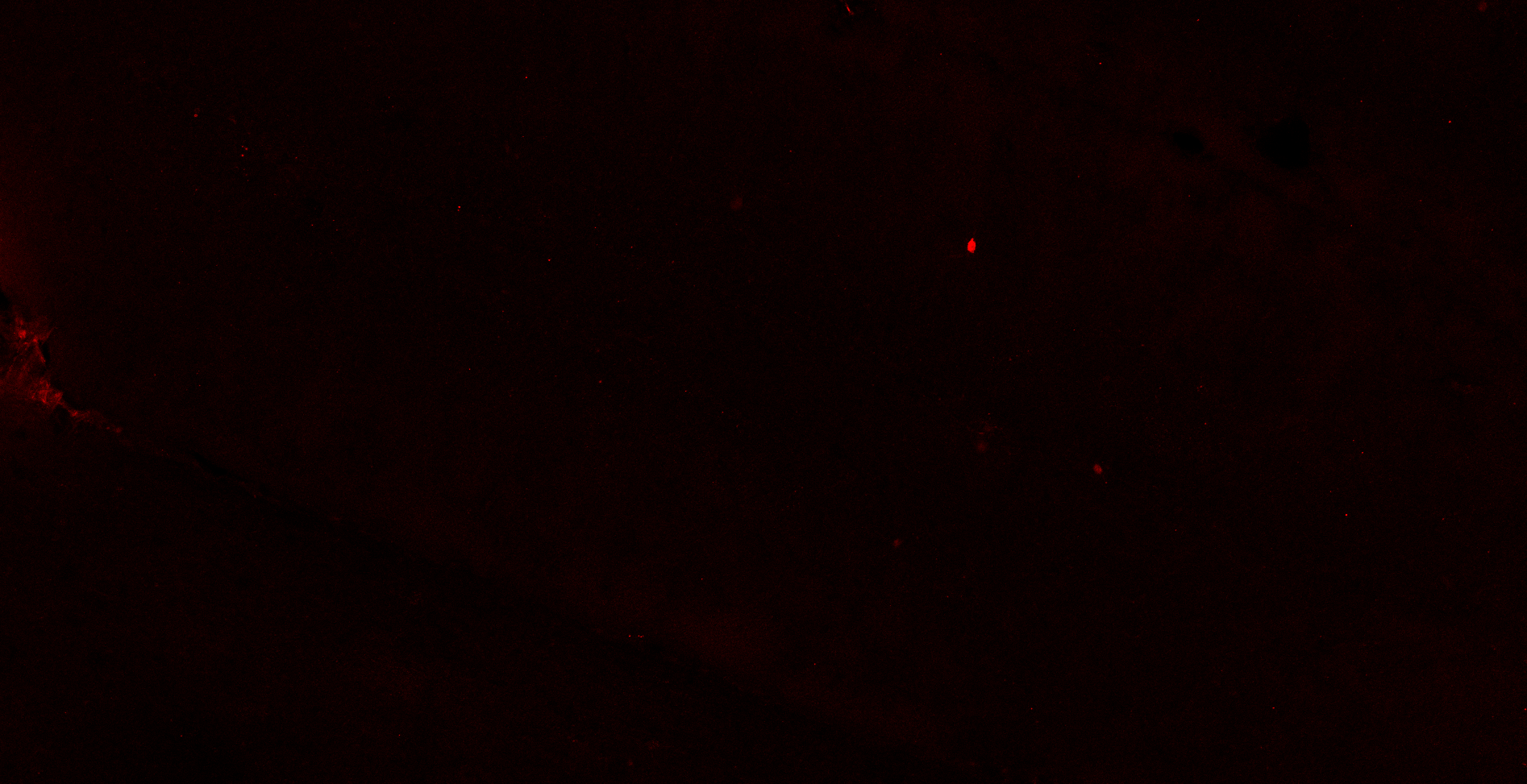

Supplement: Supplementary file 8 — Source data Fig. 3 [file 44318_2025_455_MOESM8_ESM.zip › 3C/24W/original/Ki67.tif]

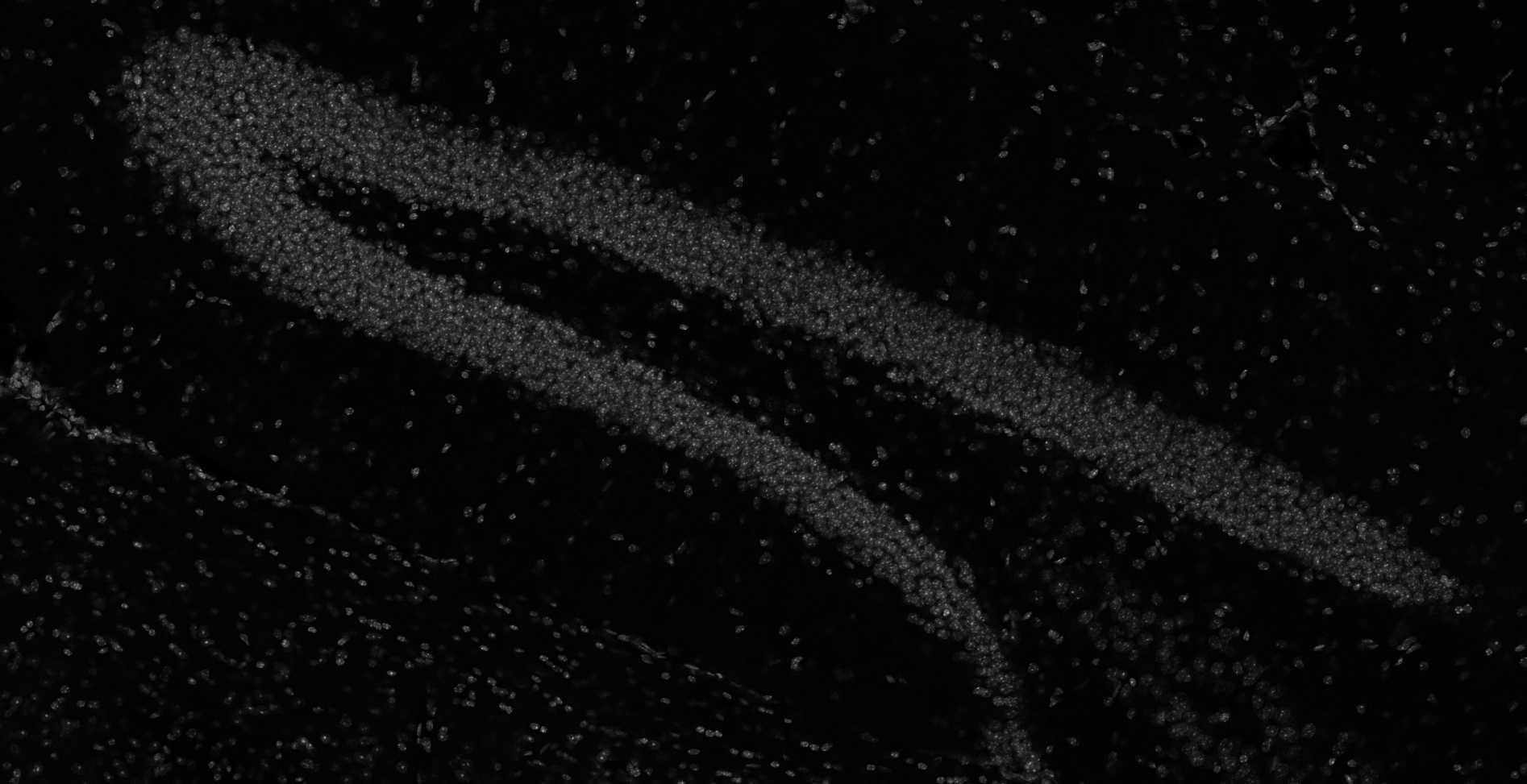

Supplement: Supplementary file 8 — Source data Fig. 3 [file 44318_2025_455_MOESM8_ESM.zip › 3C/24W/original/Hoechst.tif]

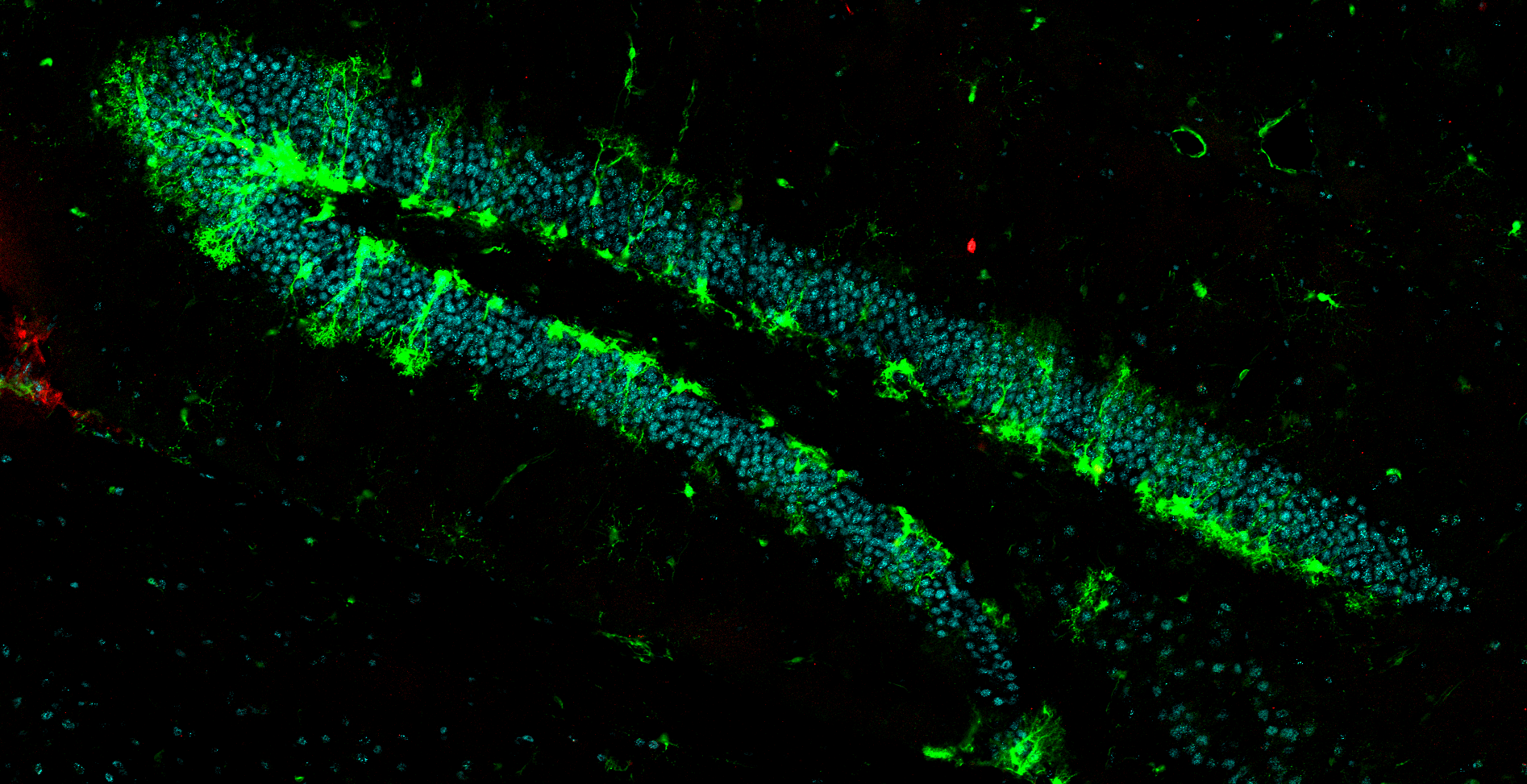

Supplement: Supplementary file 8 — Source data Fig. 3 [file 44318_2025_455_MOESM8_ESM.zip › 3C/24W/edited/Marge.tif]

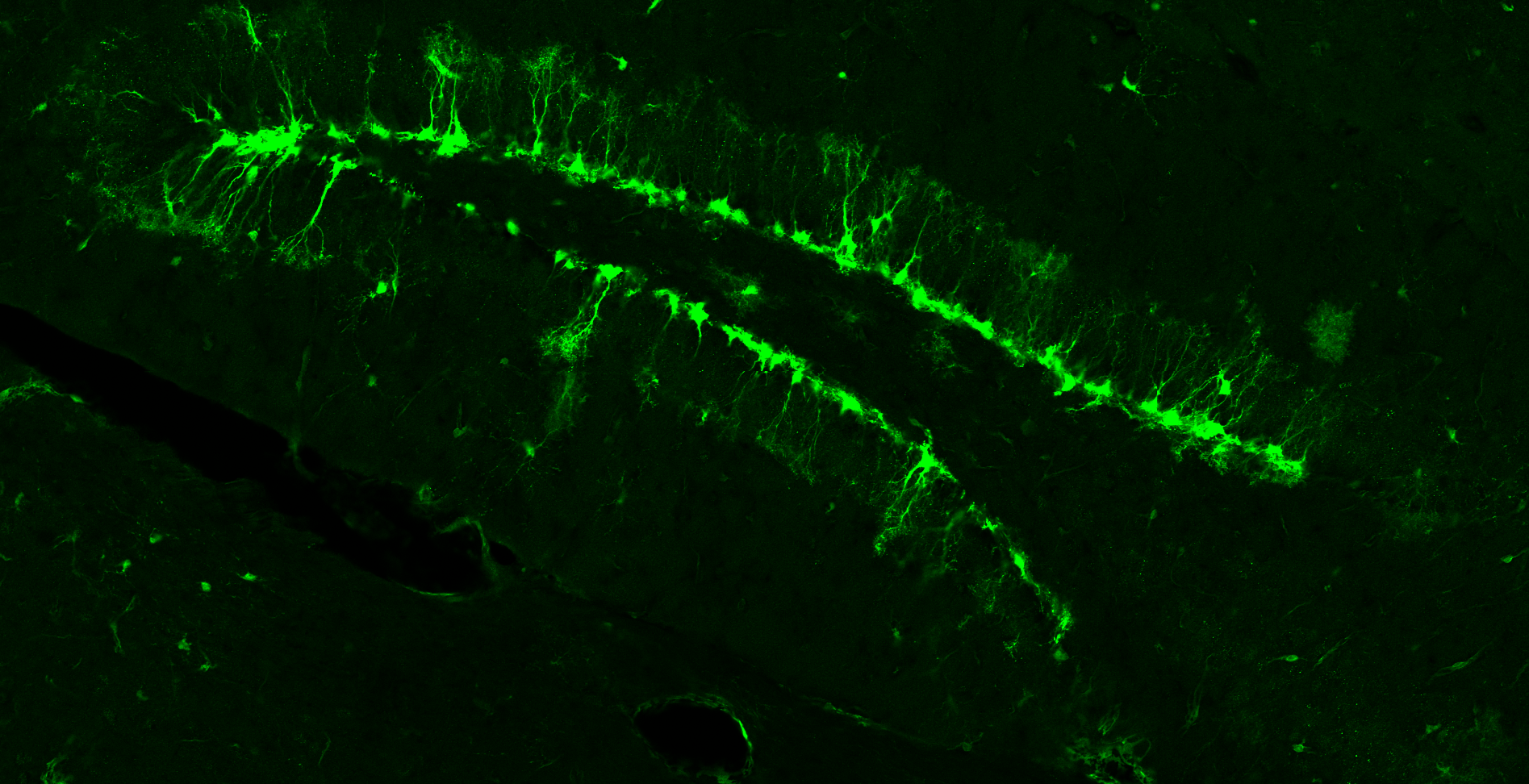

Supplement: Supplementary file 8 — Source data Fig. 3 [file 44318_2025_455_MOESM8_ESM.zip › 3C/12W/original/EGFP.tif]

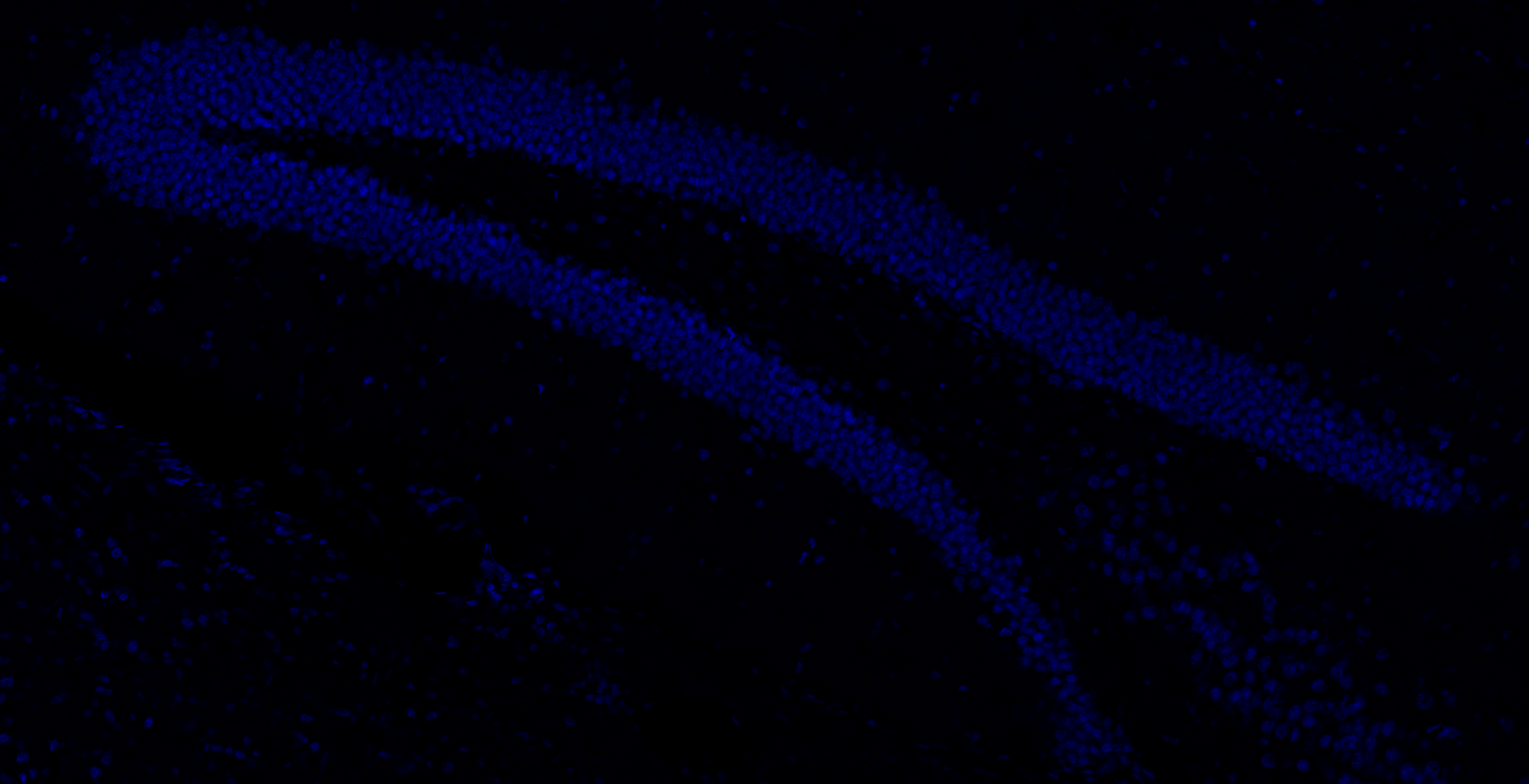

Supplement: Supplementary file 8 — Source data Fig. 3 [file 44318_2025_455_MOESM8_ESM.zip › 3C/12W/original/H4K20me1.tif]

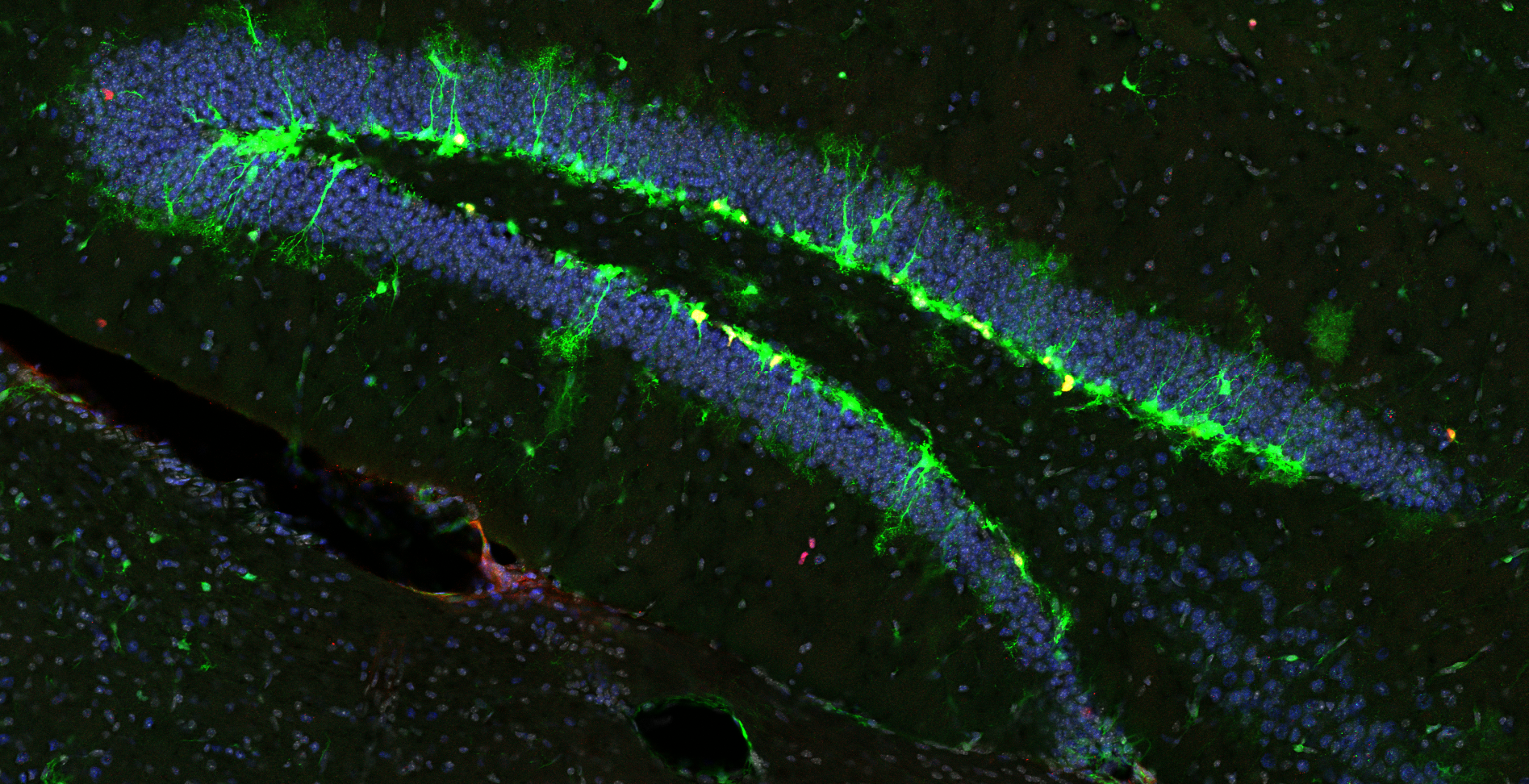

Supplement: Supplementary file 8 — Source data Fig. 3 [file 44318_2025_455_MOESM8_ESM.zip › 3C/12W/original/Marge.tif]

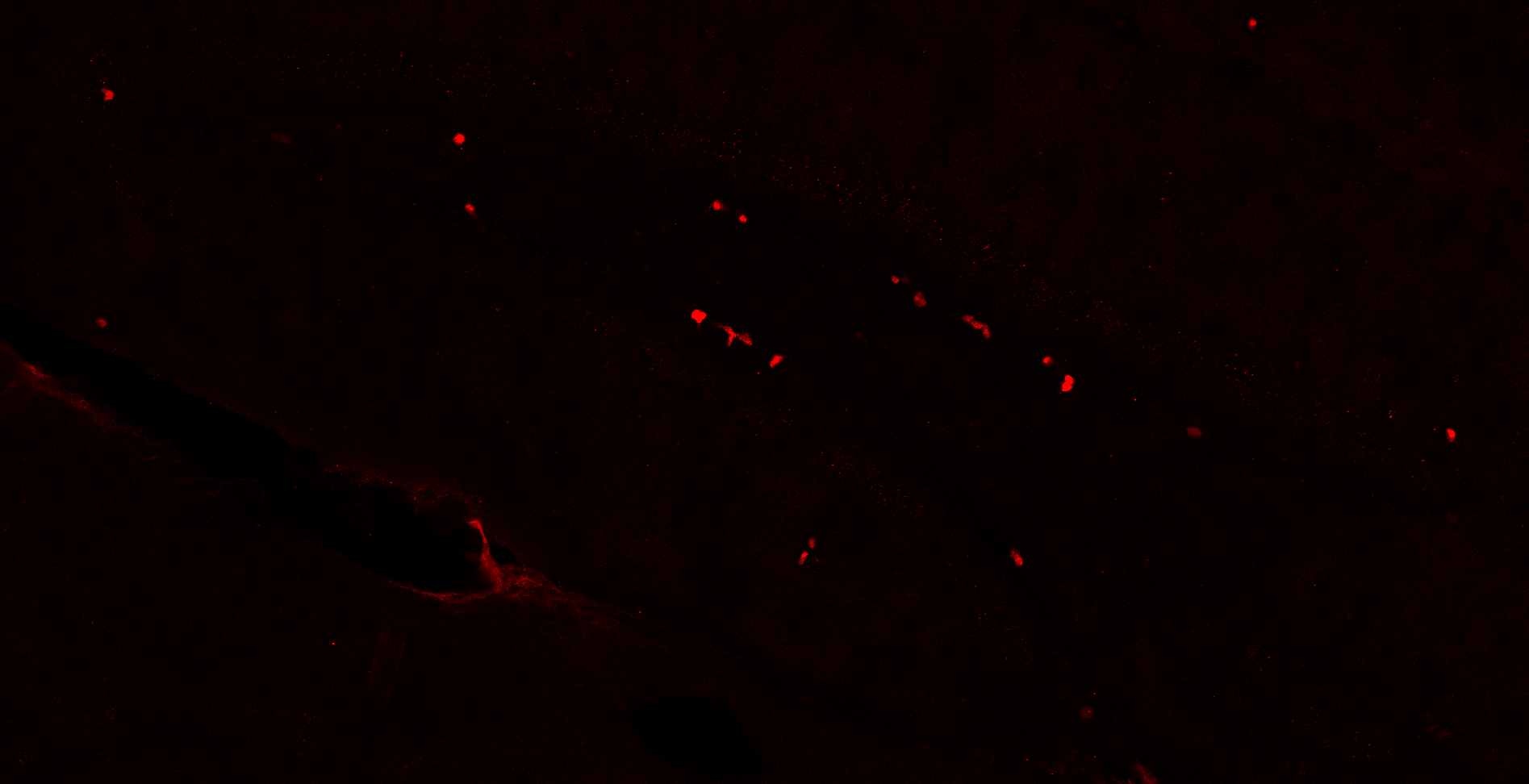

Supplement: Supplementary file 8 — Source data Fig. 3 [file 44318_2025_455_MOESM8_ESM.zip › 3C/12W/original/Ki67.tif]

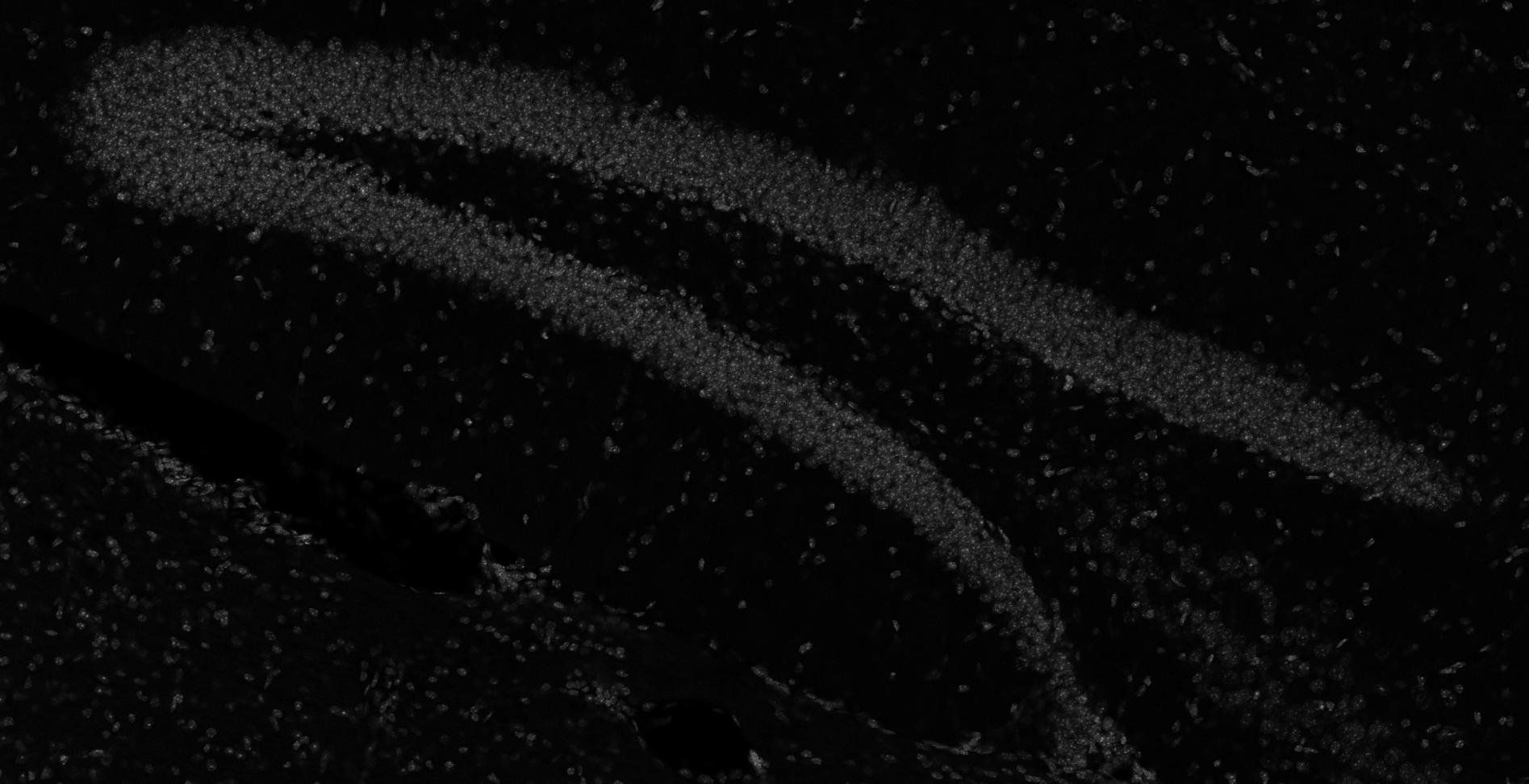

Supplement: Supplementary file 8 — Source data Fig. 3 [file 44318_2025_455_MOESM8_ESM.zip › 3C/12W/original/Hoechst.tif]

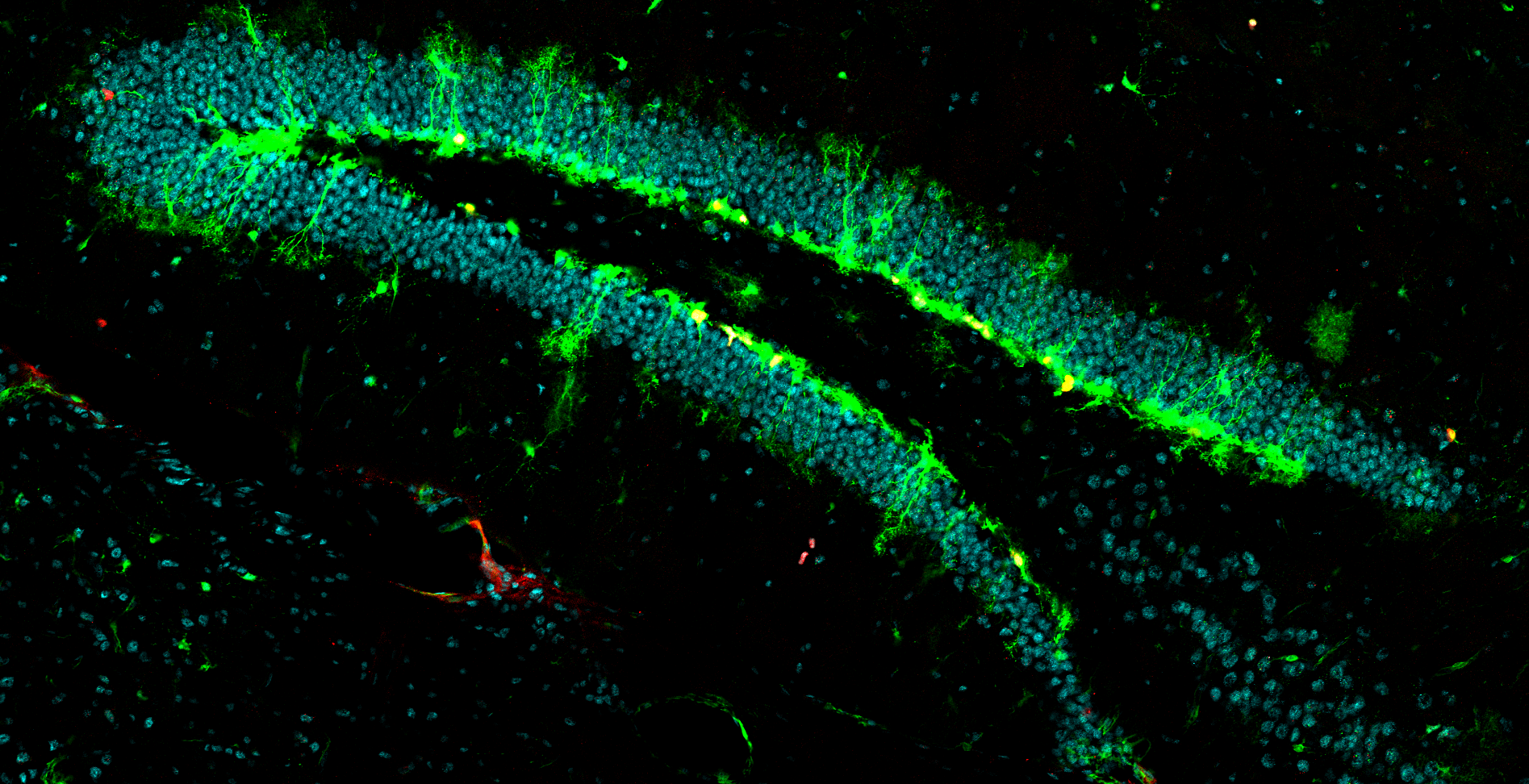

Supplement: Supplementary file 8 — Source data Fig. 3 [file 44318_2025_455_MOESM8_ESM.zip › 3C/12W/edited/Marge.tif]

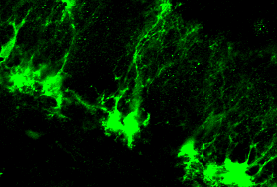

Supplement: Supplementary file 8 — Source data Fig. 3 [file 44318_2025_455_MOESM8_ESM.zip › 3C/24W/edited/magnified/EGFP.tif]

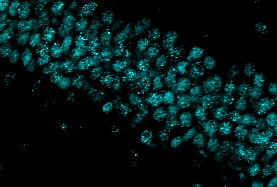

Supplement: Supplementary file 8 — Source data Fig. 3 [file 44318_2025_455_MOESM8_ESM.zip › 3C/24W/edited/magnified/H4K20me1.tif]

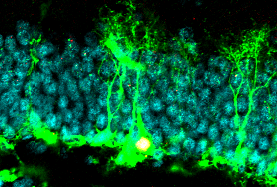

Supplement: Supplementary file 8 — Source data Fig. 3 [file 44318_2025_455_MOESM8_ESM.zip › 3C/24W/edited/magnified/Marge.tif]

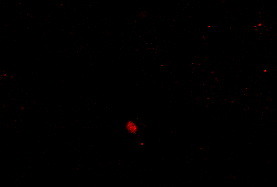

Supplement: Supplementary file 8 — Source data Fig. 3 [file 44318_2025_455_MOESM8_ESM.zip › 3C/24W/edited/magnified/Ki67.tif]

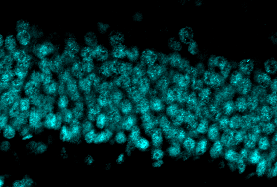

Supplement: Supplementary file 8 — Source data Fig. 3 [file 44318_2025_455_MOESM8_ESM.zip › 3C/12W/edited/magnified/H4K20me1.tif]

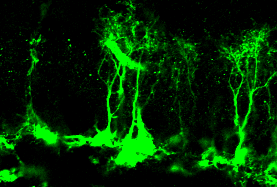

Supplement: Supplementary file 8 — Source data Fig. 3 [file 44318_2025_455_MOESM8_ESM.zip › 3C/12W/edited/magnified/GFP.tif]

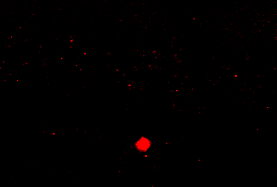

Supplement: Supplementary file 8 — Source data Fig. 3 [file 44318_2025_455_MOESM8_ESM.zip › 3C/12W/edited/magnified/Ki67.tif]

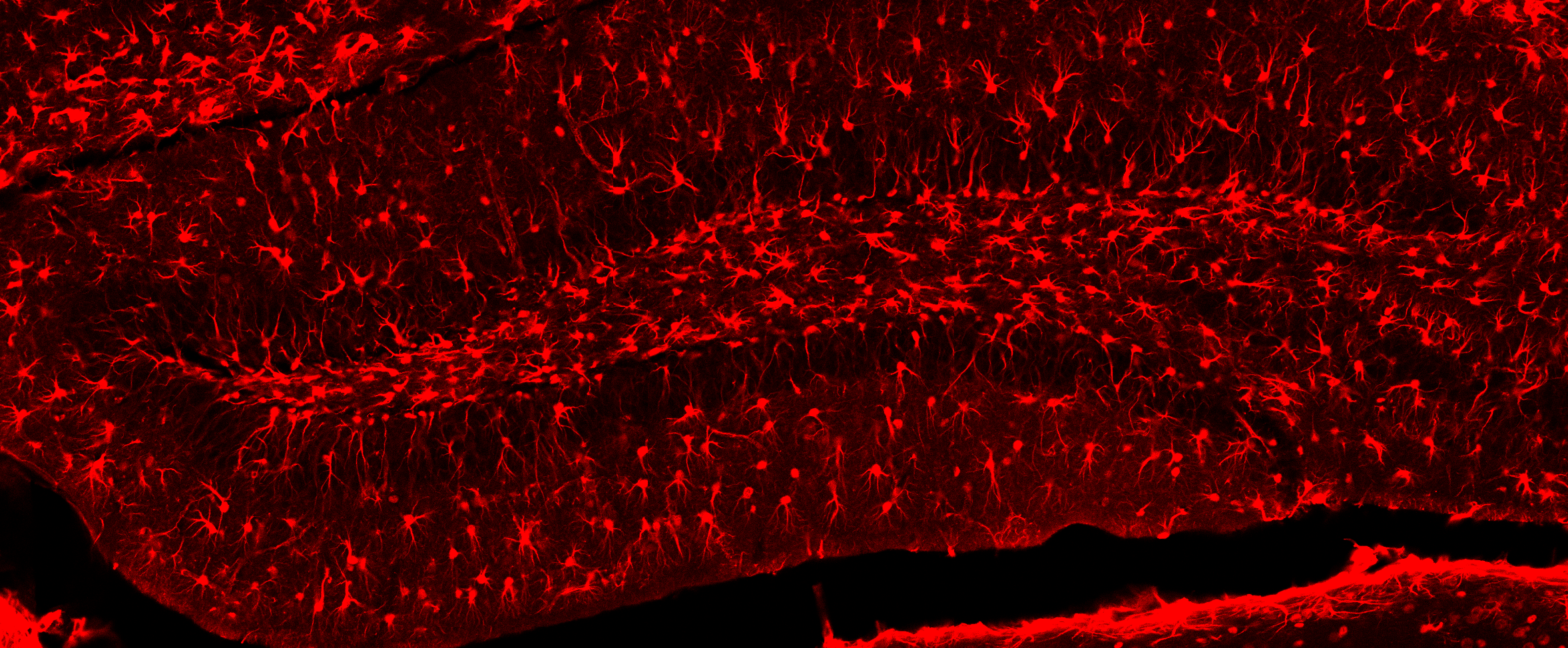

Supplement: Supplementary file 8 — Source data Fig. 3 [file 44318_2025_455_MOESM8_ESM.zip › 3F/cKO/original/Gfap_Sox2.tif]

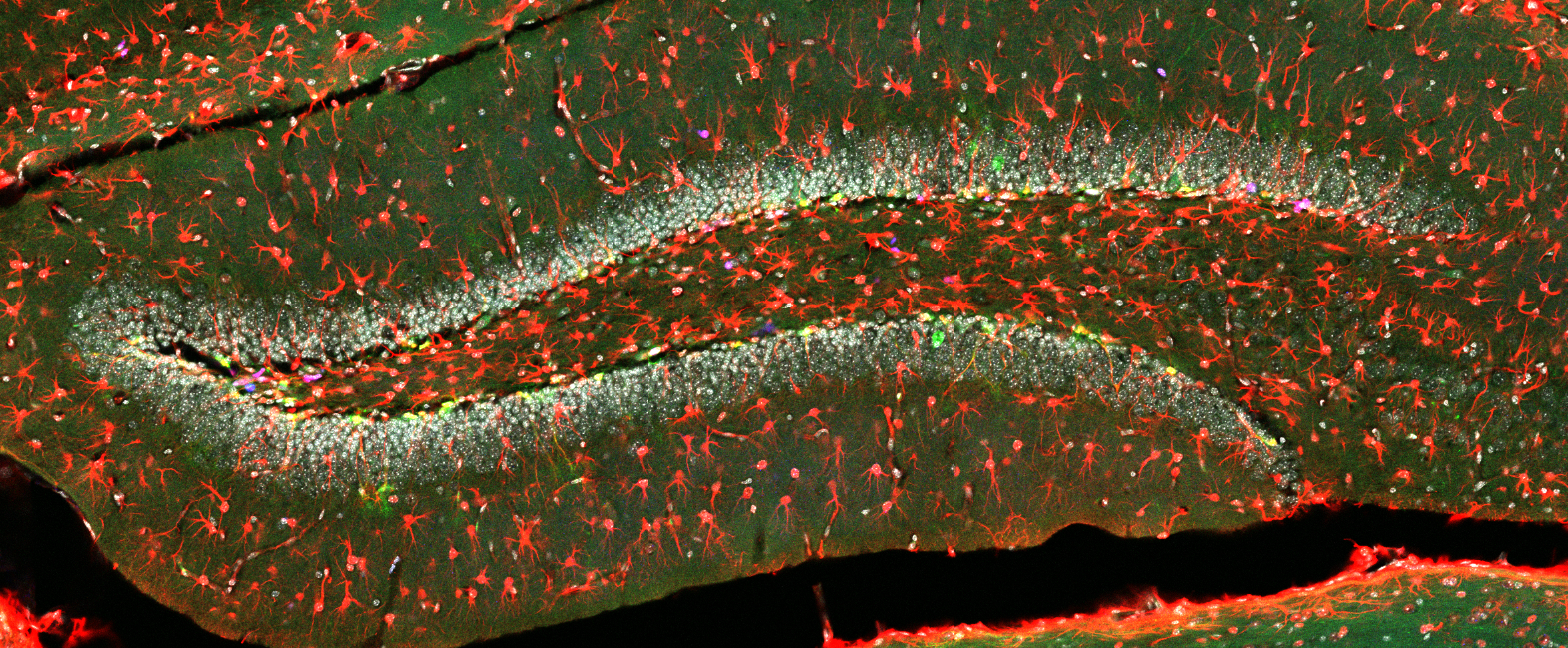

Supplement: Supplementary file 8 — Source data Fig. 3 [file 44318_2025_455_MOESM8_ESM.zip › 3F/cKO/original/Marge.tif]

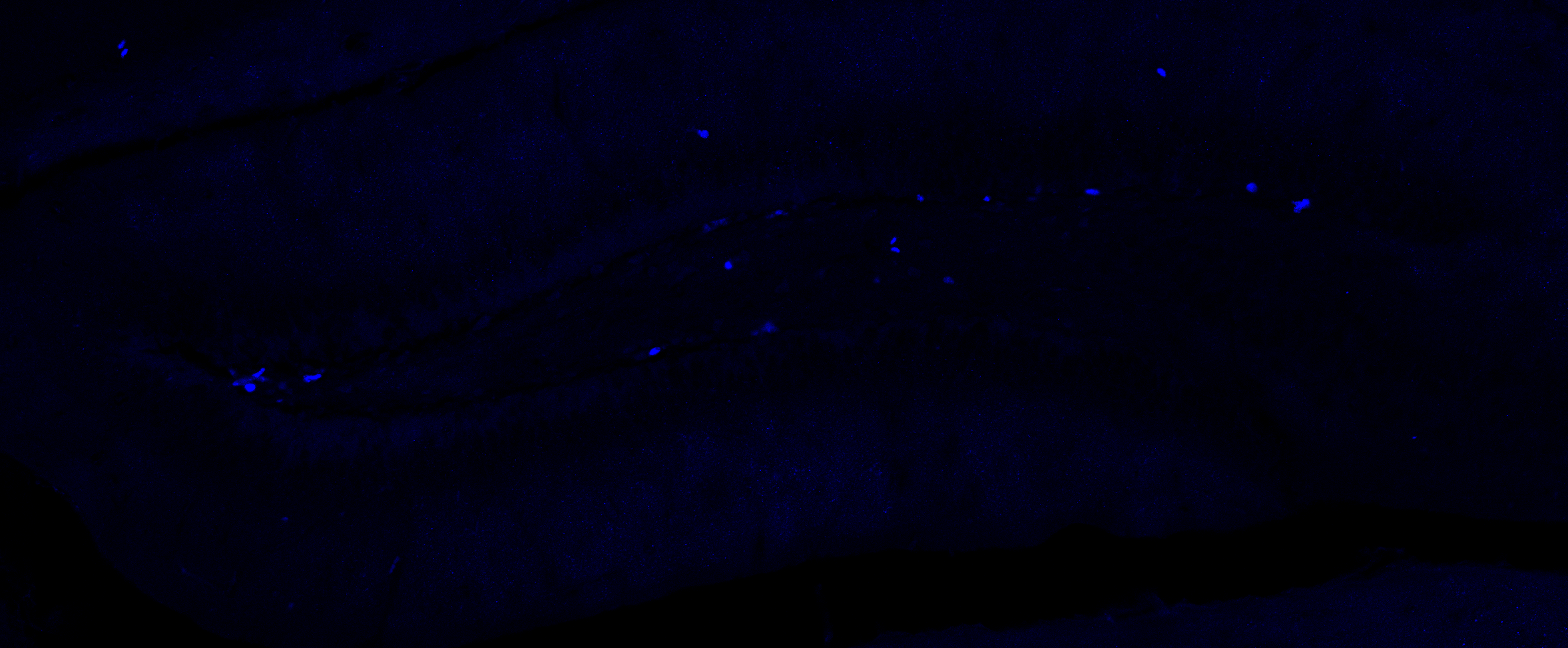

Supplement: Supplementary file 8 — Source data Fig. 3 [file 44318_2025_455_MOESM8_ESM.zip › 3F/cKO/original/Ki67.tif]

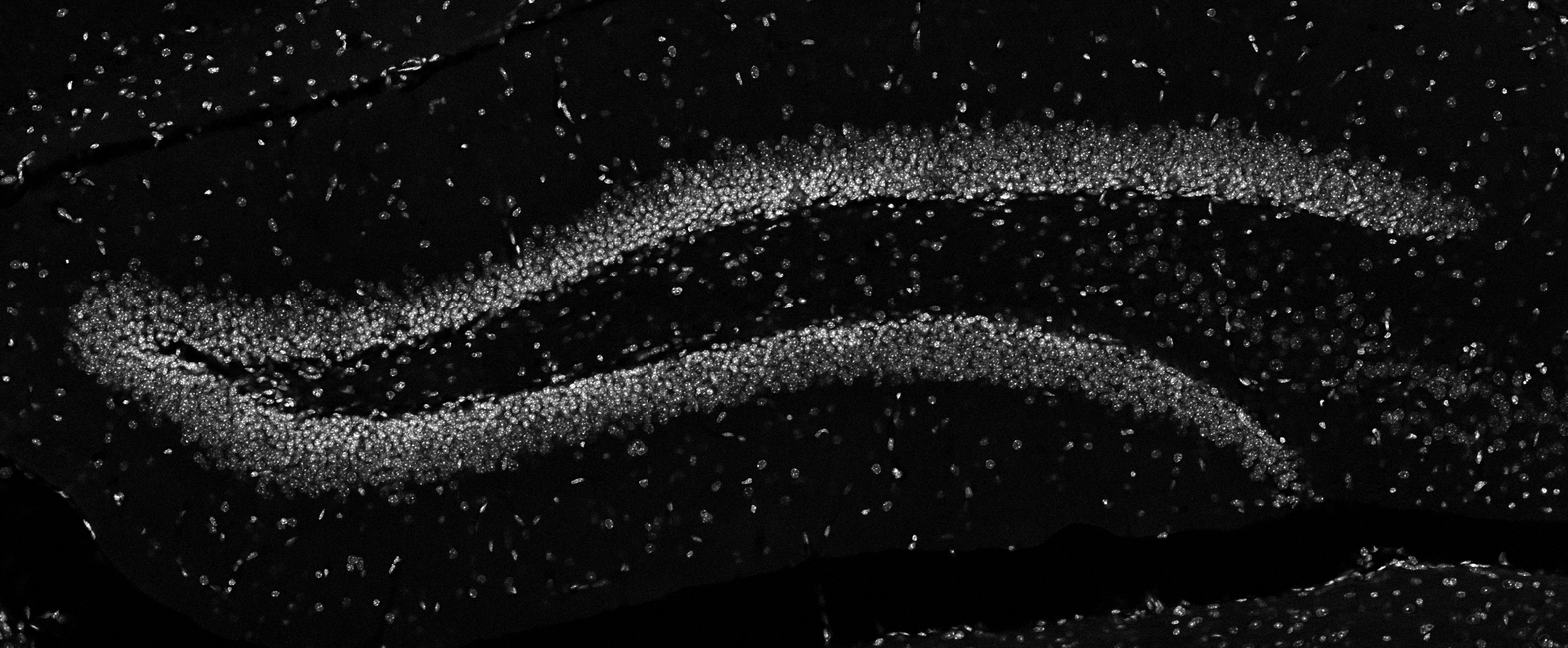

Supplement: Supplementary file 8 — Source data Fig. 3 [file 44318_2025_455_MOESM8_ESM.zip › 3F/cKO/original/Hoechst.tif]

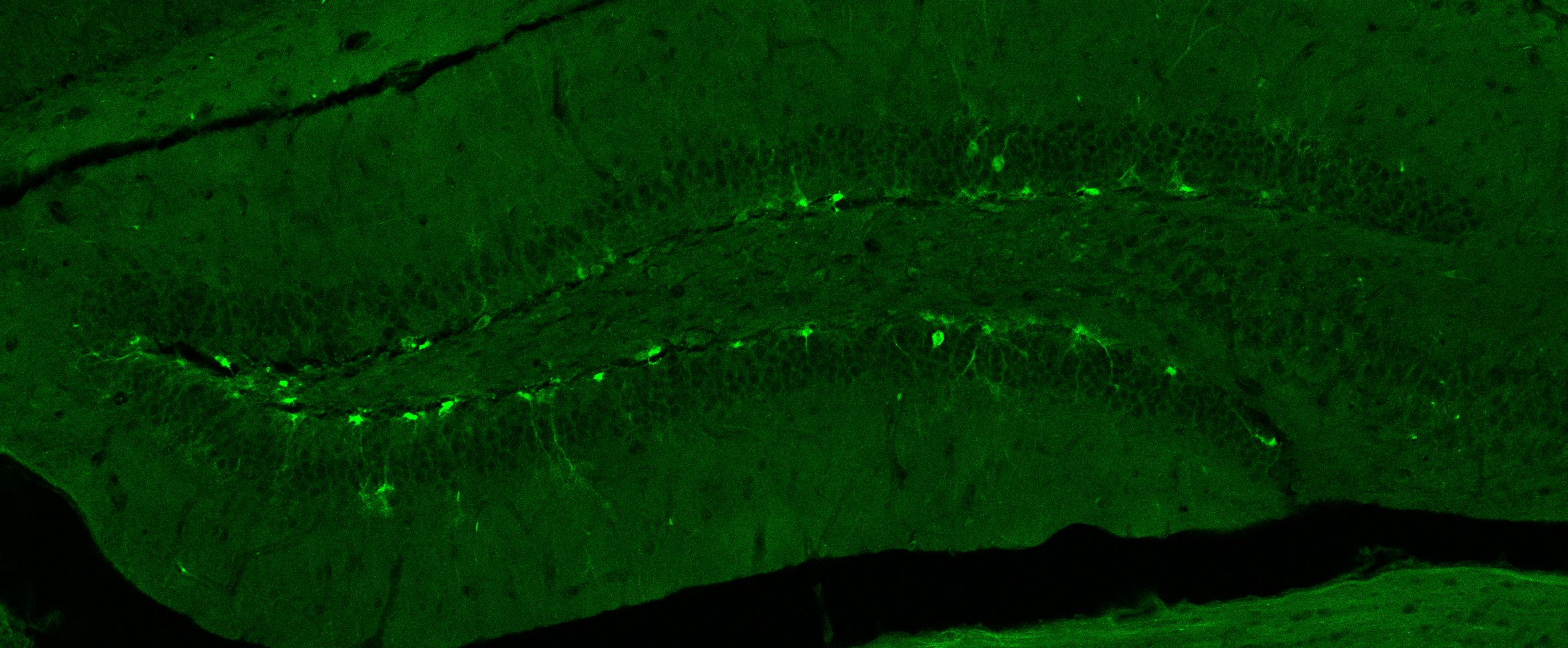

Supplement: Supplementary file 8 — Source data Fig. 3 [file 44318_2025_455_MOESM8_ESM.zip › 3F/cKO/original/YFP.tif]

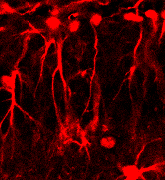

Supplement: Supplementary file 8 — Source data Fig. 3 [file 44318_2025_455_MOESM8_ESM.zip › 3F/cKO/edited/Gfap_Sox2.tif]

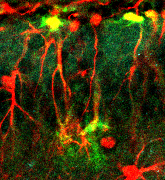

Supplement: Supplementary file 8 — Source data Fig. 3 [file 44318_2025_455_MOESM8_ESM.zip › 3F/cKO/edited/Marge.tif]

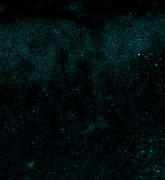

Supplement: Supplementary file 8 — Source data Fig. 3 [file 44318_2025_455_MOESM8_ESM.zip › 3F/cKO/edited/Ki67.tif]

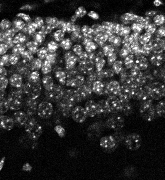

Supplement: Supplementary file 8 — Source data Fig. 3 [file 44318_2025_455_MOESM8_ESM.zip › 3F/cKO/edited/Hoechst.tif]

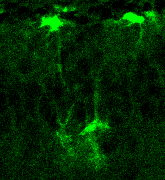

Supplement: Supplementary file 8 — Source data Fig. 3 [file 44318_2025_455_MOESM8_ESM.zip › 3F/cKO/edited/YFP.tif]

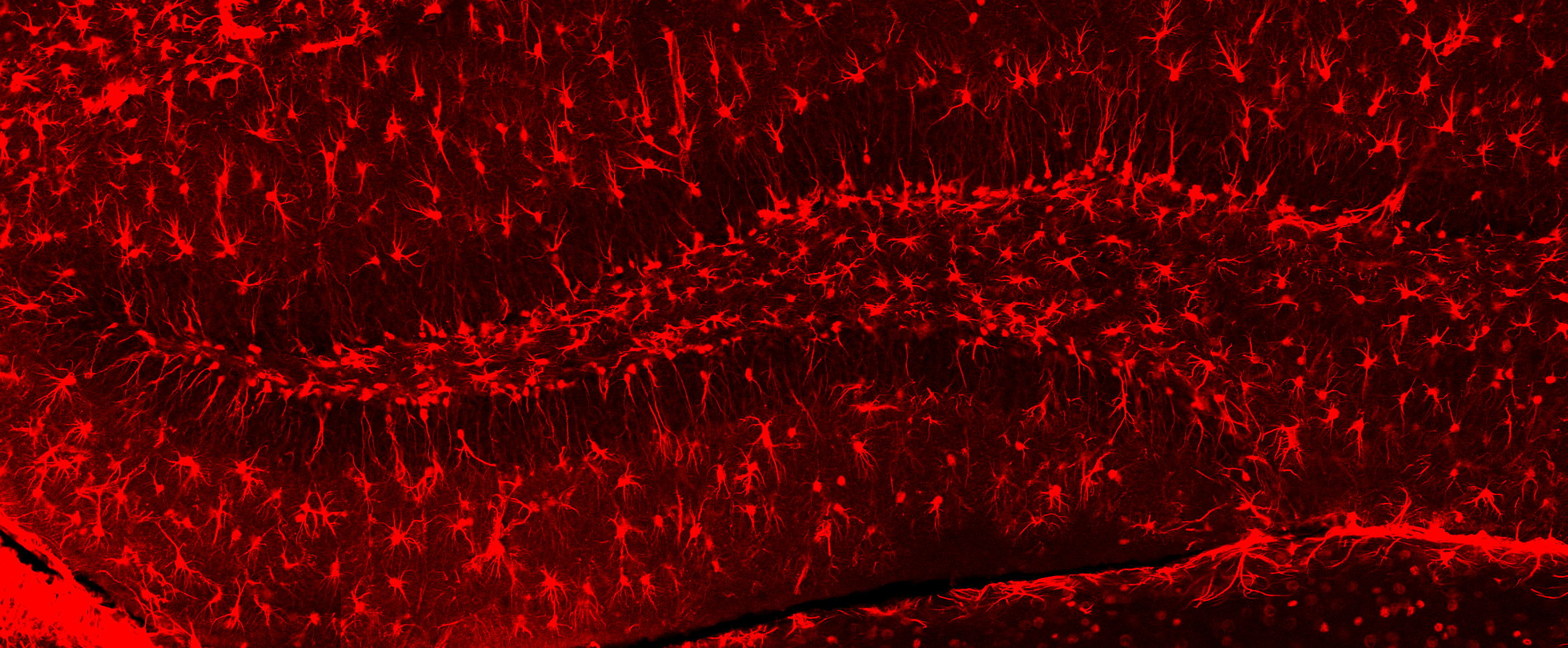

Supplement: Supplementary file 8 — Source data Fig. 3 [file 44318_2025_455_MOESM8_ESM.zip › 3F/Ctrl/original/Gfap_Sox2.tif]

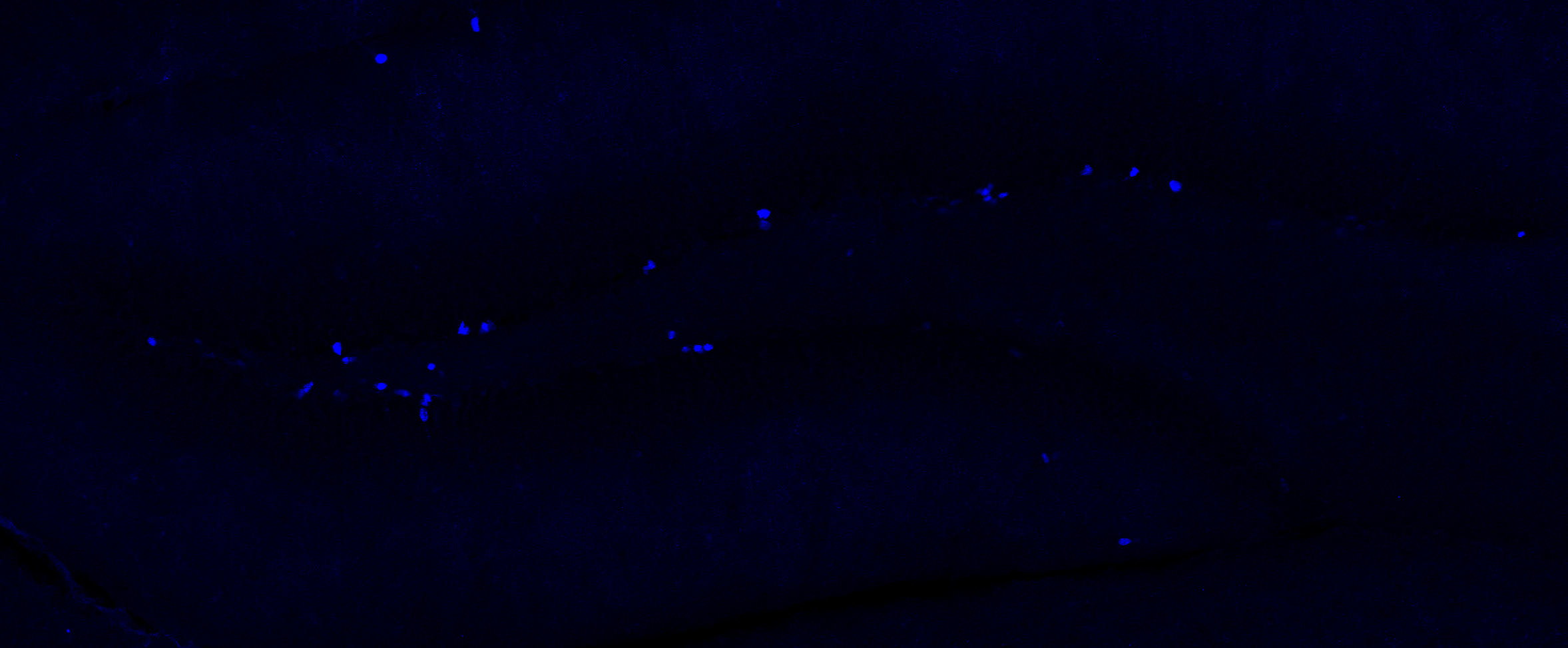

Supplement: Supplementary file 8 — Source data Fig. 3 [file 44318_2025_455_MOESM8_ESM.zip › 3F/Ctrl/original/Ki67.tif]

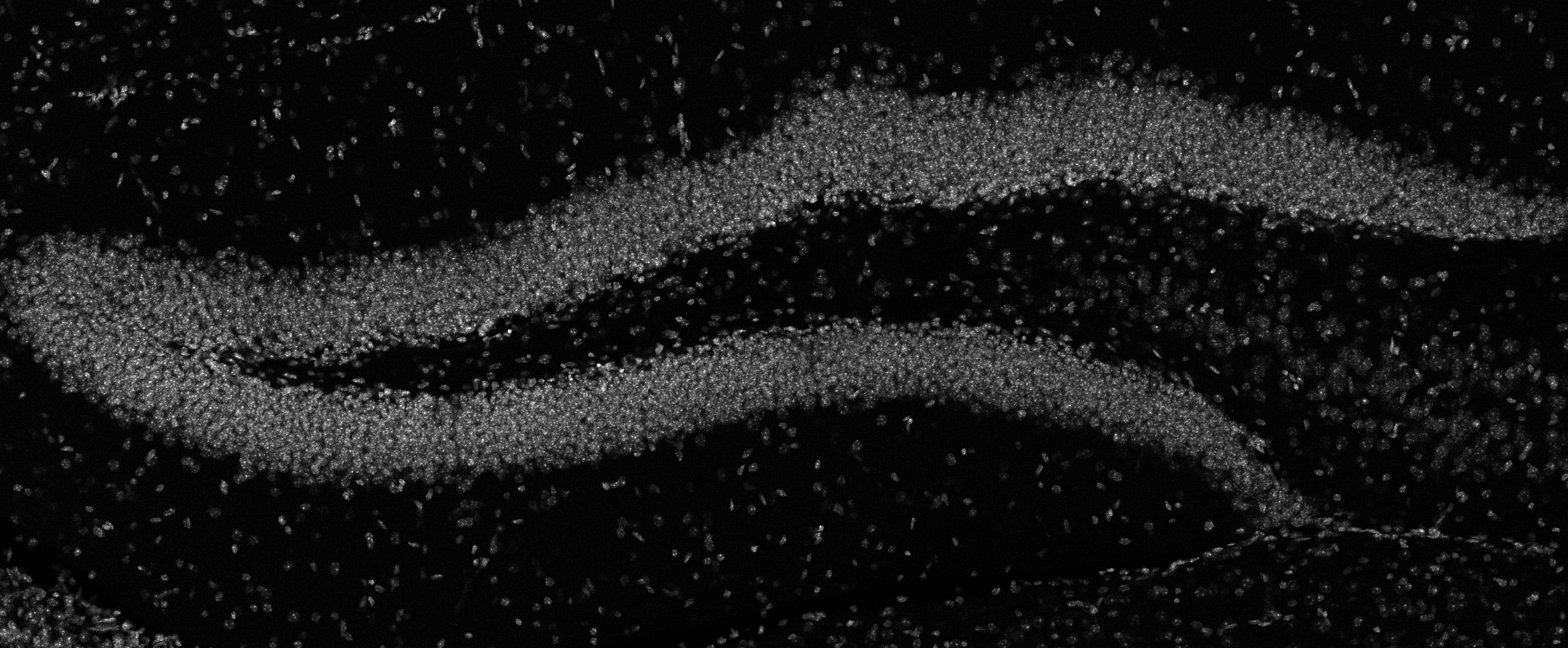

Supplement: Supplementary file 8 — Source data Fig. 3 [file 44318_2025_455_MOESM8_ESM.zip › 3F/Ctrl/original/Hoechst.tif]

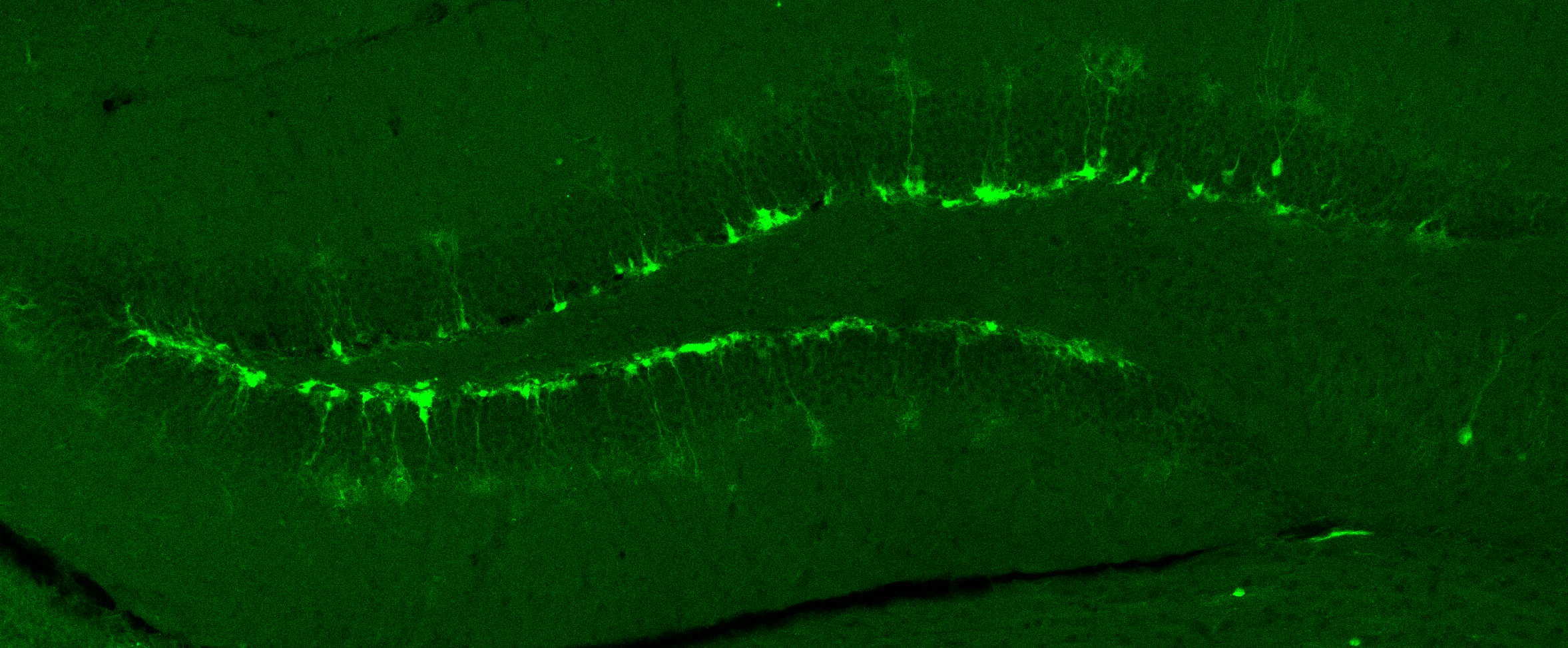

Supplement: Supplementary file 8 — Source data Fig. 3 [file 44318_2025_455_MOESM8_ESM.zip › 3F/Ctrl/original/YFP.tif]

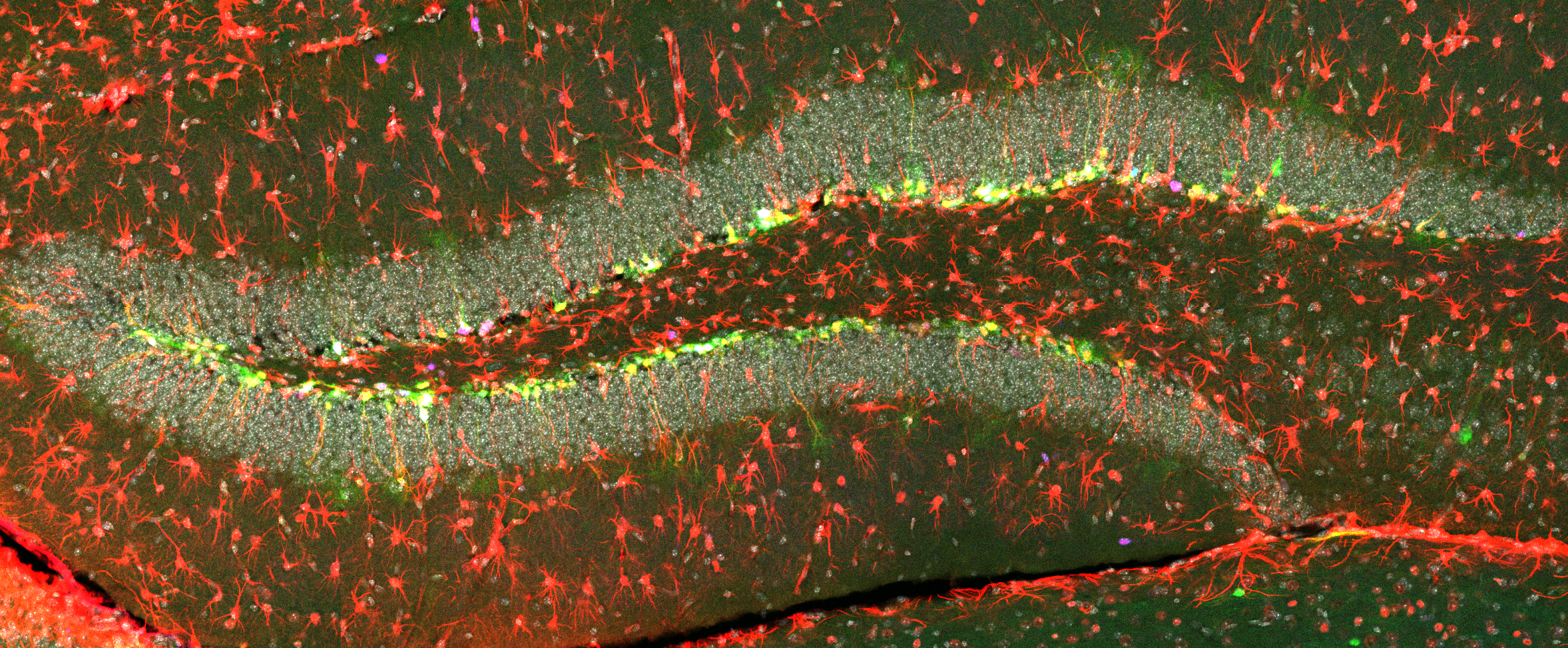

Supplement: Supplementary file 8 — Source data Fig. 3 [file 44318_2025_455_MOESM8_ESM.zip › 3F/Ctrl/original/Merge.tif]

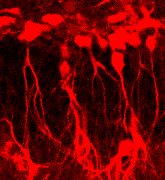

Supplement: Supplementary file 8 — Source data Fig. 3 [file 44318_2025_455_MOESM8_ESM.zip › 3F/Ctrl/edited/Gfap_Sox2.tif]

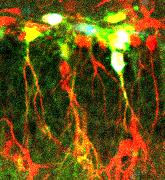

Supplement: Supplementary file 8 — Source data Fig. 3 [file 44318_2025_455_MOESM8_ESM.zip › 3F/Ctrl/edited/Marge.tif]

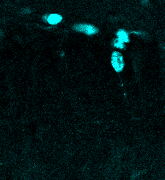

Supplement: Supplementary file 8 — Source data Fig. 3 [file 44318_2025_455_MOESM8_ESM.zip › 3F/Ctrl/edited/Ki67.tif]

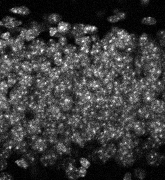

Supplement: Supplementary file 8 — Source data Fig. 3 [file 44318_2025_455_MOESM8_ESM.zip › 3F/Ctrl/edited/Hoechst.tif]

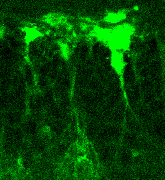

Supplement: Supplementary file 8 — Source data Fig. 3 [file 44318_2025_455_MOESM8_ESM.zip › 3F/Ctrl/edited/YFP.tif]

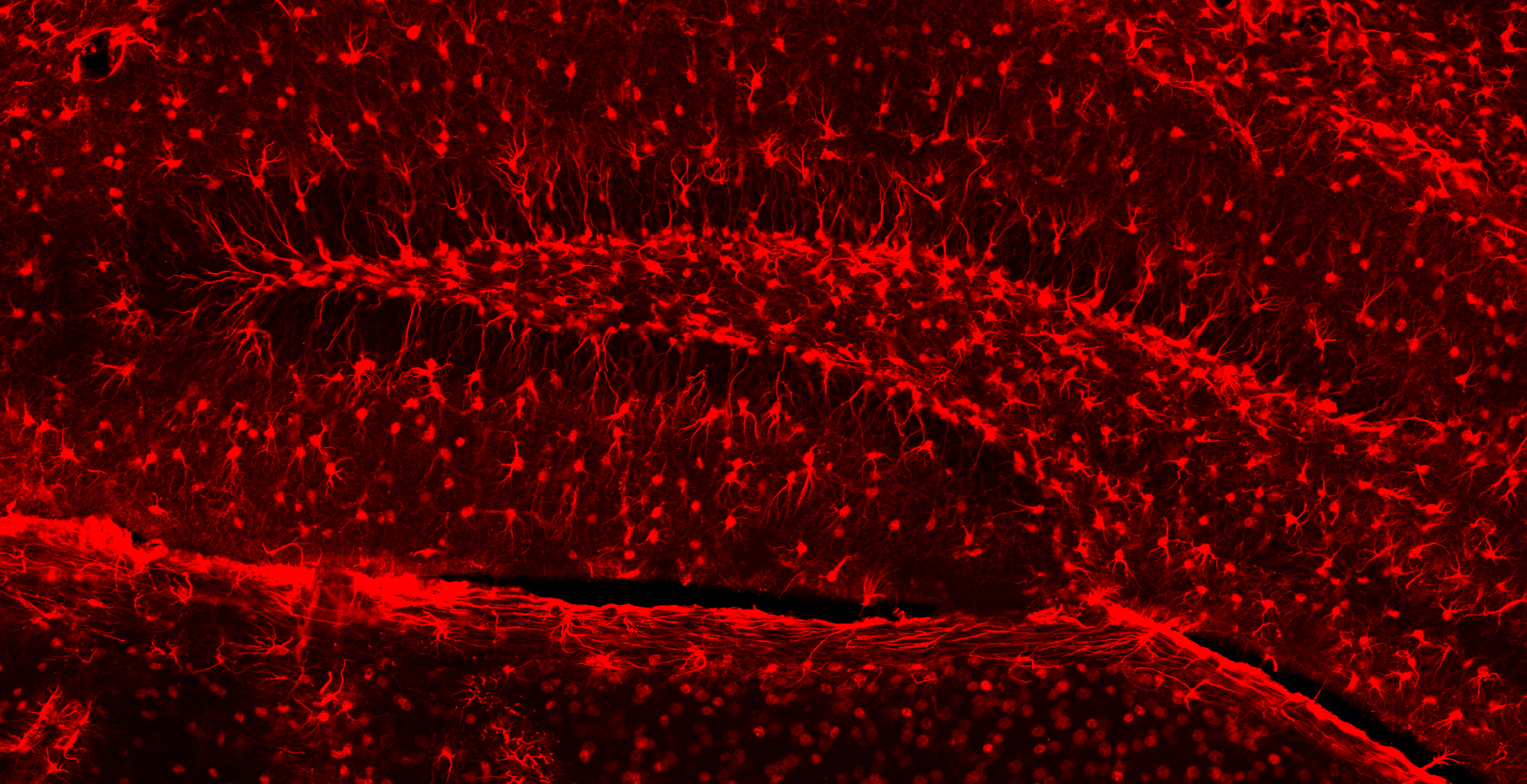

Supplement: Supplementary file 9 — Source data Fig. 4 [file 44318_2025_455_MOESM9_ESM.zip › 4B/original/Gfap_Sox2.tif]

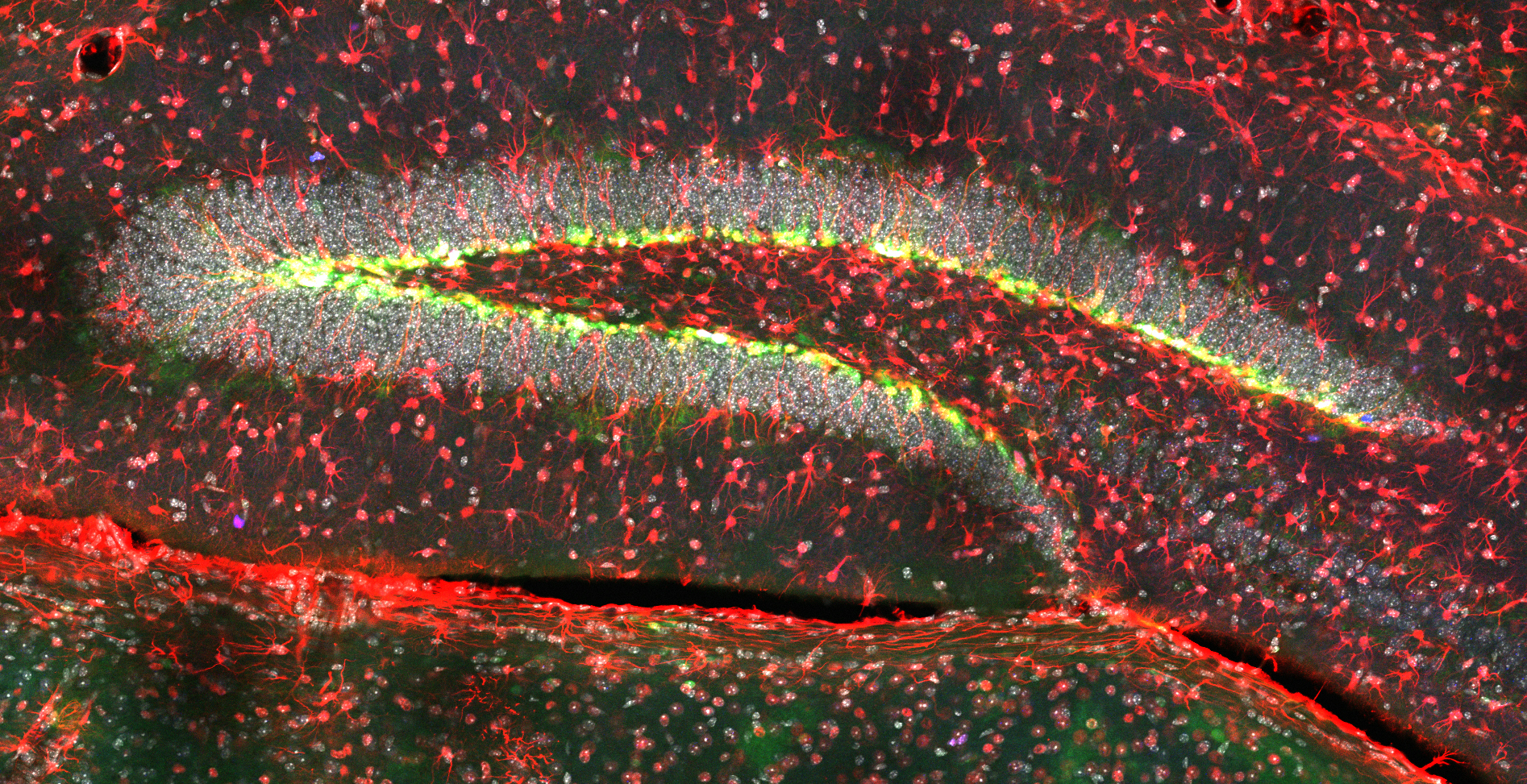

Supplement: Supplementary file 9 — Source data Fig. 4 [file 44318_2025_455_MOESM9_ESM.zip › 4B/original/Marge.tif]

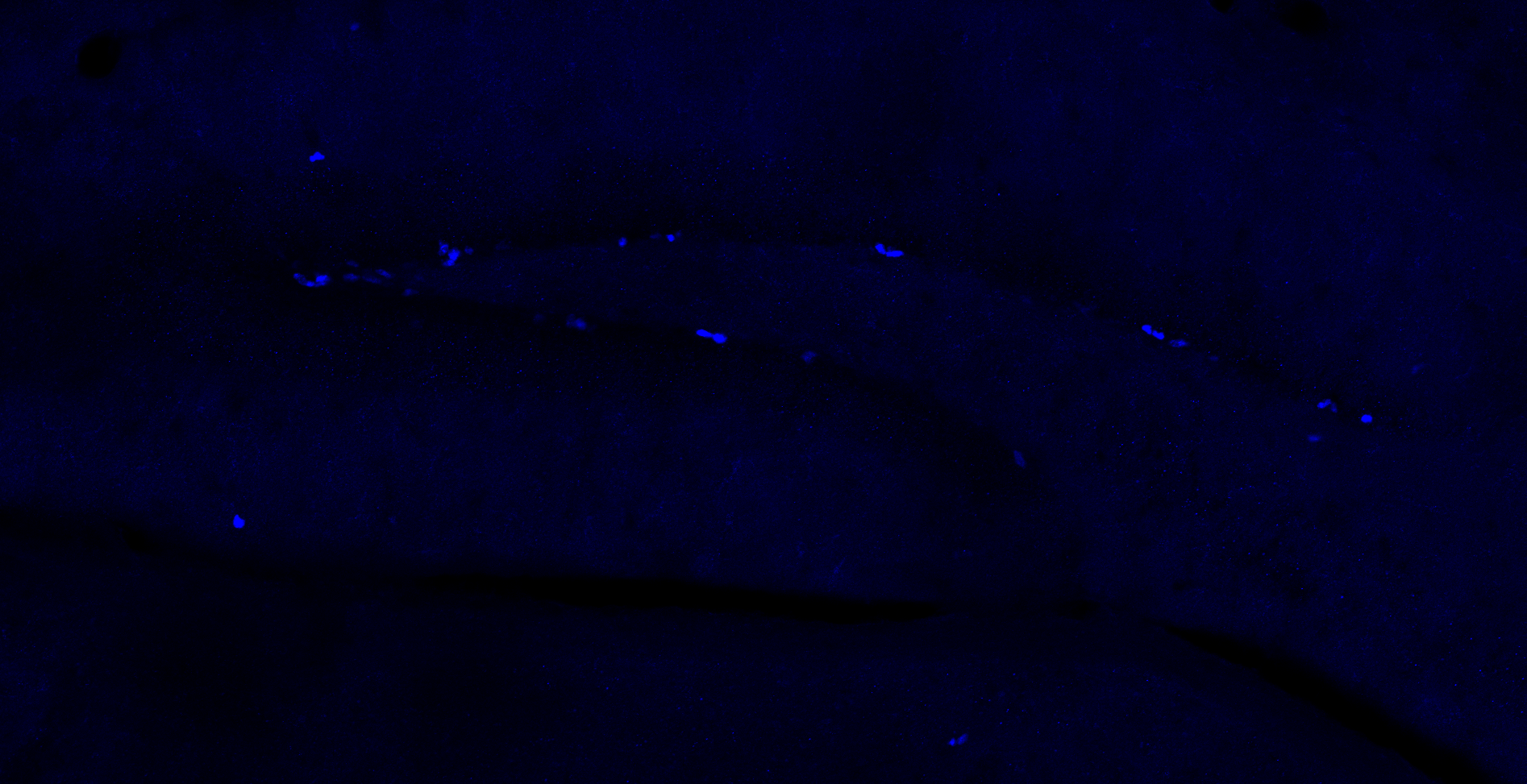

Supplement: Supplementary file 9 — Source data Fig. 4 [file 44318_2025_455_MOESM9_ESM.zip › 4B/original/Ki67.tif]

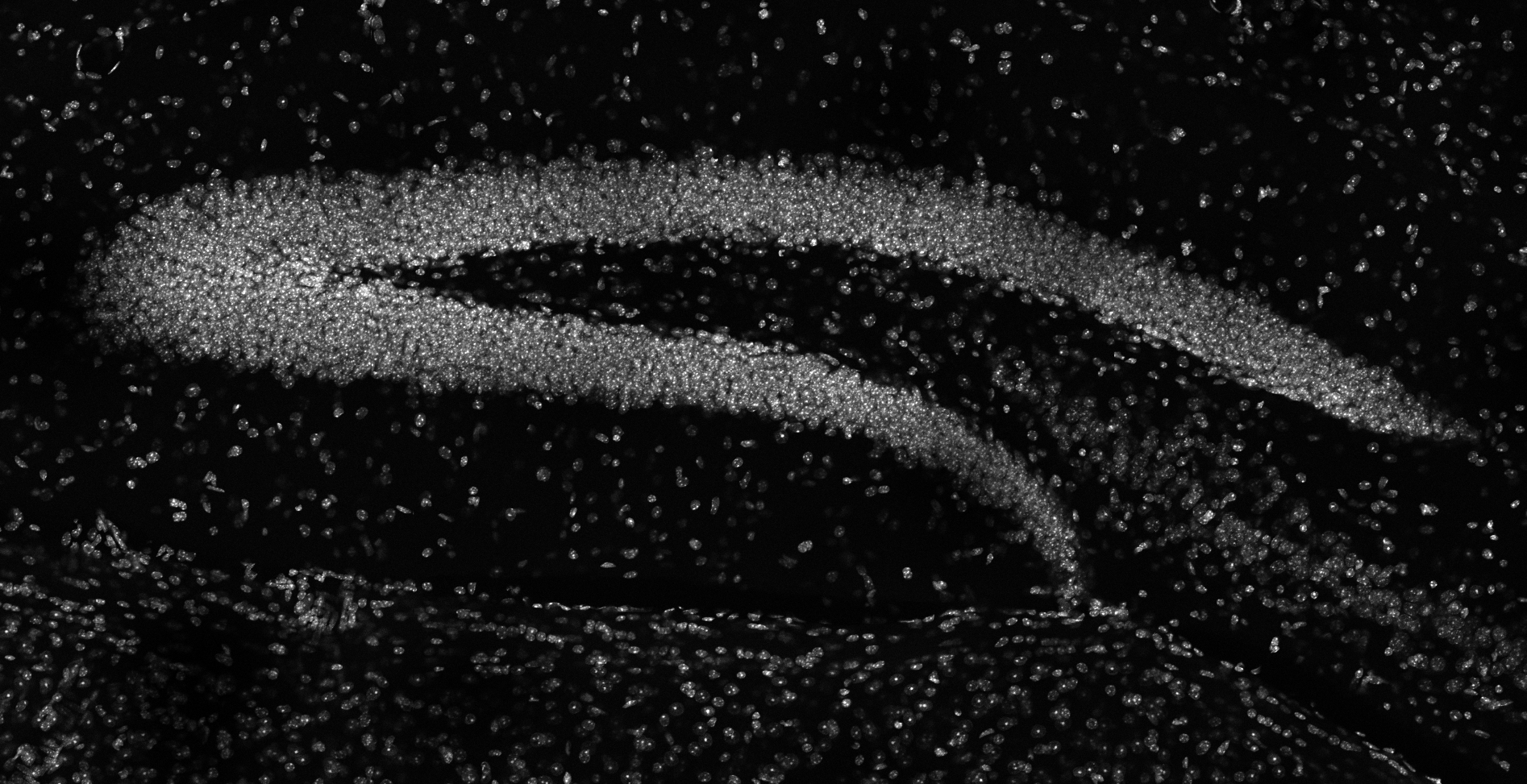

Supplement: Supplementary file 9 — Source data Fig. 4 [file 44318_2025_455_MOESM9_ESM.zip › 4B/original/Hoechst.tif]

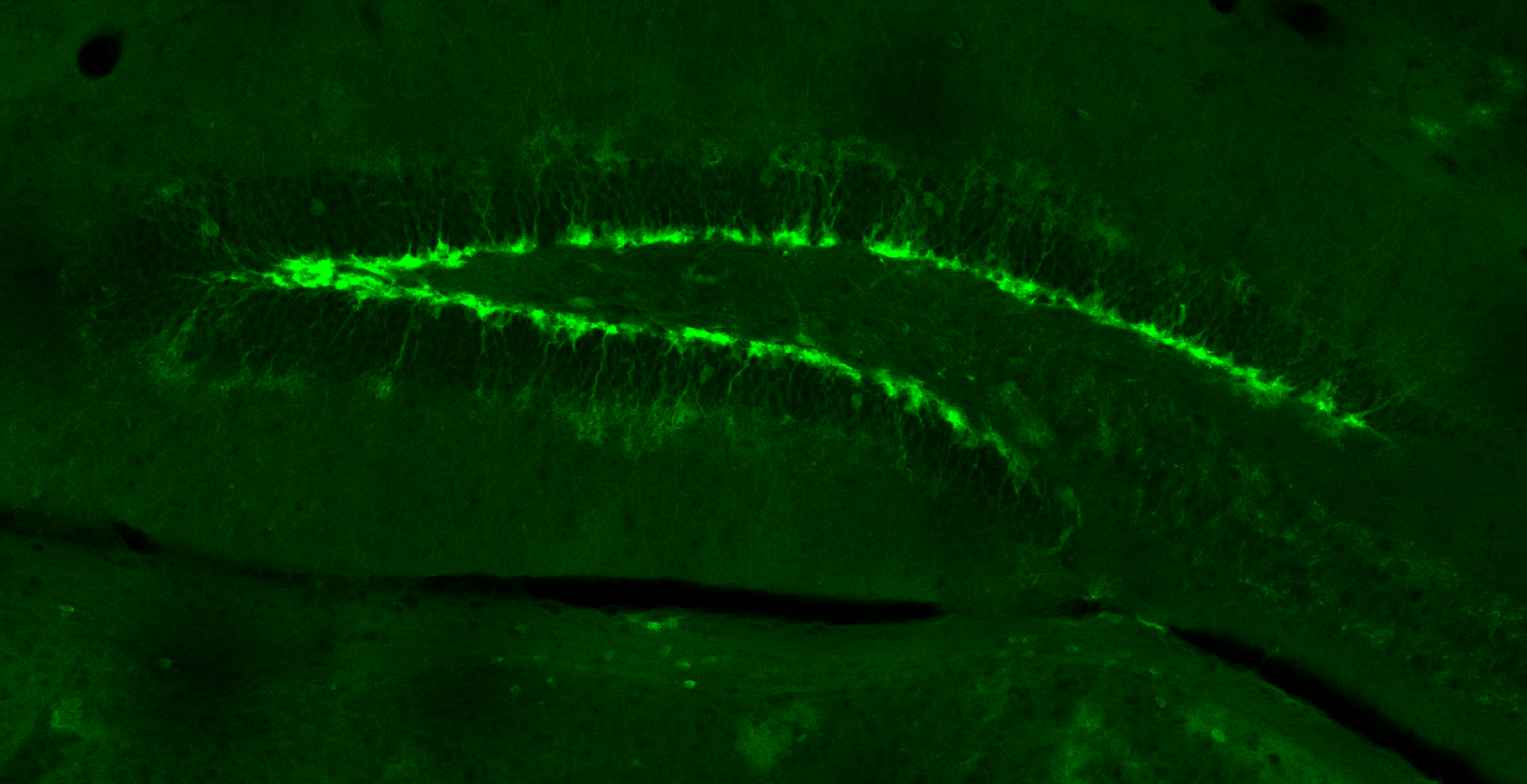

Supplement: Supplementary file 9 — Source data Fig. 4 [file 44318_2025_455_MOESM9_ESM.zip › 4B/original/YFP.tif]

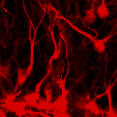

Supplement: Supplementary file 9 — Source data Fig. 4 [file 44318_2025_455_MOESM9_ESM.zip › 4B/edit/Gfap_Sox2.tif]

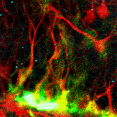

Supplement: Supplementary file 9 — Source data Fig. 4 [file 44318_2025_455_MOESM9_ESM.zip › 4B/edit/Marge.tif]

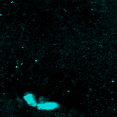

Supplement: Supplementary file 9 — Source data Fig. 4 [file 44318_2025_455_MOESM9_ESM.zip › 4B/edit/Ki67.tif]

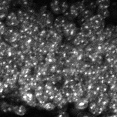

Supplement: Supplementary file 9 — Source data Fig. 4 [file 44318_2025_455_MOESM9_ESM.zip › 4B/edit/Hoechst.tif]

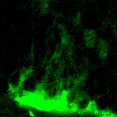

Supplement: Supplementary file 9 — Source data Fig. 4 [file 44318_2025_455_MOESM9_ESM.zip › 4B/edit/YFP.tif]

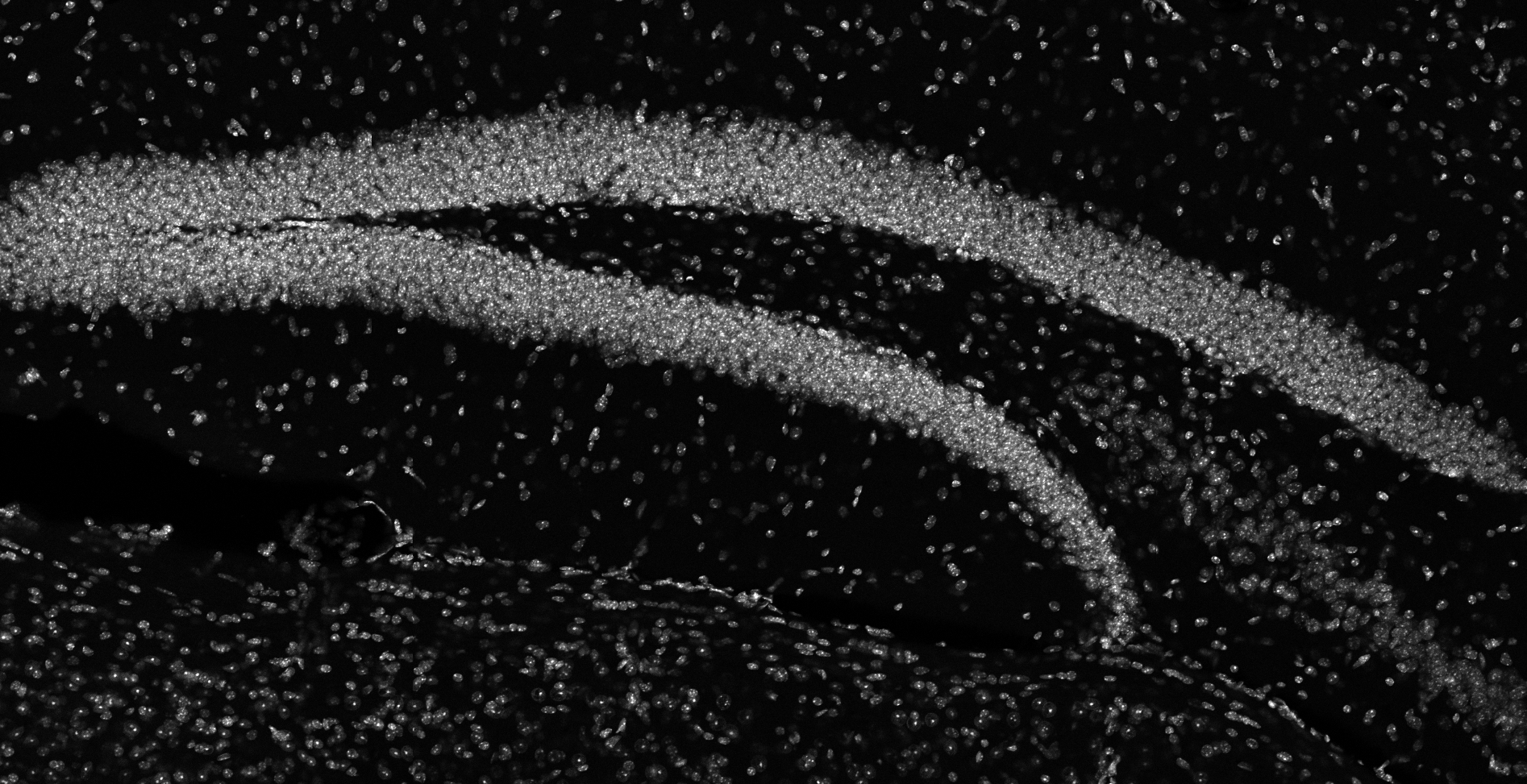

Supplement: Supplementary file 9 — Source data Fig. 4 [file 44318_2025_455_MOESM9_ESM.zip › 4C/original/Hoeches.tif]

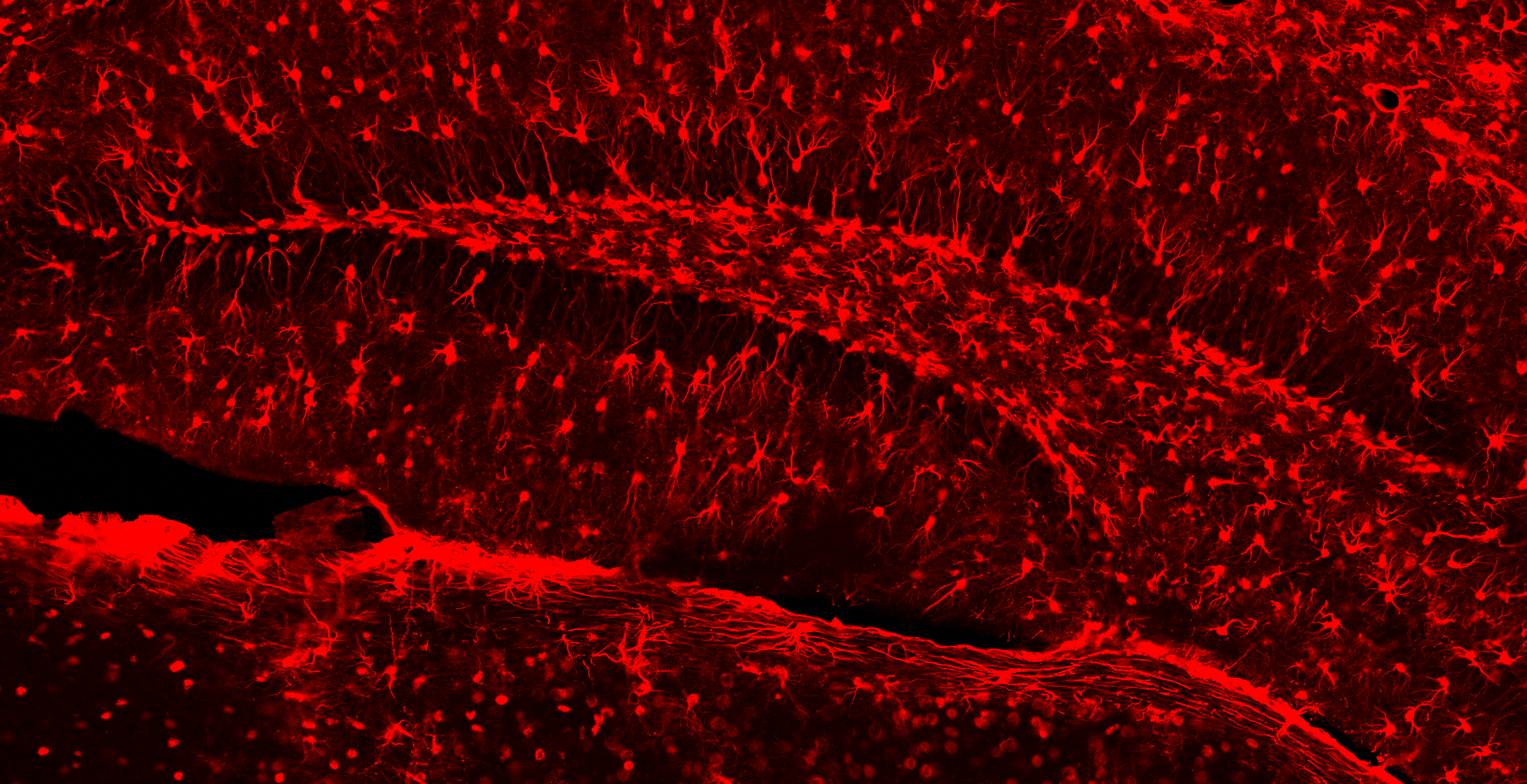

Supplement: Supplementary file 9 — Source data Fig. 4 [file 44318_2025_455_MOESM9_ESM.zip › 4C/original/Gfap_Sox2.tif]

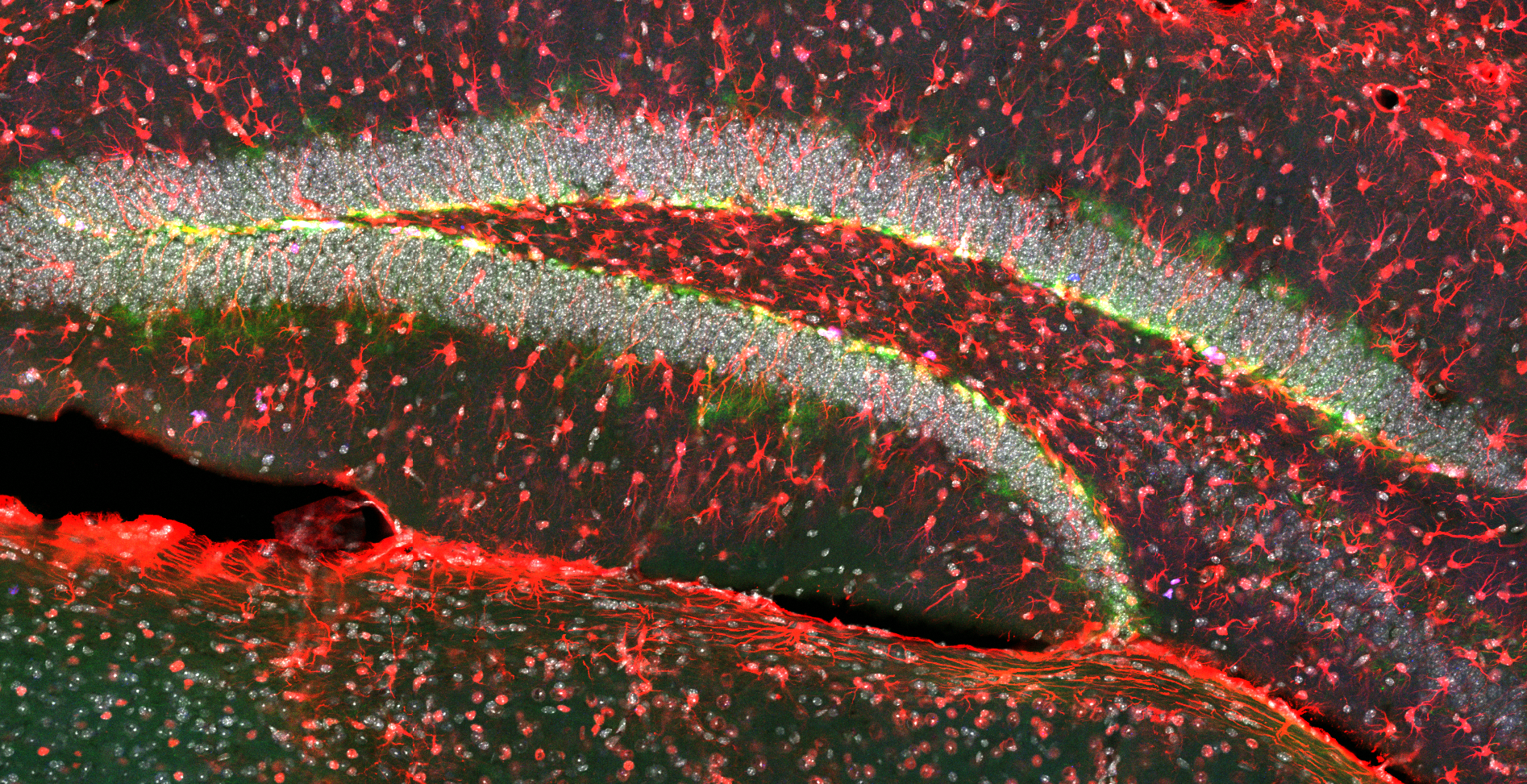

Supplement: Supplementary file 9 — Source data Fig. 4 [file 44318_2025_455_MOESM9_ESM.zip › 4C/original/Marge.tif]

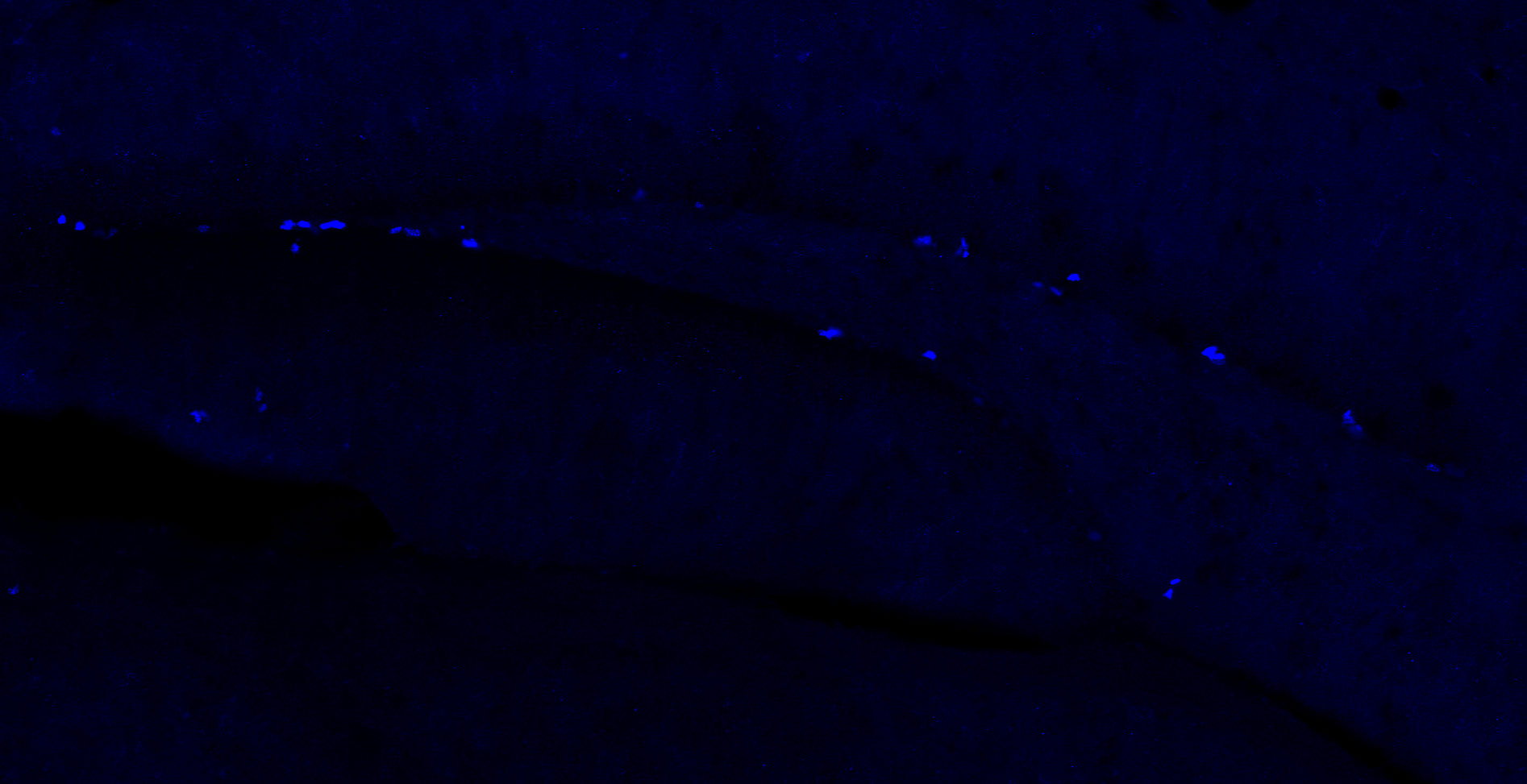

Supplement: Supplementary file 9 — Source data Fig. 4 [file 44318_2025_455_MOESM9_ESM.zip › 4C/original/Ki67.tif]

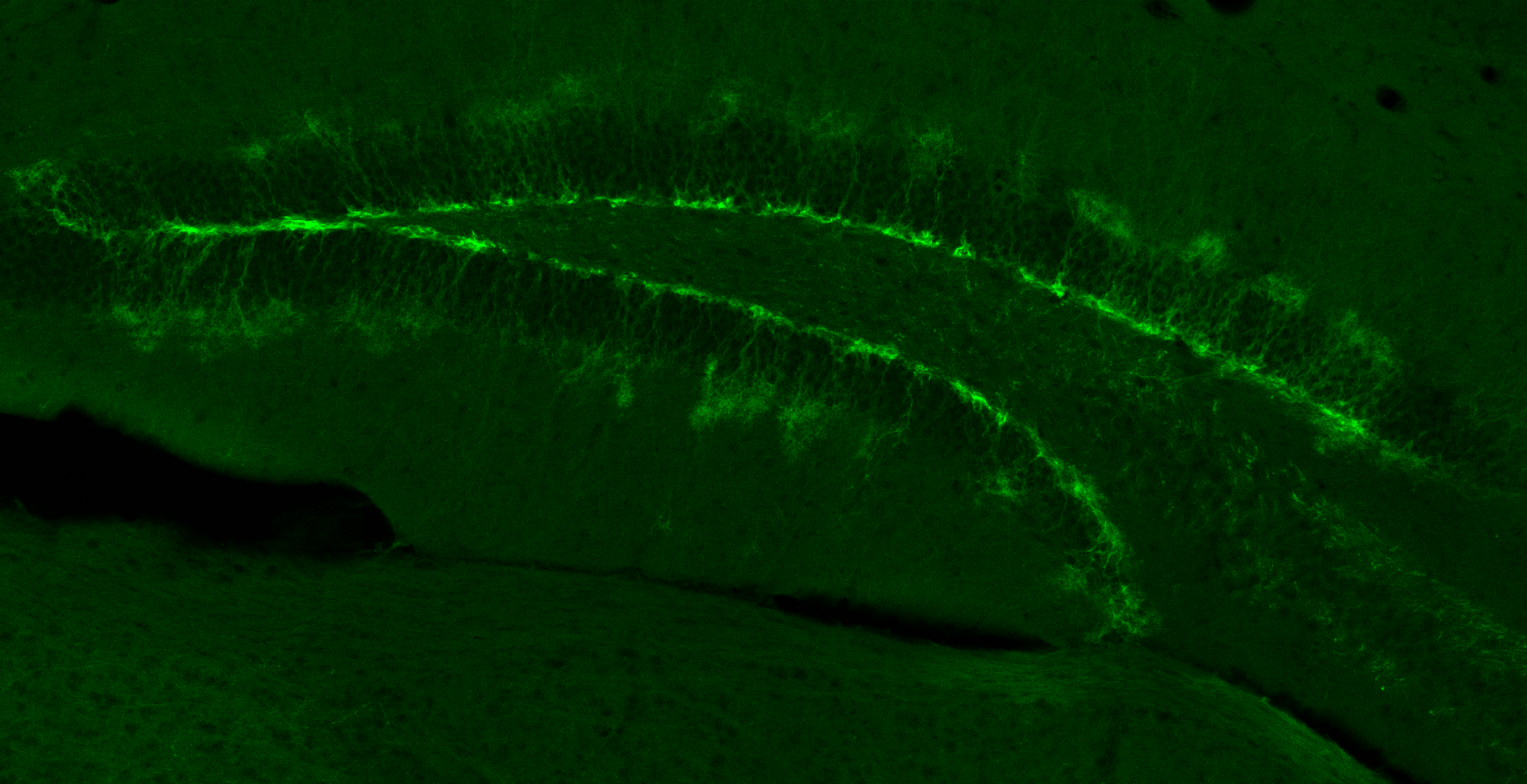

Supplement: Supplementary file 9 — Source data Fig. 4 [file 44318_2025_455_MOESM9_ESM.zip › 4C/original/YFP.tif]

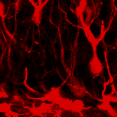

Supplement: Supplementary file 9 — Source data Fig. 4 [file 44318_2025_455_MOESM9_ESM.zip › 4C/edit/Gfap_Sox2.tif]

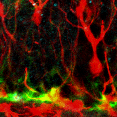

Supplement: Supplementary file 9 — Source data Fig. 4 [file 44318_2025_455_MOESM9_ESM.zip › 4C/edit/Marge.tif]

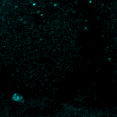

Supplement: Supplementary file 9 — Source data Fig. 4 [file 44318_2025_455_MOESM9_ESM.zip › 4C/edit/Ki67.tif]

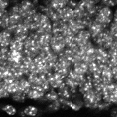

Supplement: Supplementary file 9 — Source data Fig. 4 [file 44318_2025_455_MOESM9_ESM.zip › 4C/edit/Hoechst.tif]

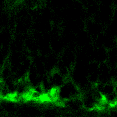

Supplement: Supplementary file 9 — Source data Fig. 4 [file 44318_2025_455_MOESM9_ESM.zip › 4C/edit/YFP.tif]

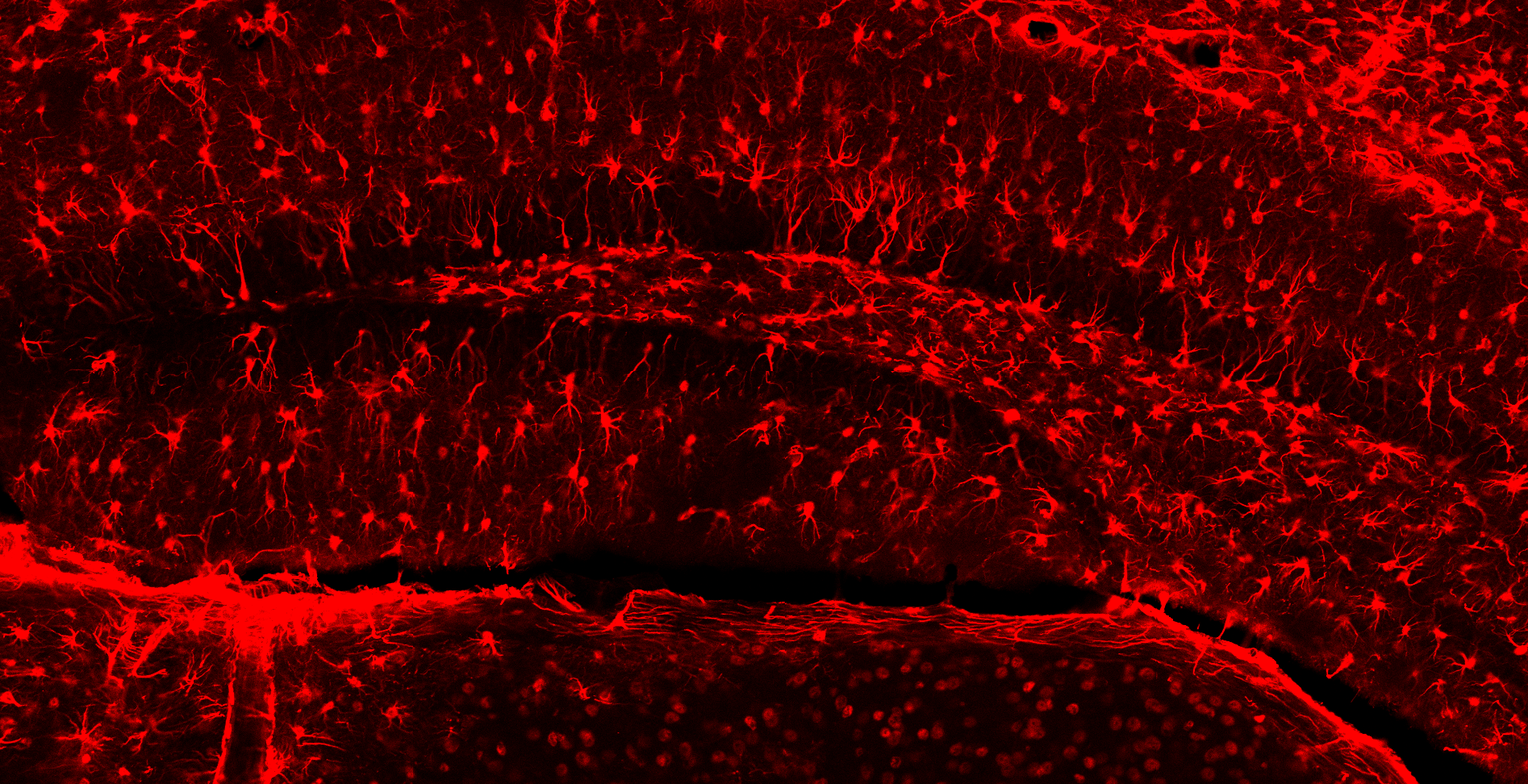

Supplement: Supplementary file 9 — Source data Fig. 4 [file 44318_2025_455_MOESM9_ESM.zip › 4D/original/Gfap_Sox2.tif]

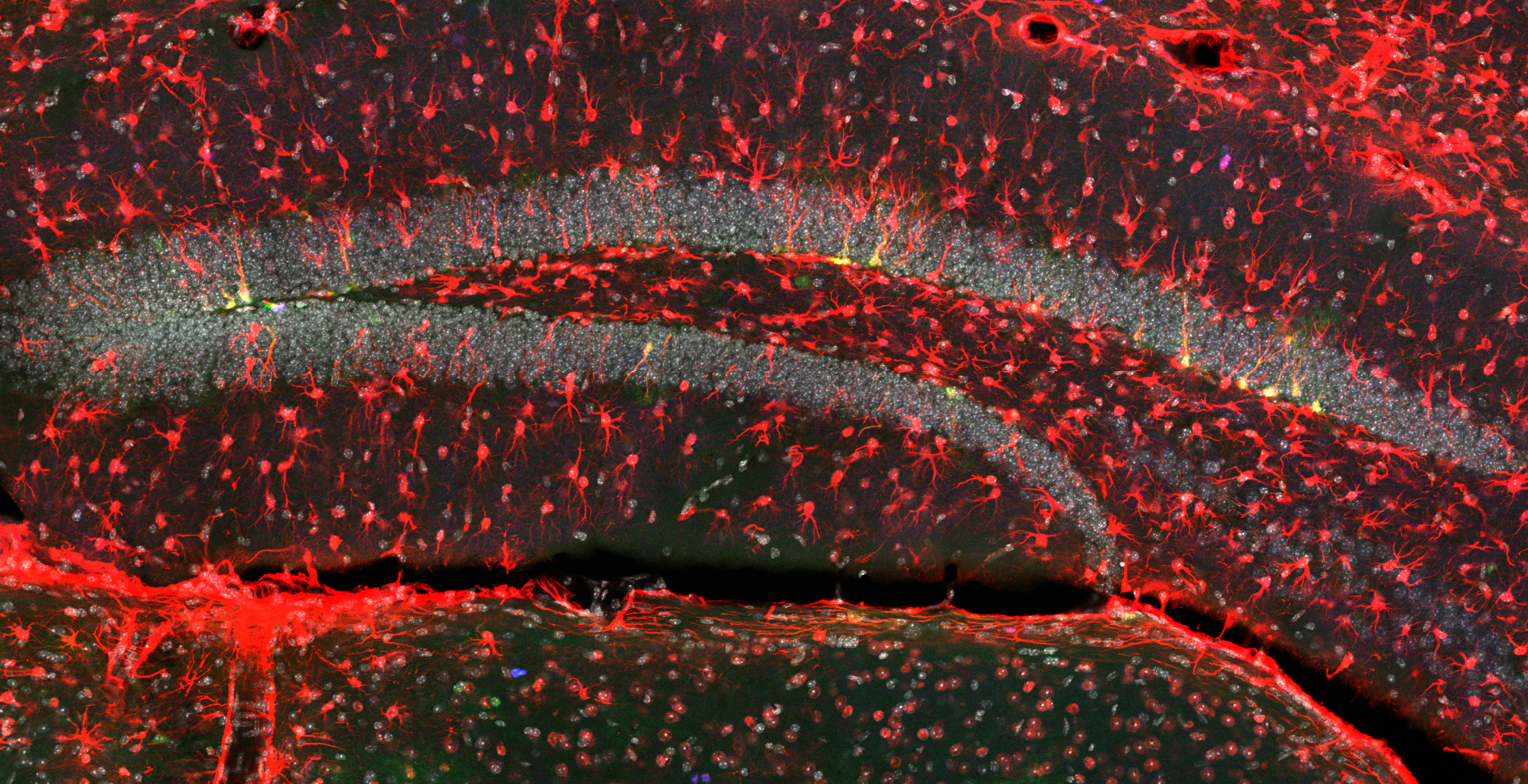

Supplement: Supplementary file 9 — Source data Fig. 4 [file 44318_2025_455_MOESM9_ESM.zip › 4D/original/Marge.tif]

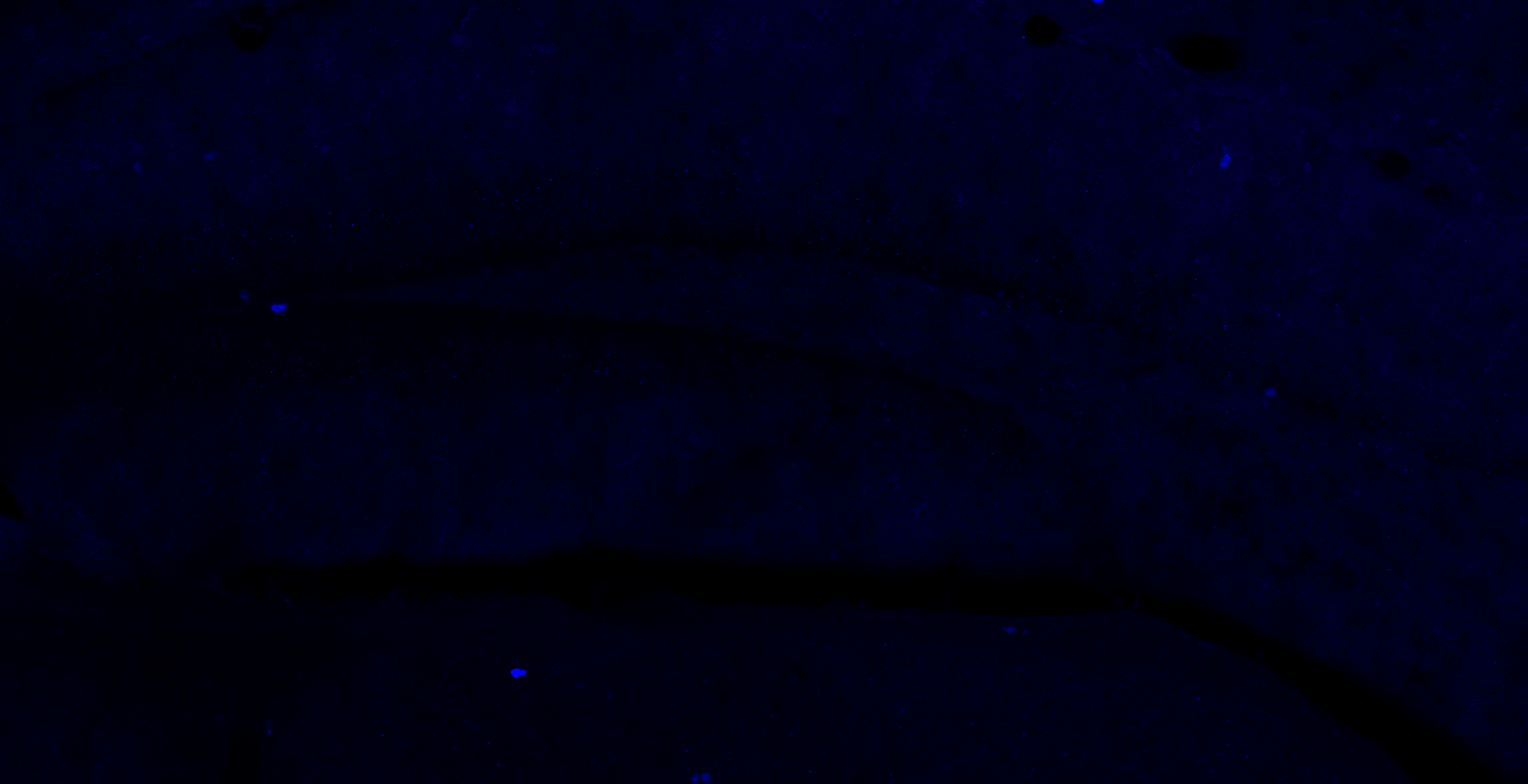

Supplement: Supplementary file 9 — Source data Fig. 4 [file 44318_2025_455_MOESM9_ESM.zip › 4D/original/Ki67.tif]

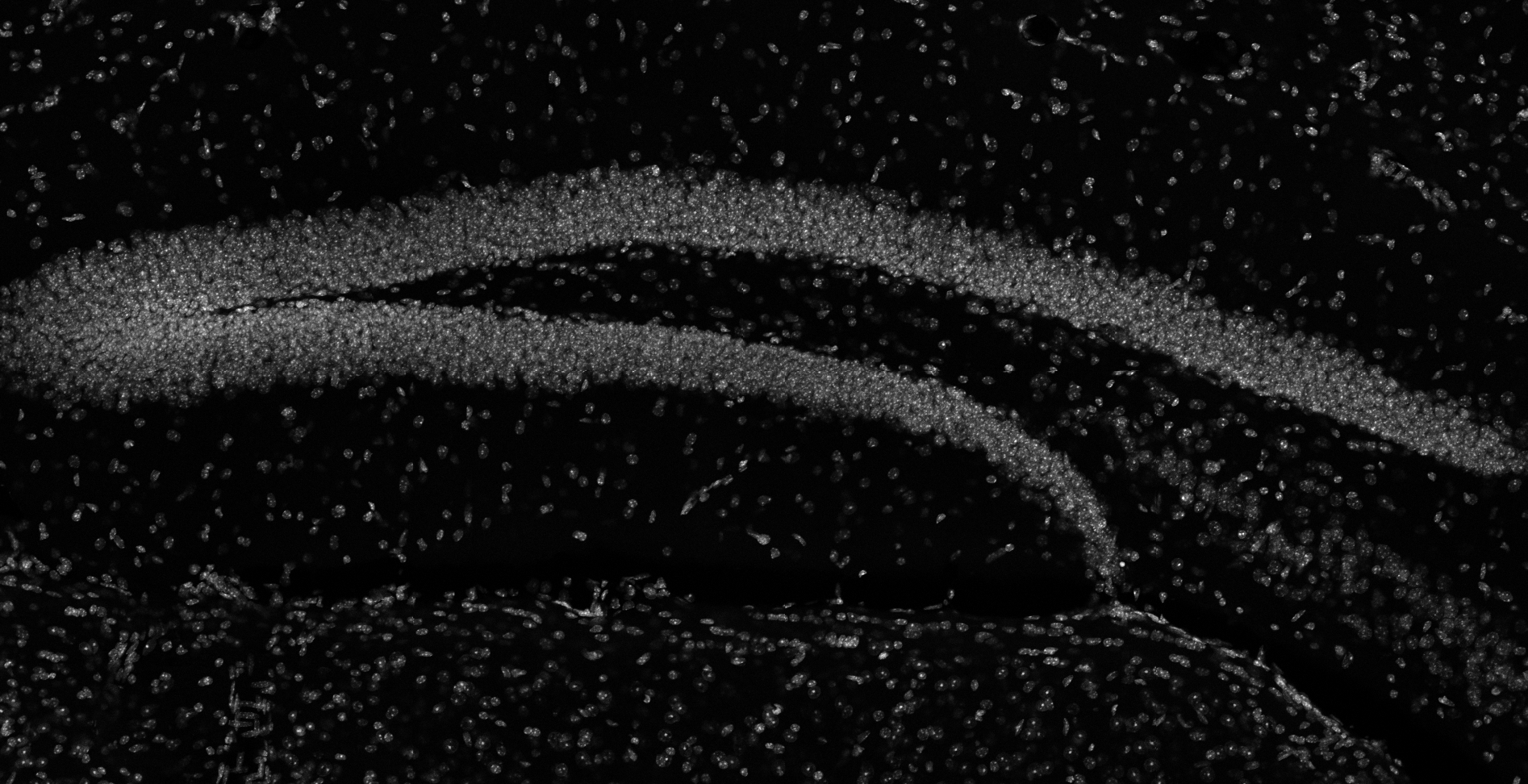

Supplement: Supplementary file 9 — Source data Fig. 4 [file 44318_2025_455_MOESM9_ESM.zip › 4D/original/Hoechst.tif]

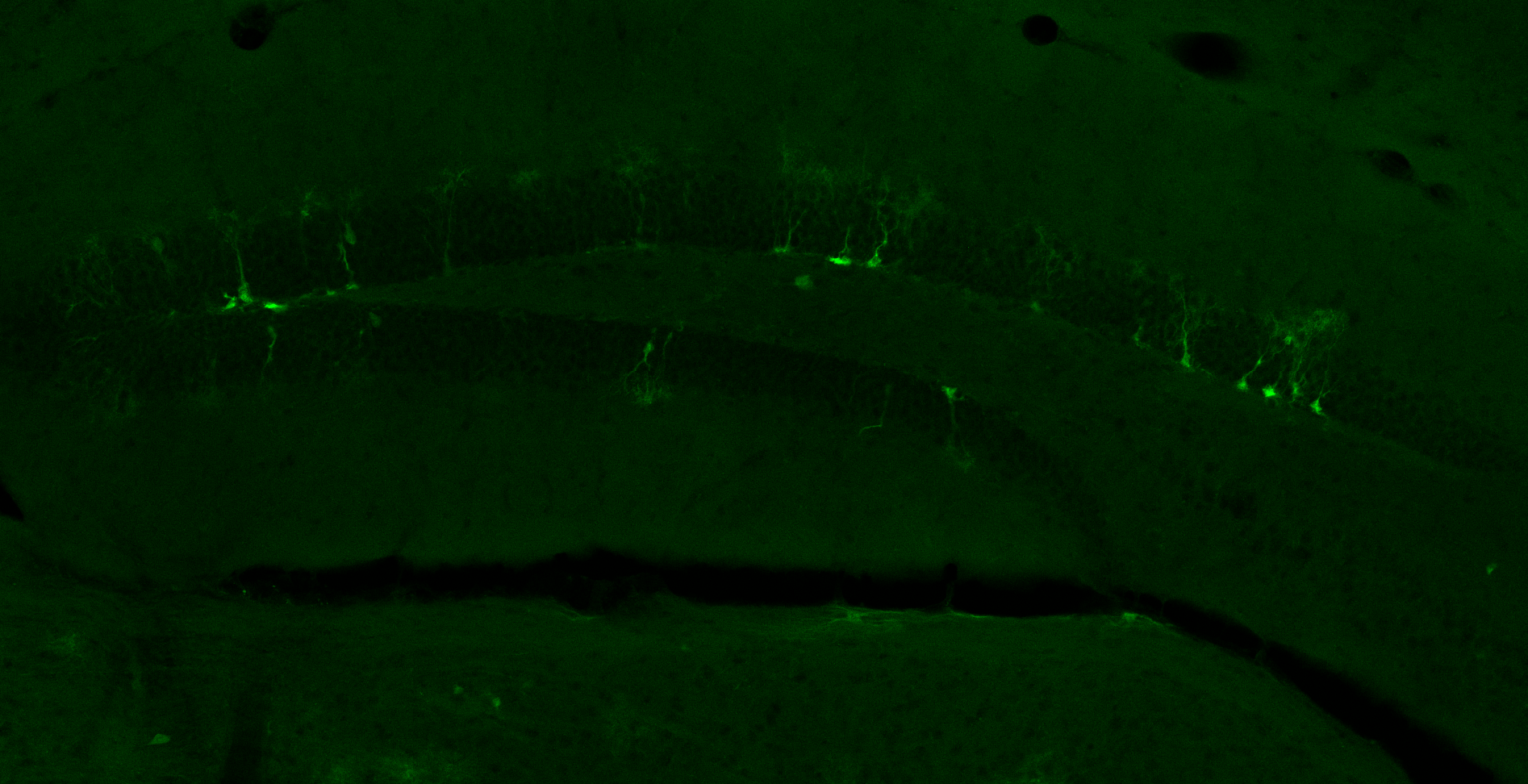

Supplement: Supplementary file 9 — Source data Fig. 4 [file 44318_2025_455_MOESM9_ESM.zip › 4D/original/YFP.tif]

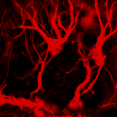

Supplement: Supplementary file 9 — Source data Fig. 4 [file 44318_2025_455_MOESM9_ESM.zip › 4D/edit/Gfap_Sox2.tif]

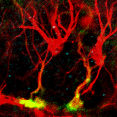

Supplement: Supplementary file 9 — Source data Fig. 4 [file 44318_2025_455_MOESM9_ESM.zip › 4D/edit/Marge.tif]

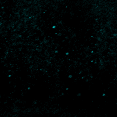

Supplement: Supplementary file 9 — Source data Fig. 4 [file 44318_2025_455_MOESM9_ESM.zip › 4D/edit/Ki67.tif]

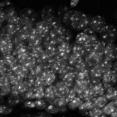

Supplement: Supplementary file 9 — Source data Fig. 4 [file 44318_2025_455_MOESM9_ESM.zip › 4D/edit/Hoechst.tif]

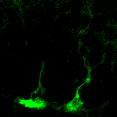

Supplement: Supplementary file 9 — Source data Fig. 4 [file 44318_2025_455_MOESM9_ESM.zip › 4D/edit/YFP.tif]

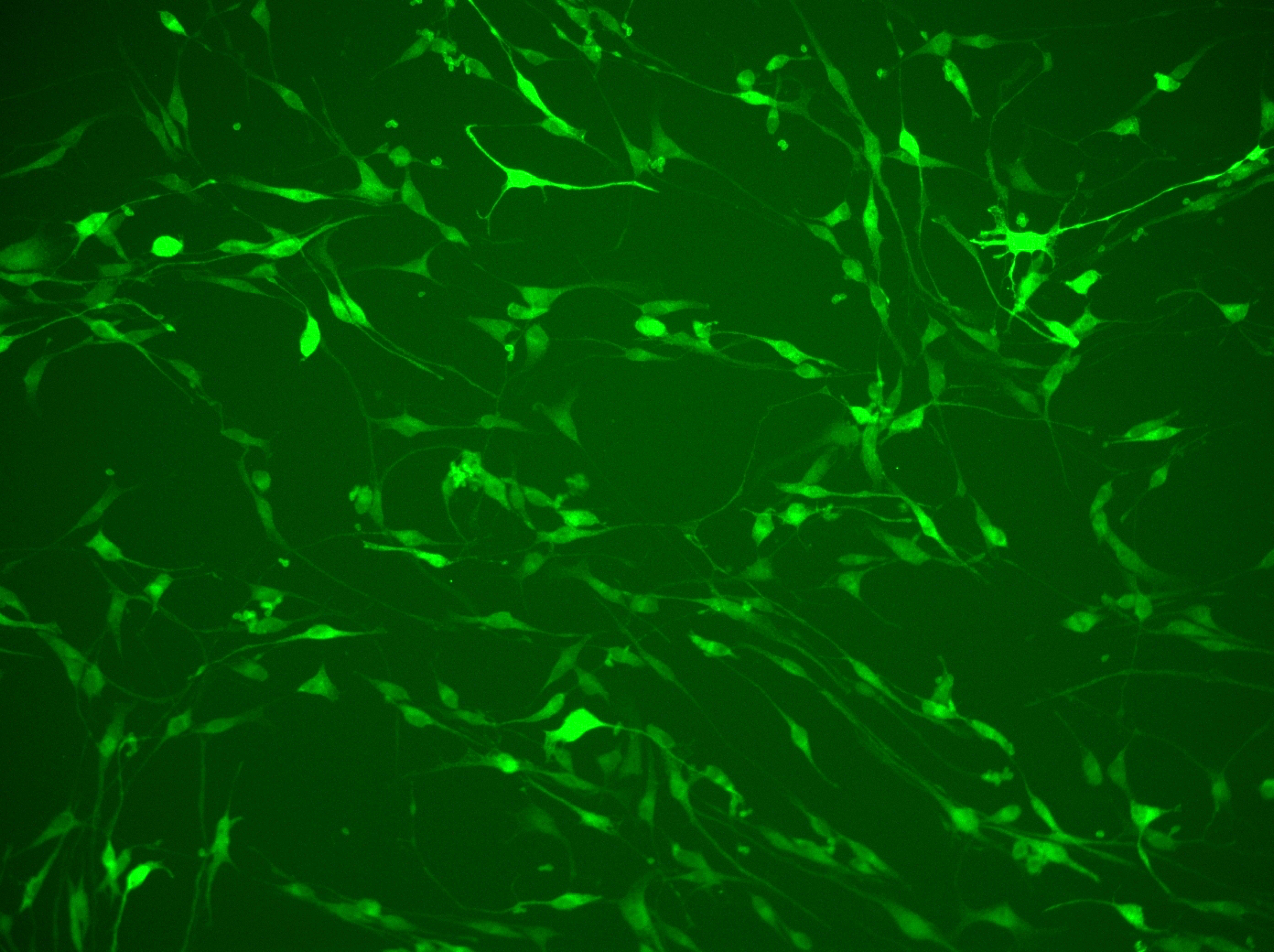

Supplement: Supplementary file 11 — Appendix Figure Source Data sd_S4_5 [file 44318_2025_455_MOESM11_ESM.zip › E/Ctrl/S4_E_Ctrl_1.tiff]

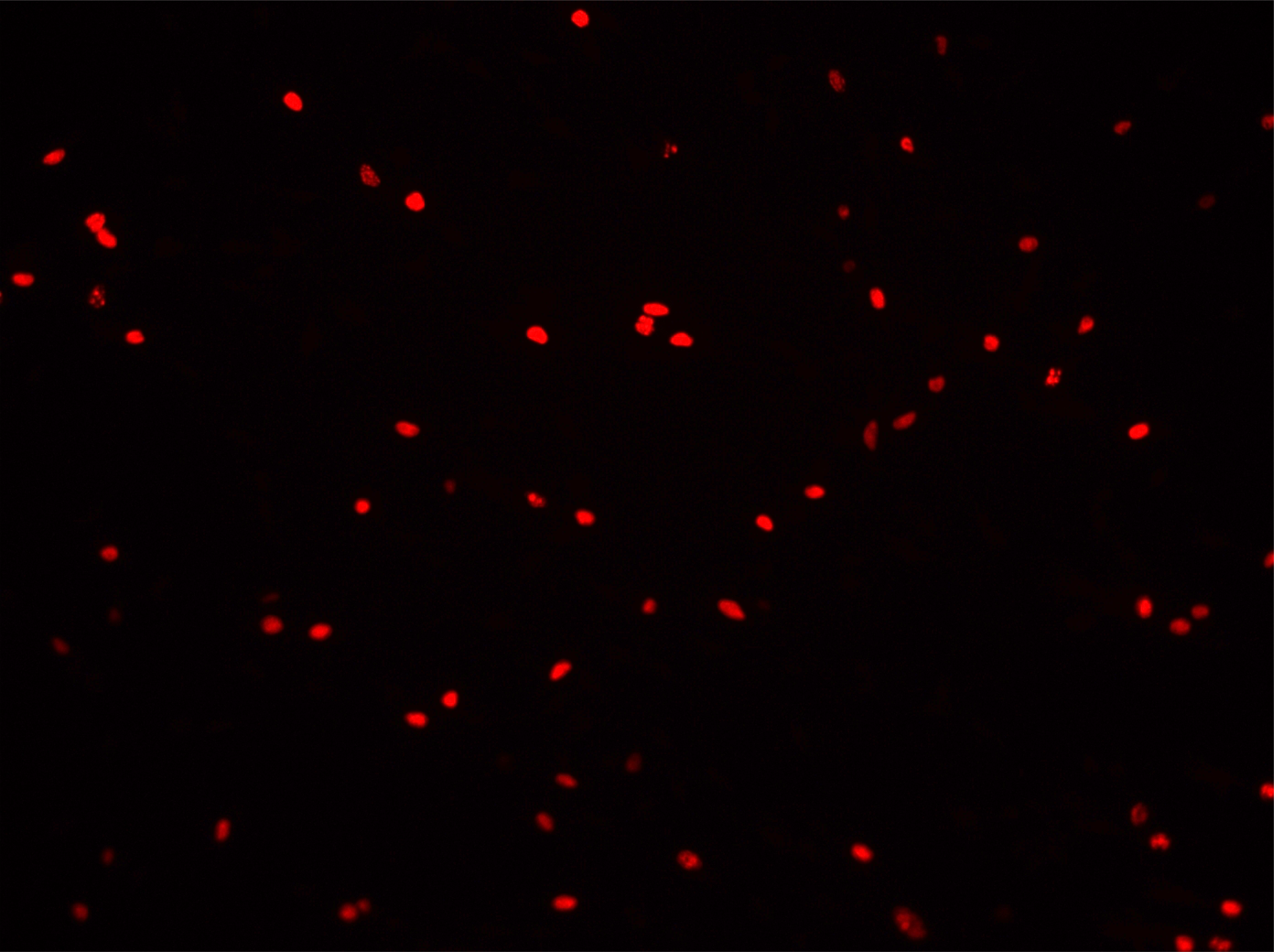

Supplement: Supplementary file 11 — Appendix Figure Source Data sd_S4_5 [file 44318_2025_455_MOESM11_ESM.zip › E/Ctrl/S4_E_Ctrl_2.tiff]

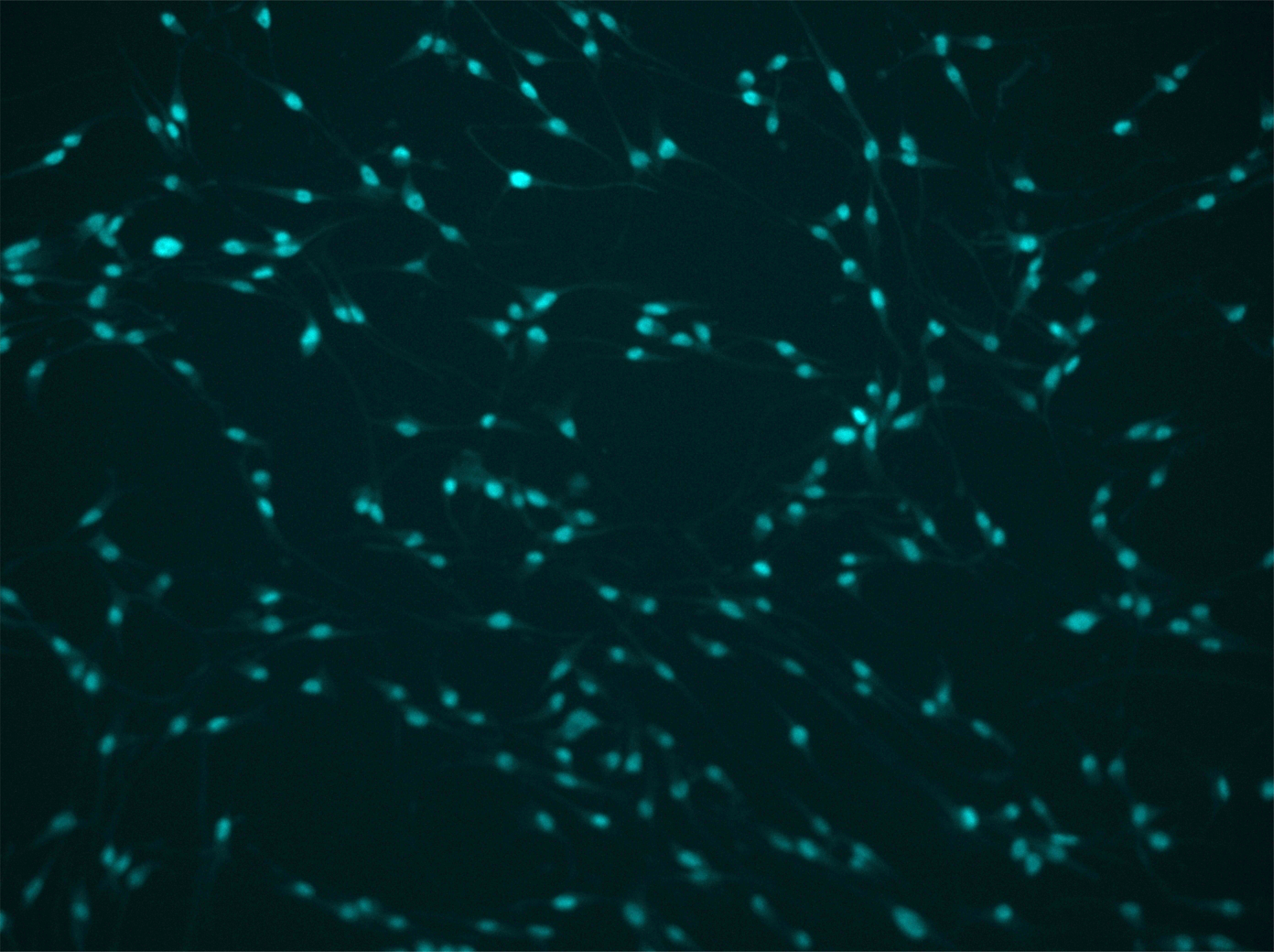

Supplement: Supplementary file 11 — Appendix Figure Source Data sd_S4_5 [file 44318_2025_455_MOESM11_ESM.zip › E/Ctrl/S4_E_Ctrl_3.tiff]

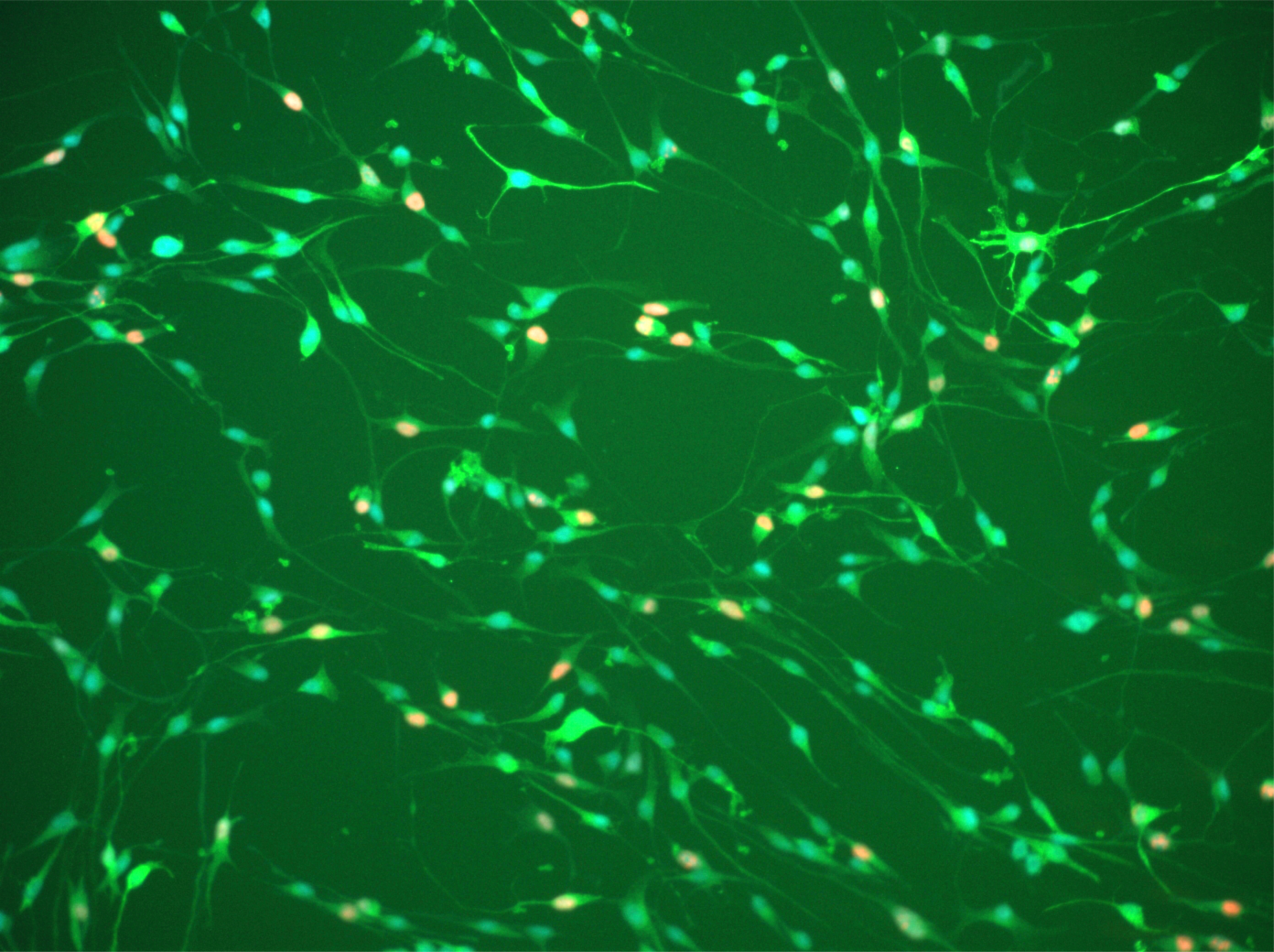

Supplement: Supplementary file 11 — Appendix Figure Source Data sd_S4_5 [file 44318_2025_455_MOESM11_ESM.zip › E/Ctrl/S4_E_Ctrl_4.tiff]

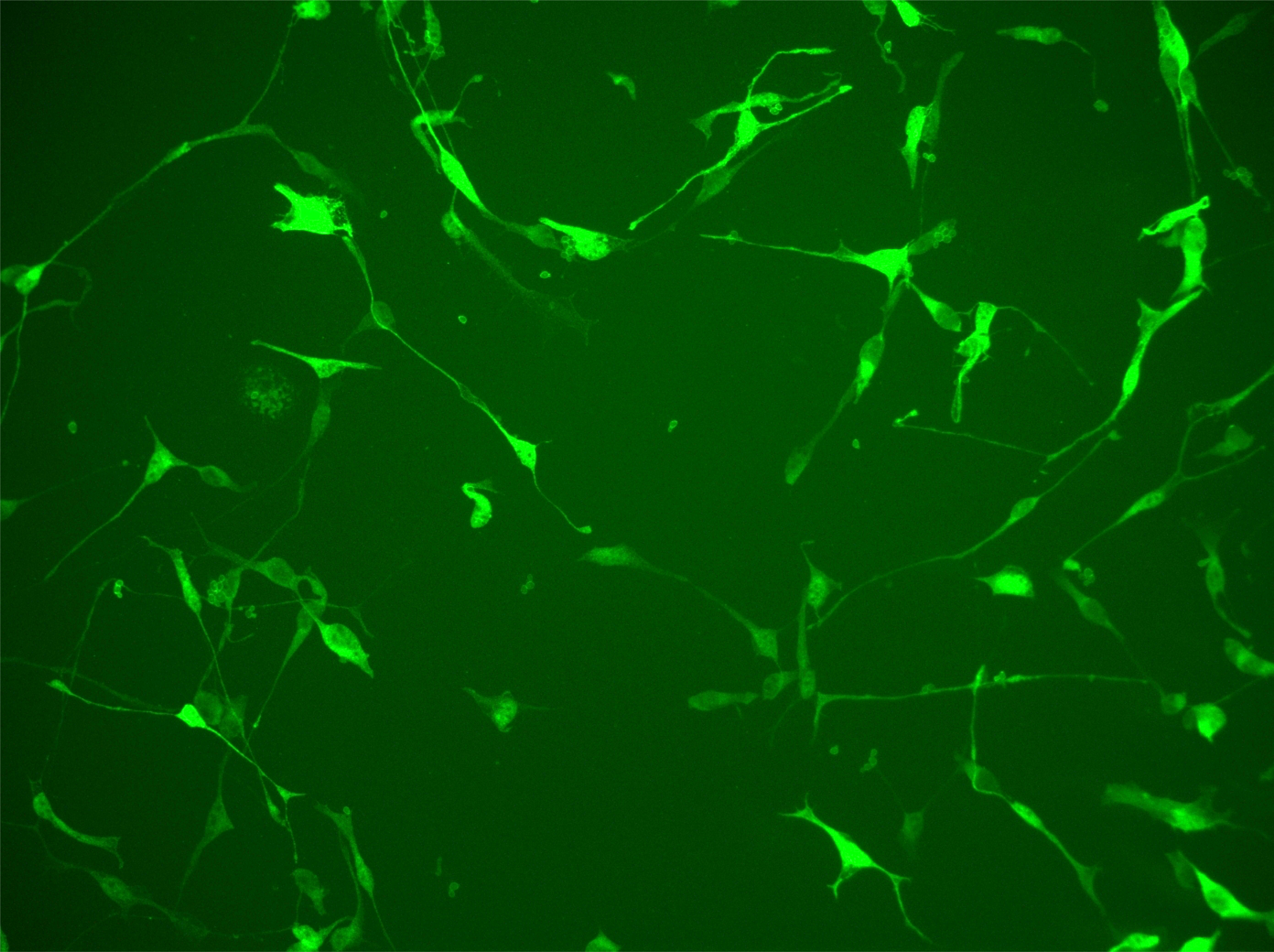

Supplement: Supplementary file 11 — Appendix Figure Source Data sd_S4_5 [file 44318_2025_455_MOESM11_ESM.zip › E/KD/S4_E_KD_1.tiff]

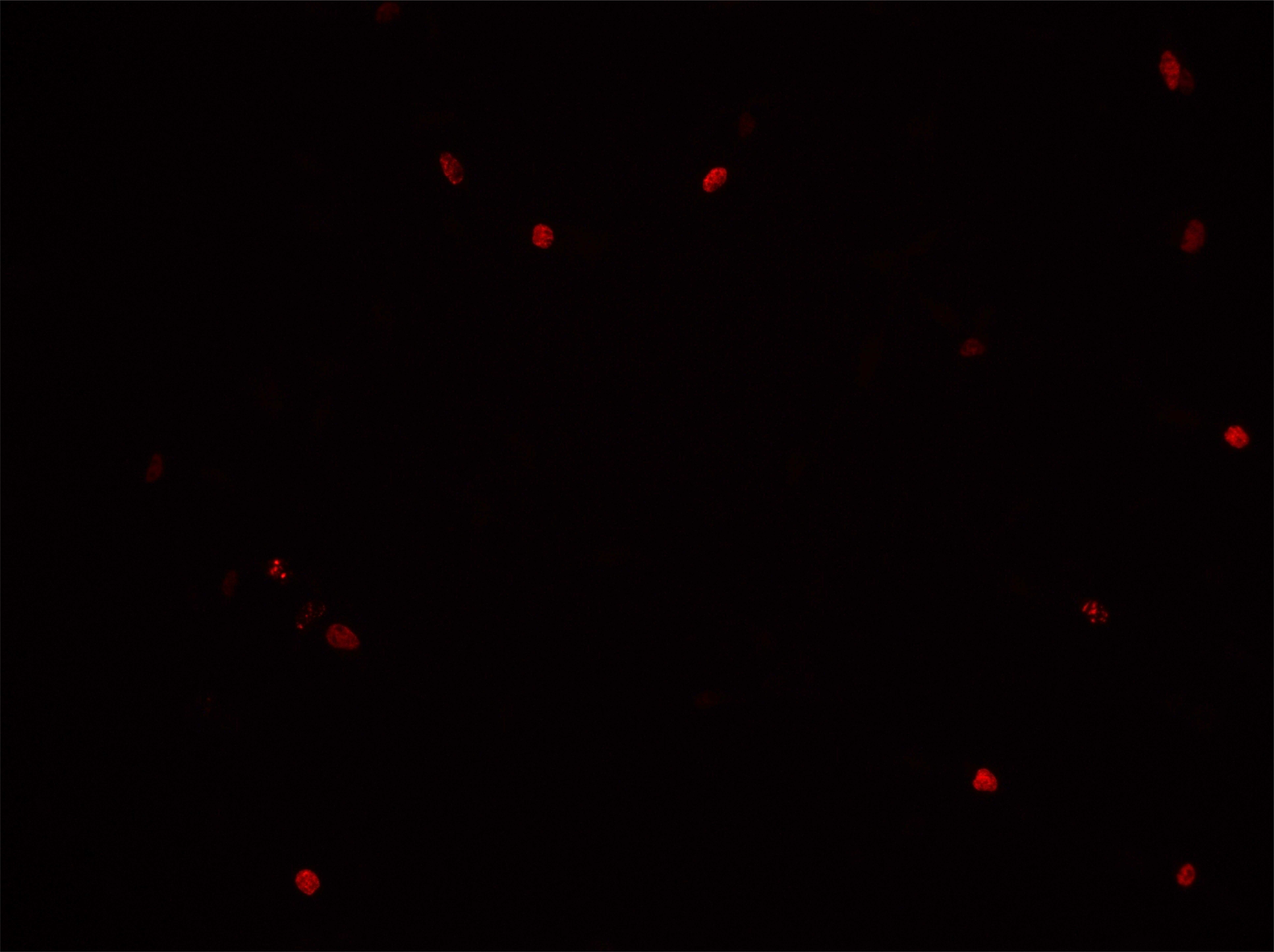

Supplement: Supplementary file 11 — Appendix Figure Source Data sd_S4_5 [file 44318_2025_455_MOESM11_ESM.zip › E/KD/S4_E_KD_2.tiff]

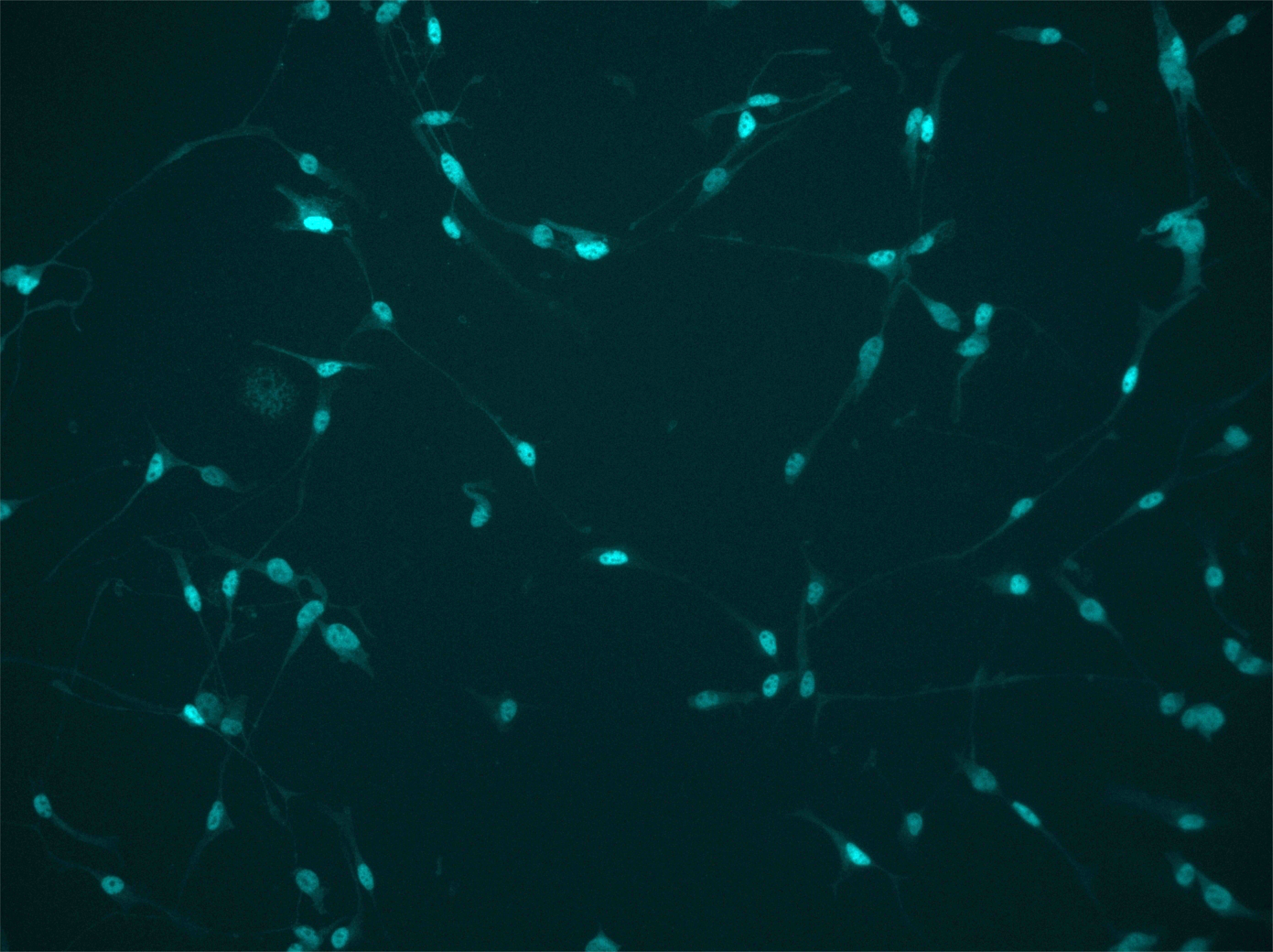

Supplement: Supplementary file 11 — Appendix Figure Source Data sd_S4_5 [file 44318_2025_455_MOESM11_ESM.zip › E/KD/S4_E_KD_3.tiff]

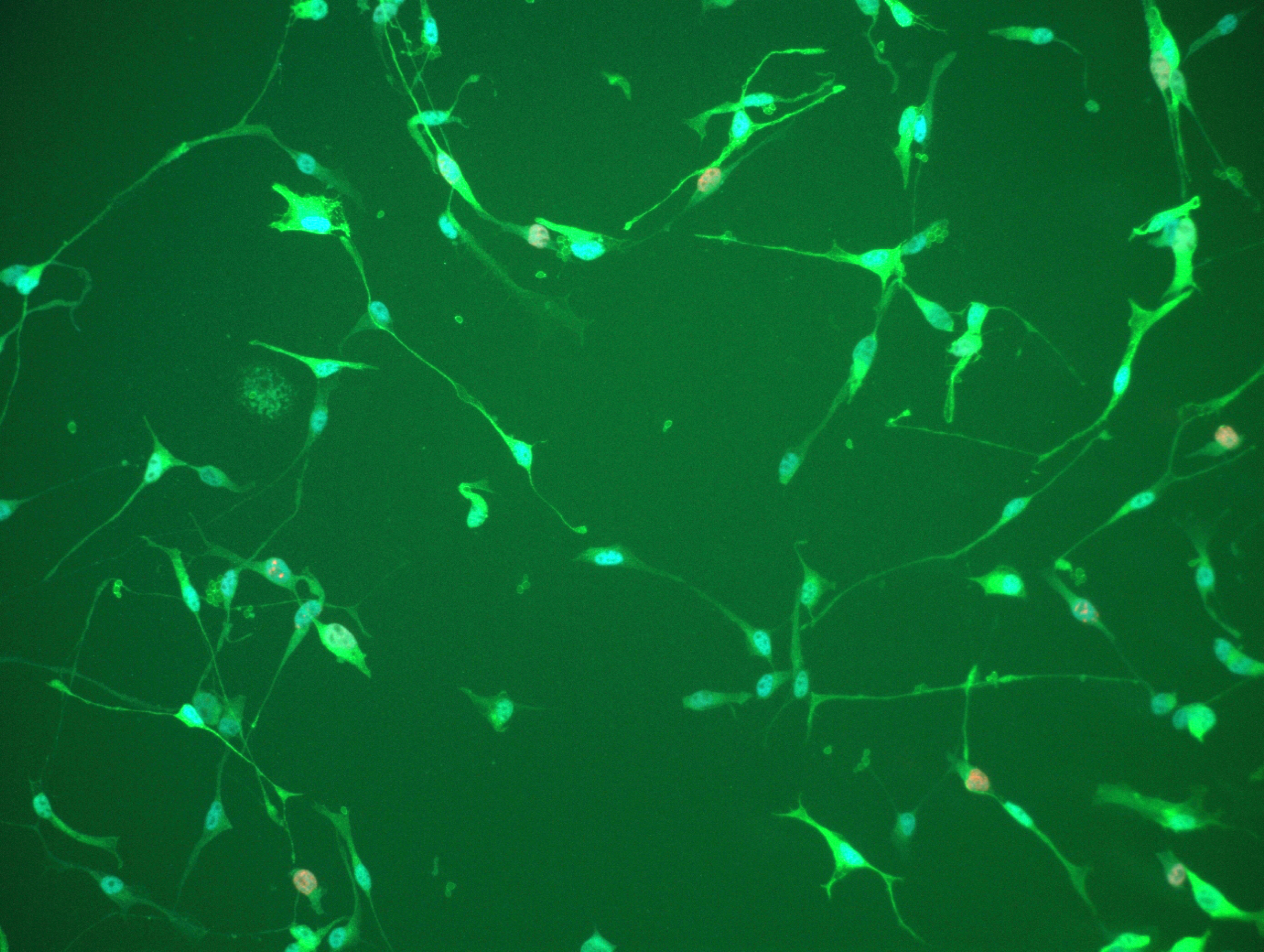

Supplement: Supplementary file 11 — Appendix Figure Source Data sd_S4_5 [file 44318_2025_455_MOESM11_ESM.zip › E/KD/S4_E_KD_4.tiff]

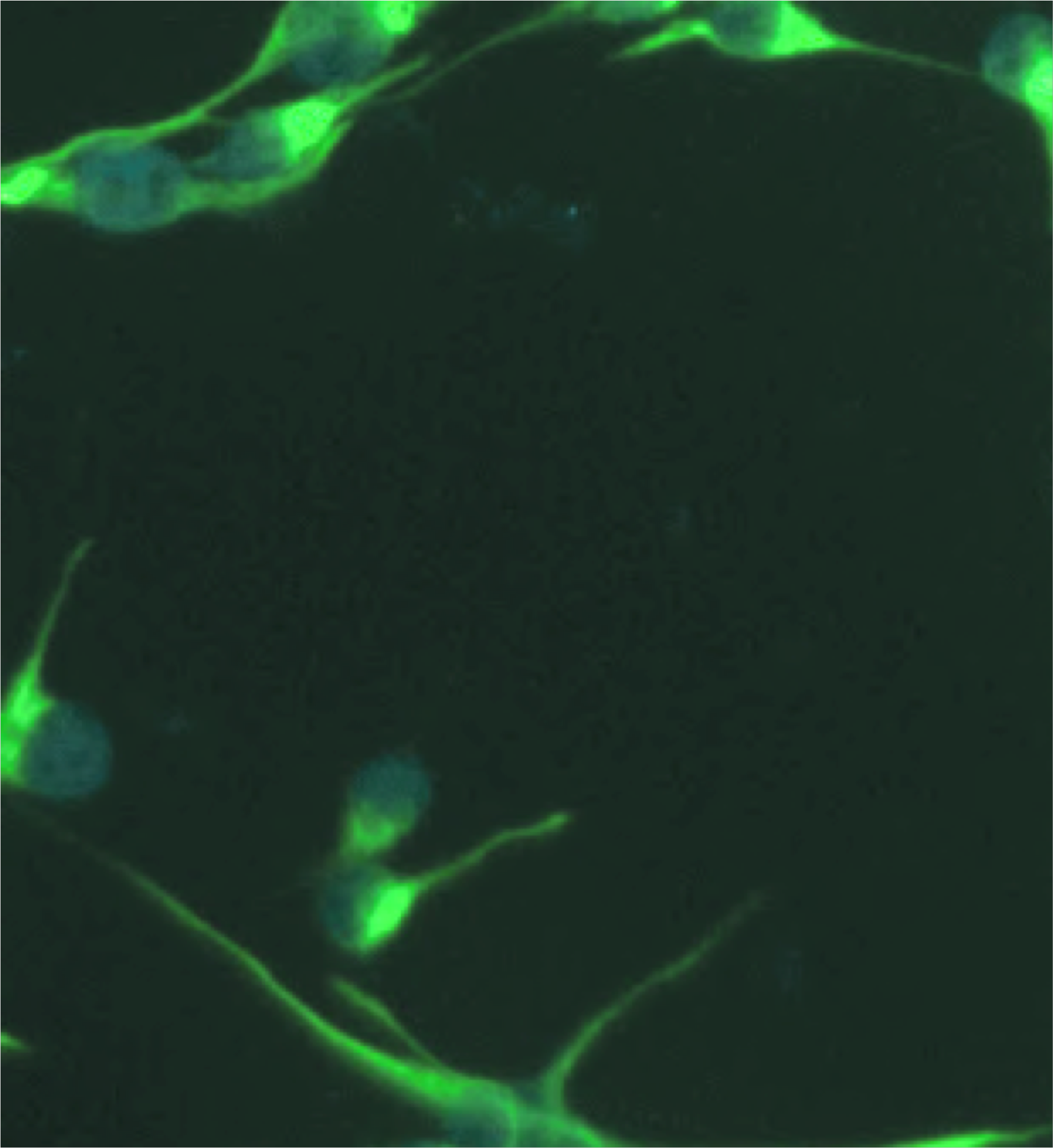

Supplement: Supplementary file 14 — Appendix Figure Source Data sd_S9 [file 44318_2025_455_MOESM14_ESM.zip › S9/B/S8i/S9_B_S8i_2.tiff]

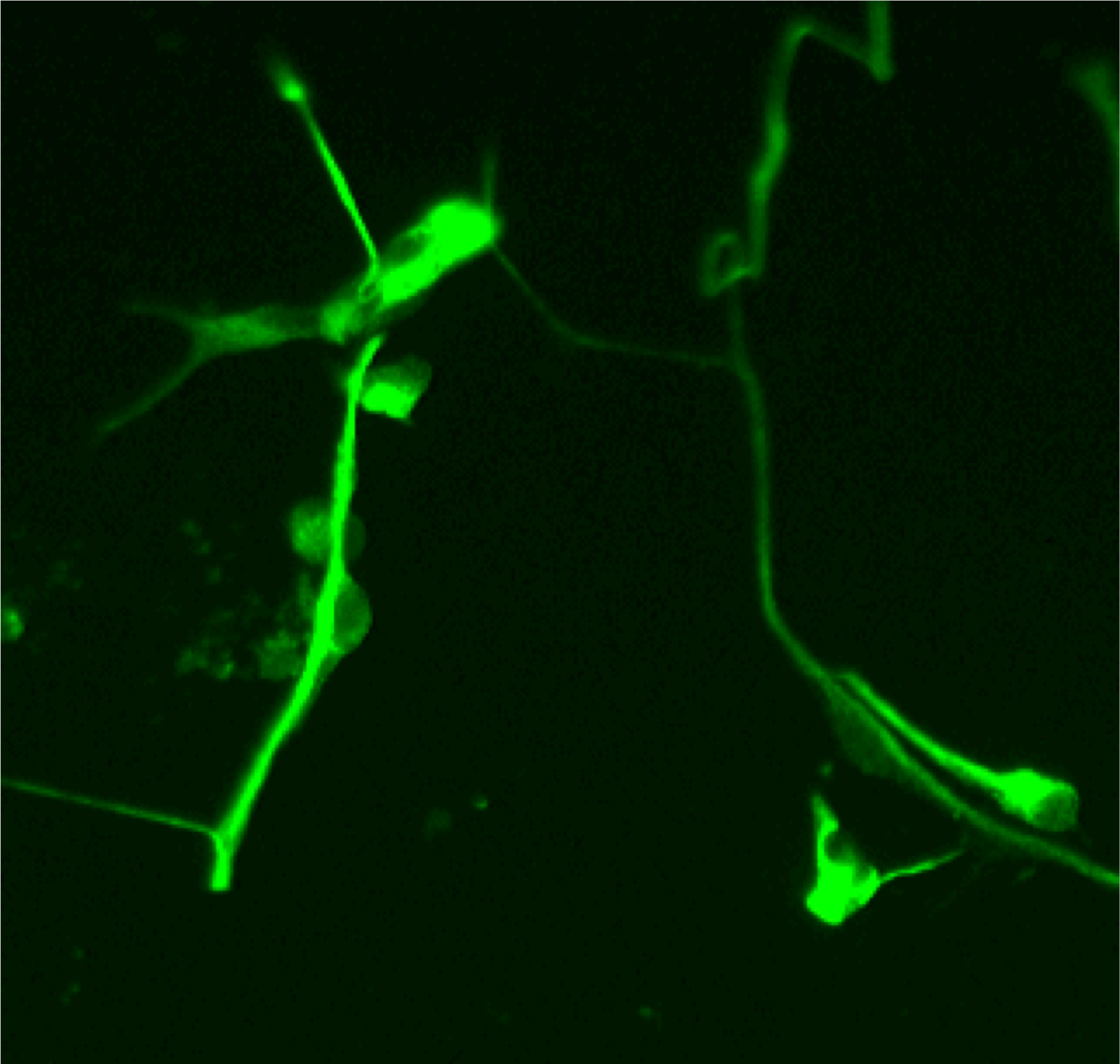

Supplement: Supplementary file 14 — Appendix Figure Source Data sd_S9 [file 44318_2025_455_MOESM14_ESM.zip › S9/C/Ctrl/S9_C_DMSO_2.tiff]

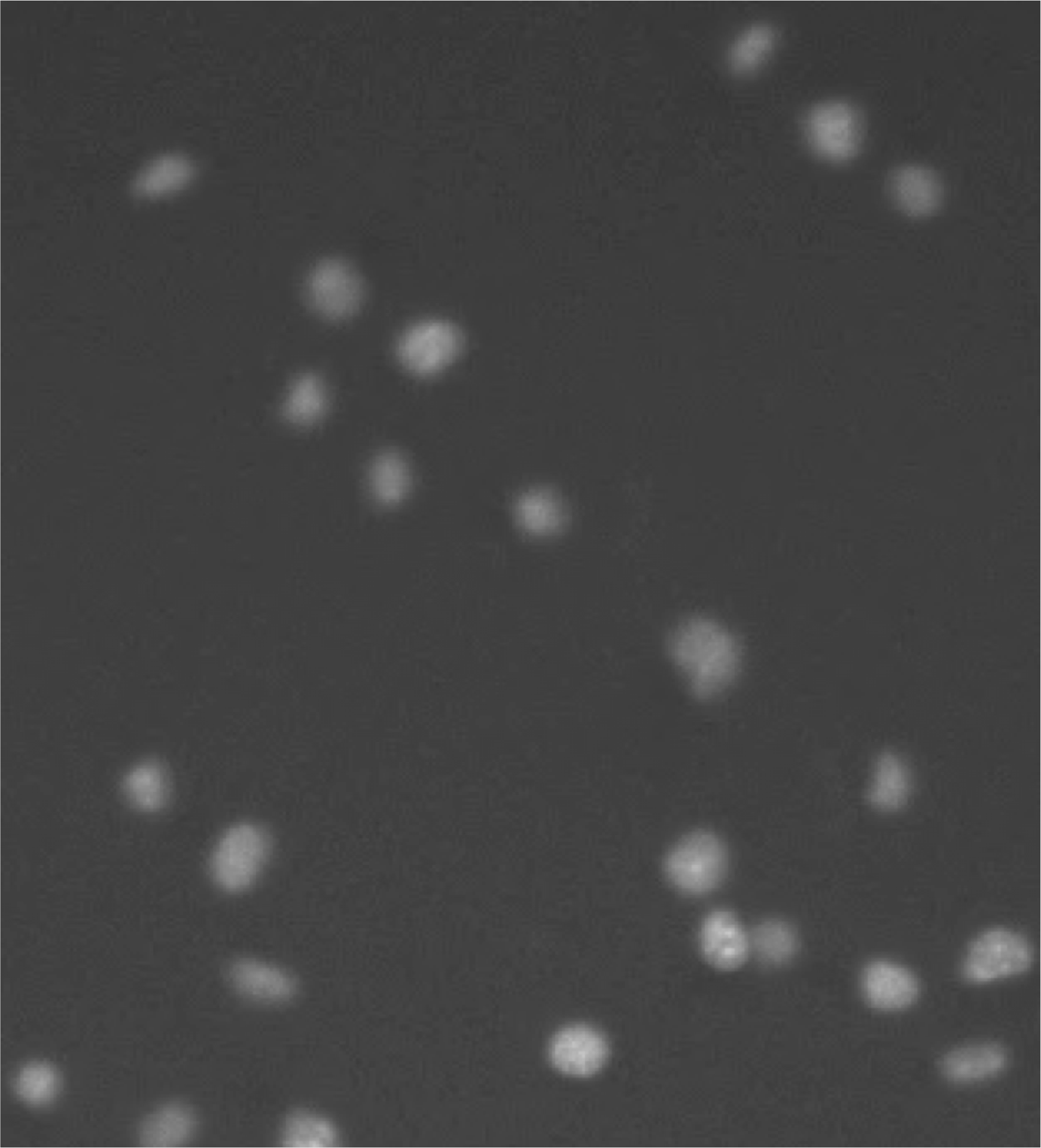

Supplement: Supplementary file 14 — Appendix Figure Source Data sd_S9 [file 44318_2025_455_MOESM14_ESM.zip › S9/B/S8iDMSO/S9_B_S8iDMSO_1.tiff]

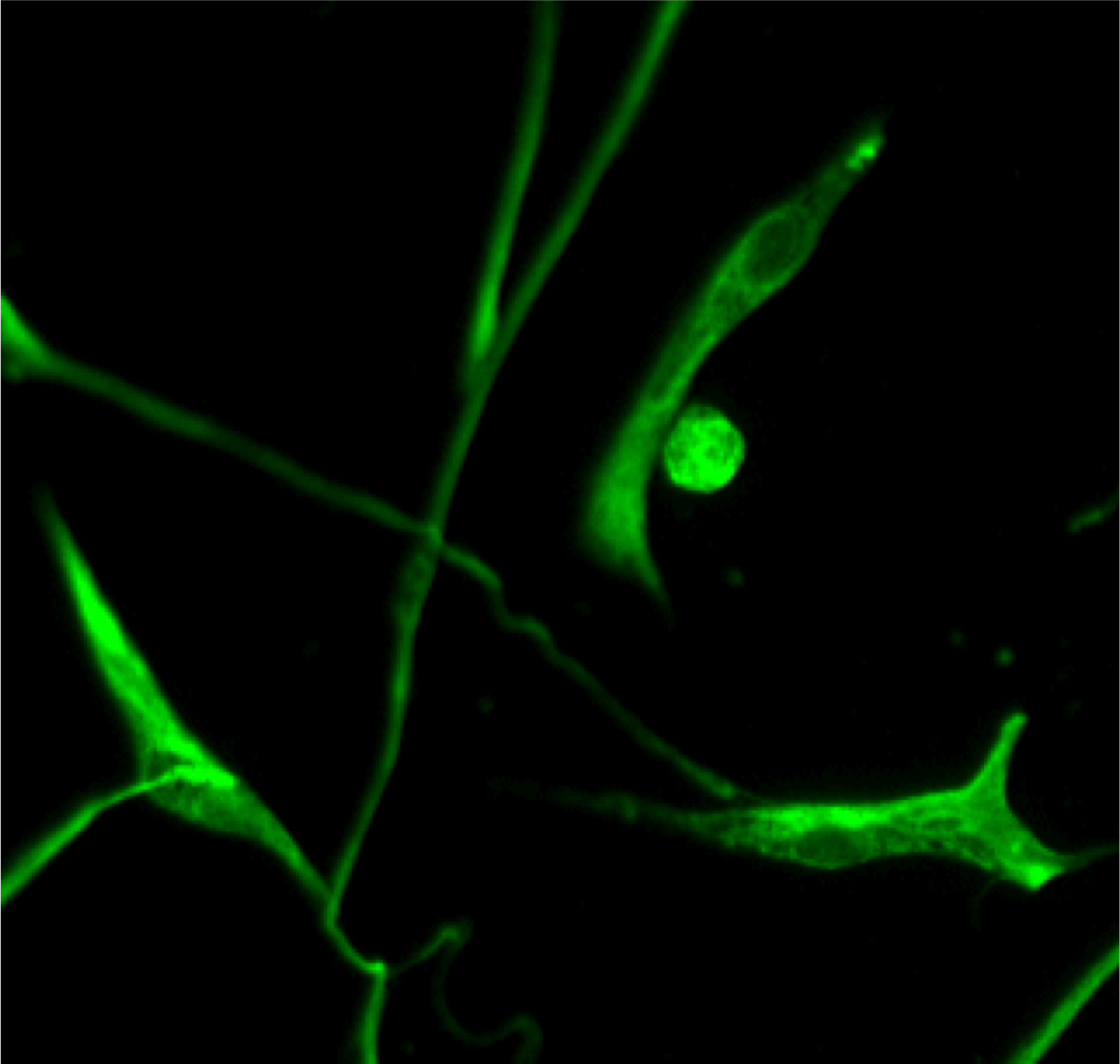

Supplement: Supplementary file 14 — Appendix Figure Source Data sd_S9 [file 44318_2025_455_MOESM14_ESM.zip › S9/C/S8i/S9_C_S8i_2.tiff]

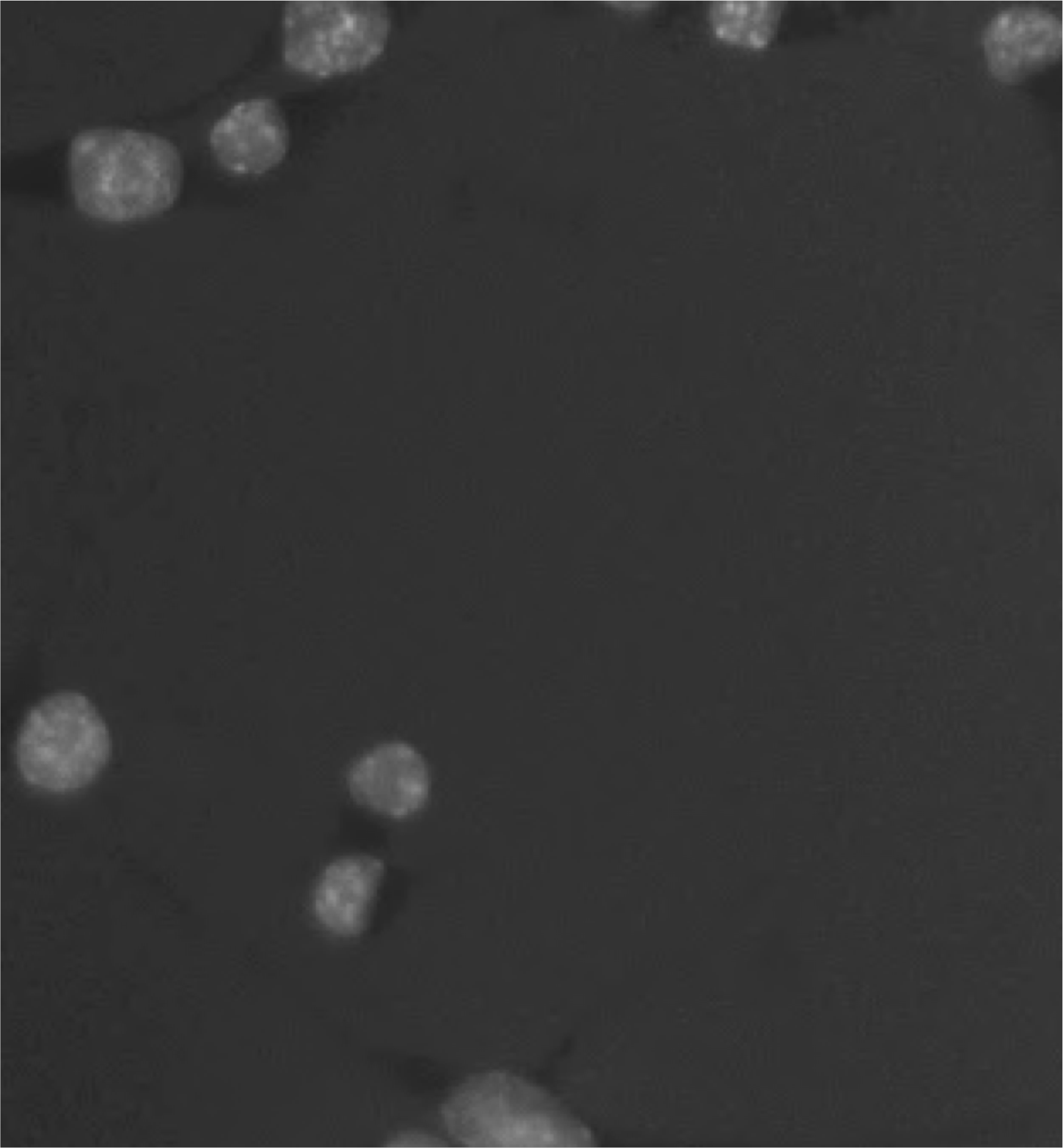

Supplement: Supplementary file 14 — Appendix Figure Source Data sd_S9 [file 44318_2025_455_MOESM14_ESM.zip › S9/B/S8i/S9_B_S8i_1.tiff]

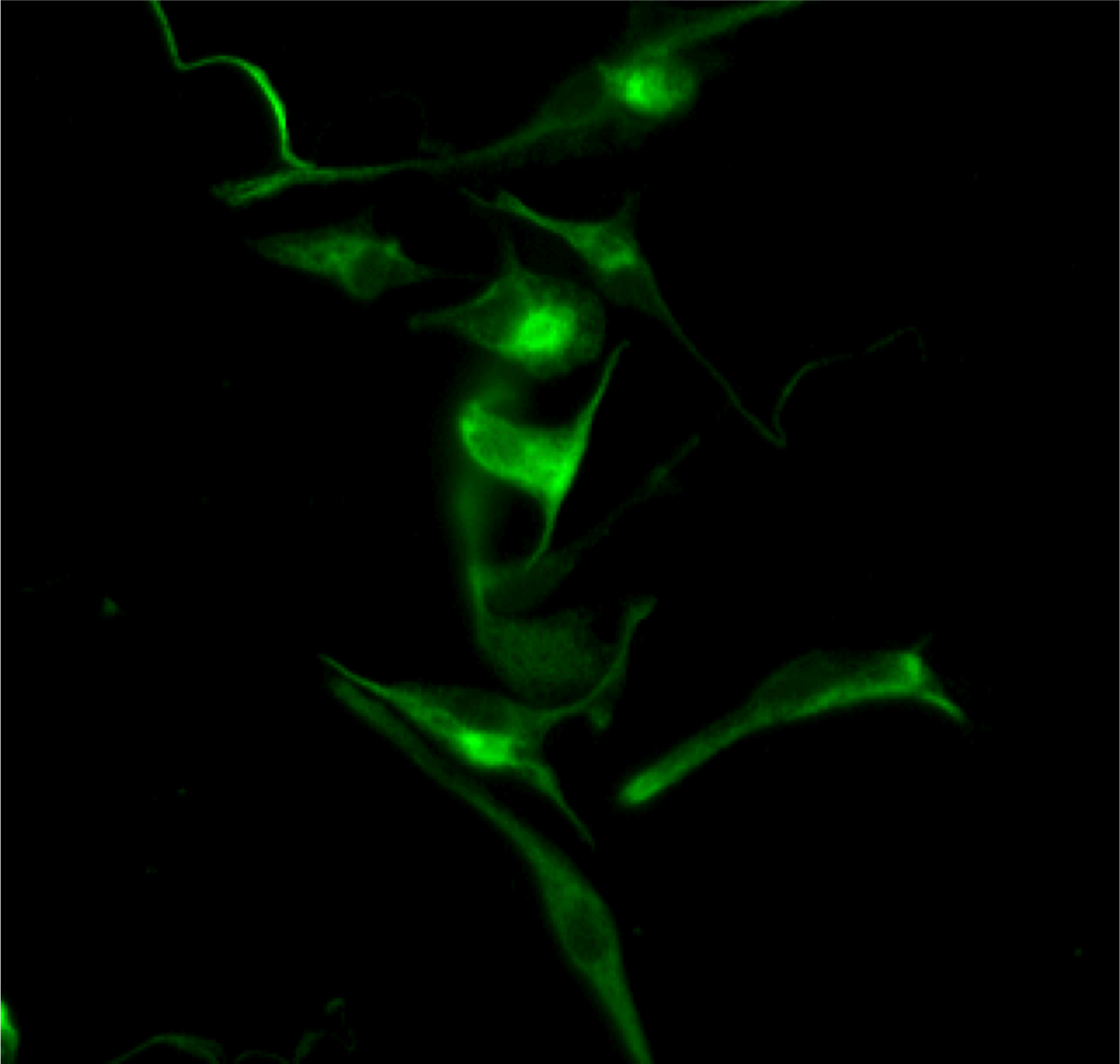

Supplement: Supplementary file 14 — Appendix Figure Source Data sd_S9 [file 44318_2025_455_MOESM14_ESM.zip › S9/C/S8iDMSO/S9_C_S8iDMSO_2.tiff]

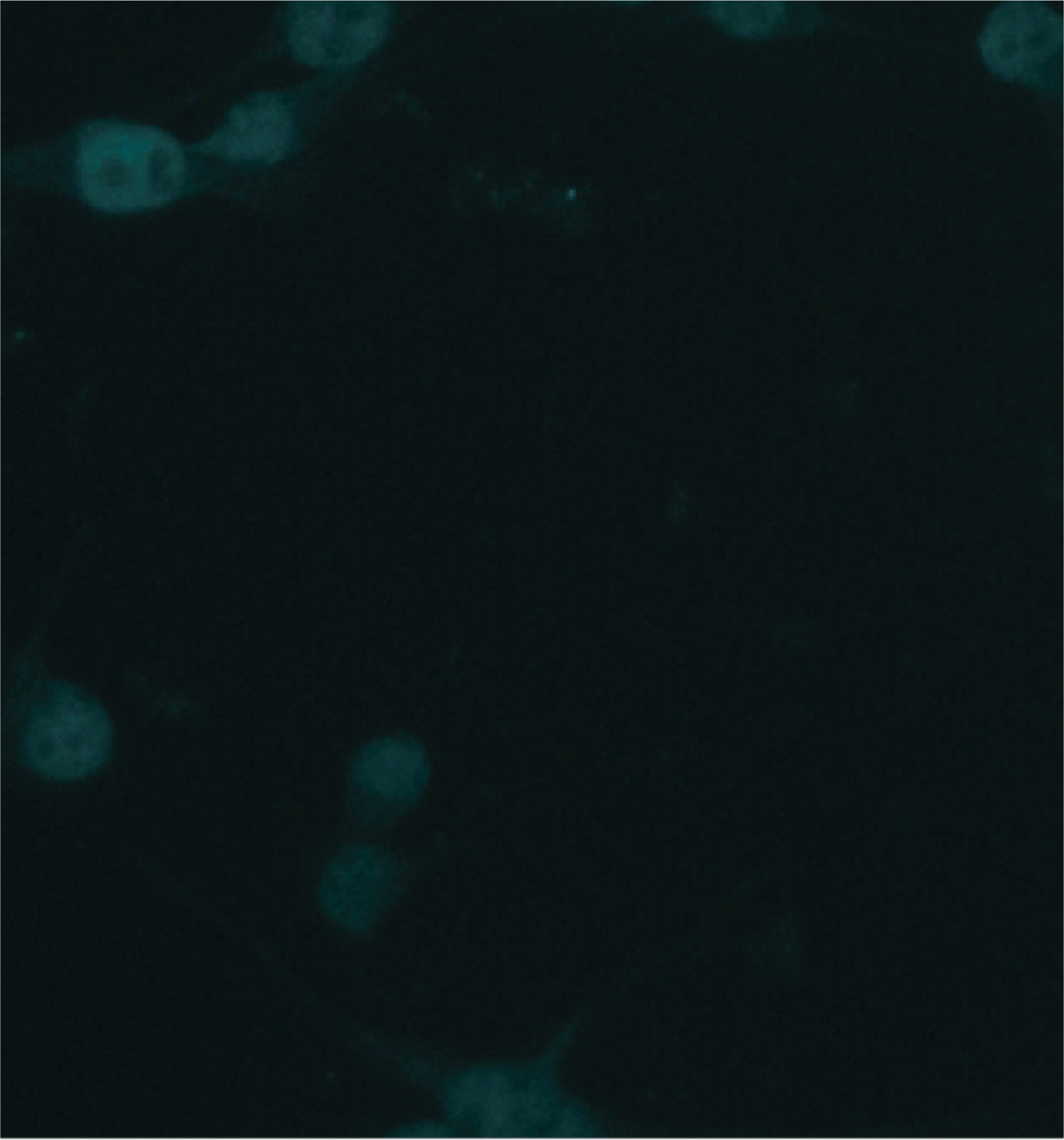

Supplement: Supplementary file 14 — Appendix Figure Source Data sd_S9 [file 44318_2025_455_MOESM14_ESM.zip › S9/B/S8i/S9_B_S8i_3.tiff]

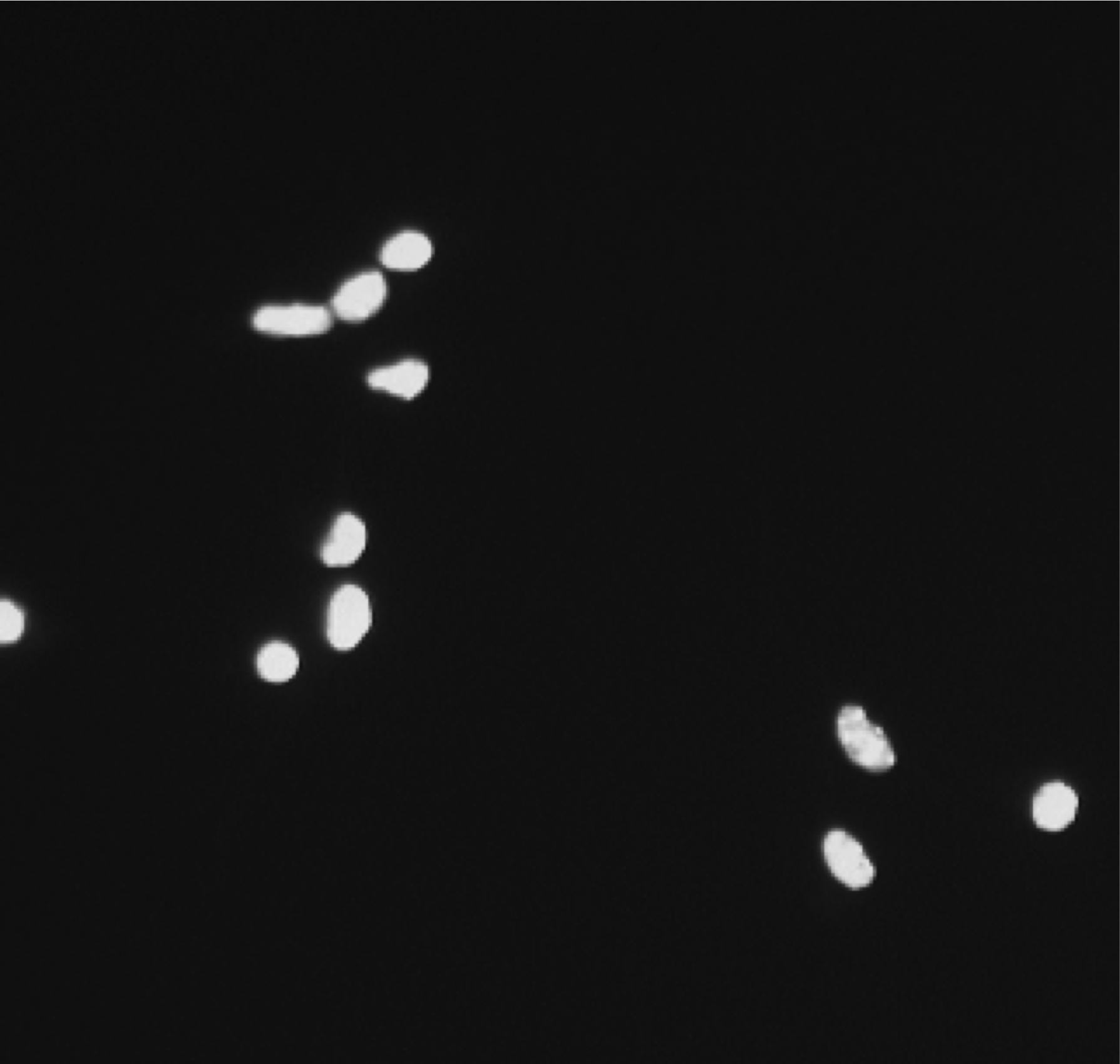

Supplement: Supplementary file 14 — Appendix Figure Source Data sd_S9 [file 44318_2025_455_MOESM14_ESM.zip › S9/C/Ctrl/S9_C_DMSO_1.tiff]

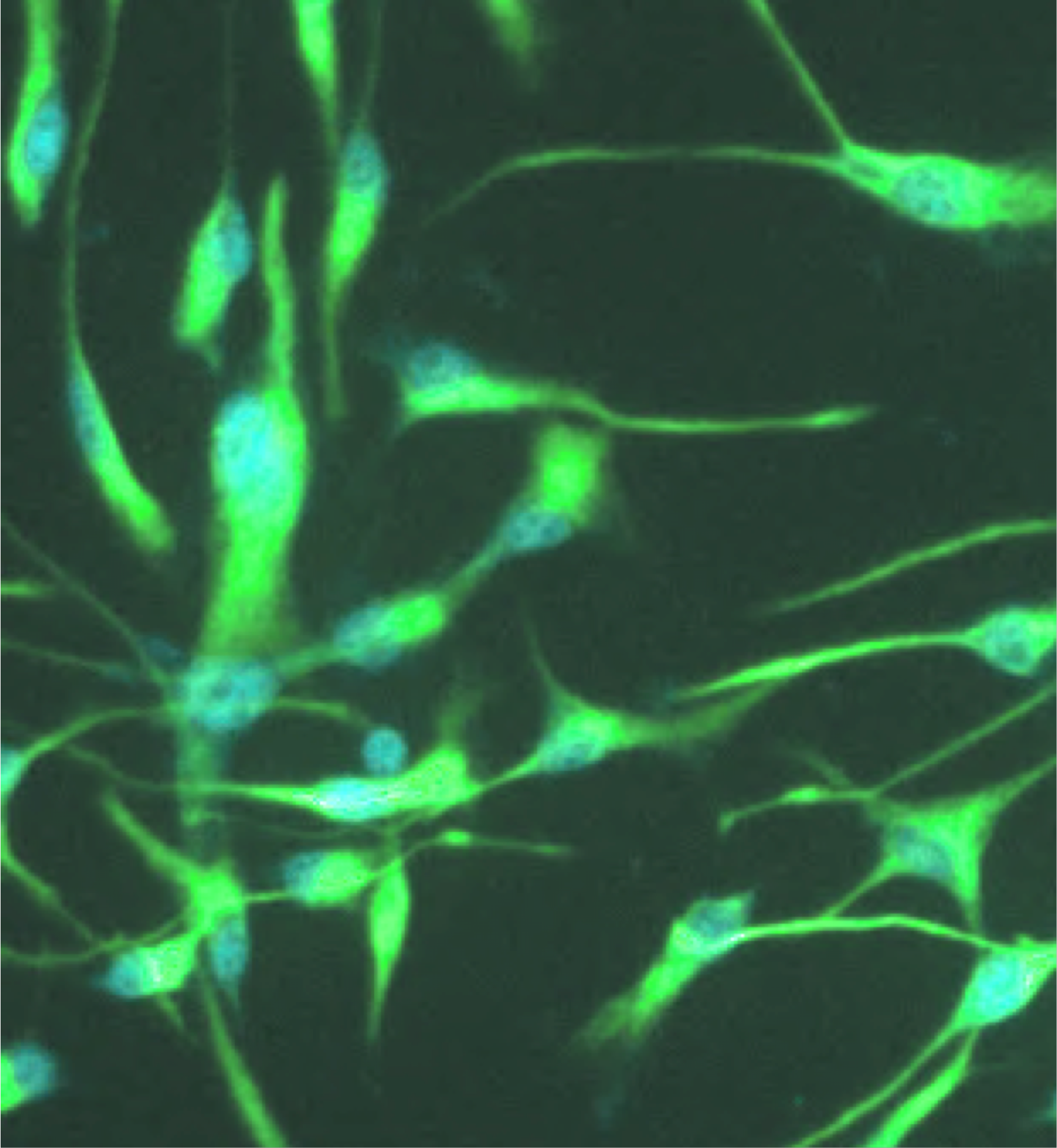

Supplement: Supplementary file 14 — Appendix Figure Source Data sd_S9 [file 44318_2025_455_MOESM14_ESM.zip › S9/B/DMSO/S9_B_DMSO_2.tiff]

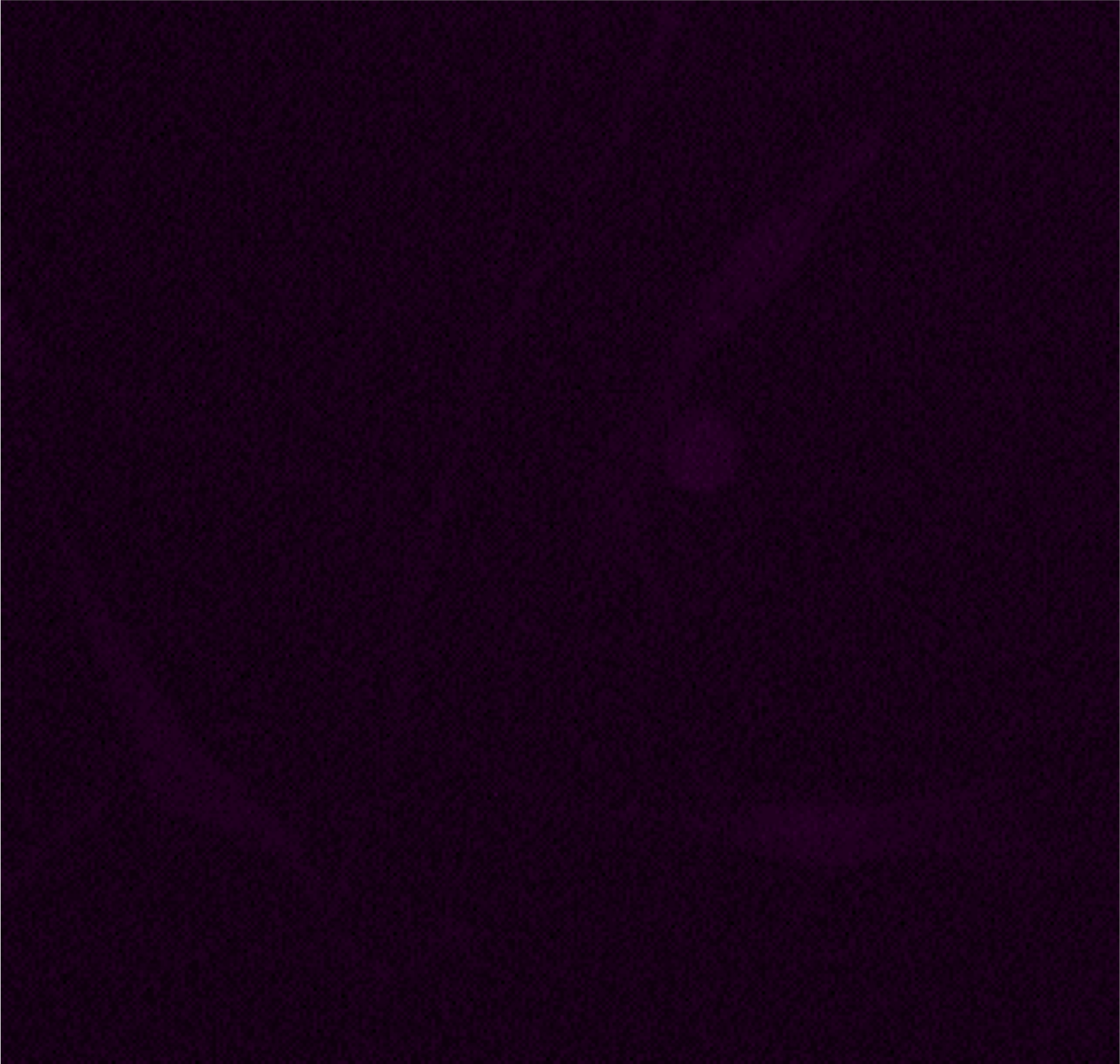

Supplement: Supplementary file 14 — Appendix Figure Source Data sd_S9 [file 44318_2025_455_MOESM14_ESM.zip › S9/C/S8i/S9_C_S8i_3.tiff]

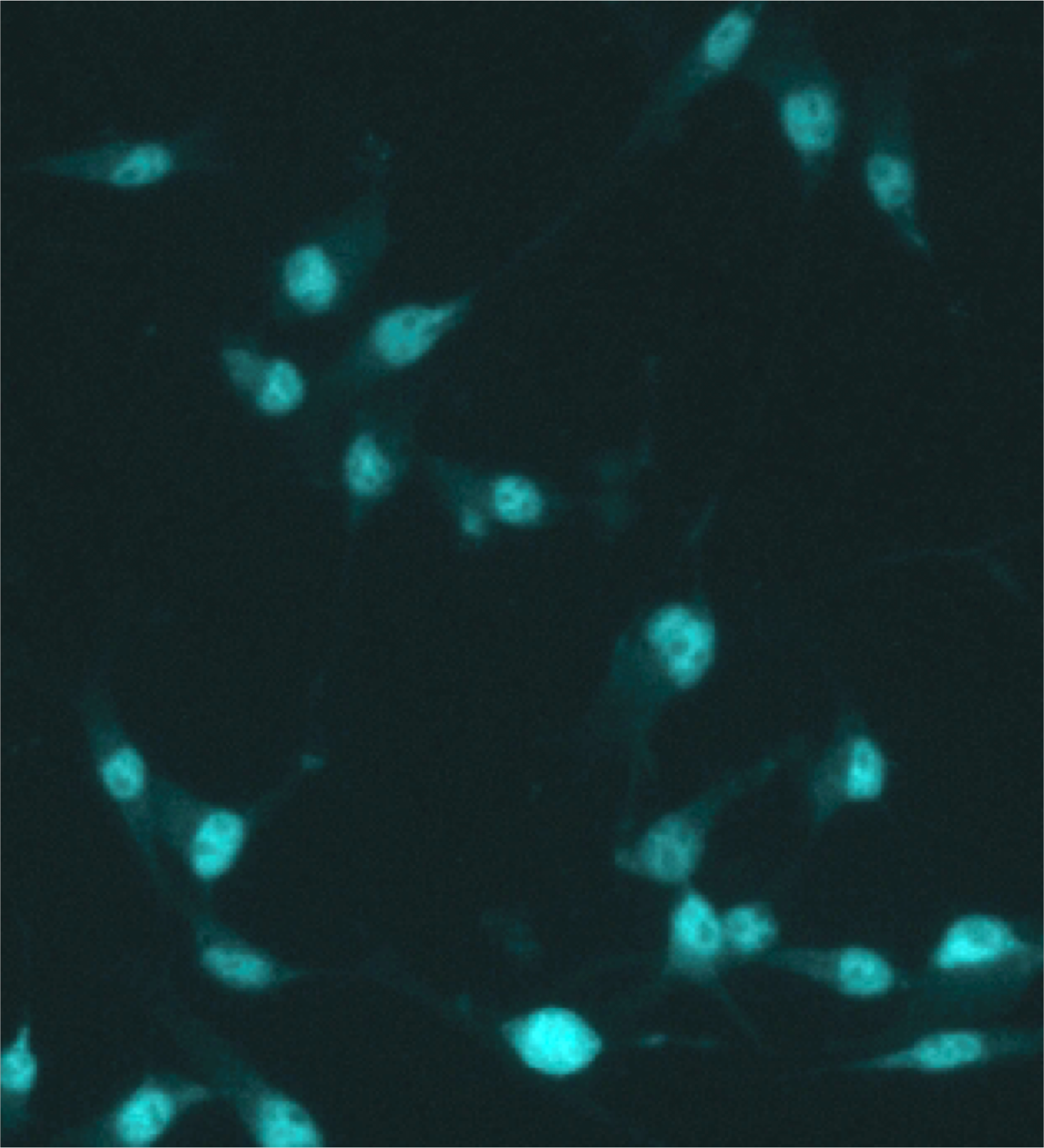

Supplement: Supplementary file 14 — Appendix Figure Source Data sd_S9 [file 44318_2025_455_MOESM14_ESM.zip › S9/B/S8iDMSO/S9_B_S8iDMSO_3.tiff]

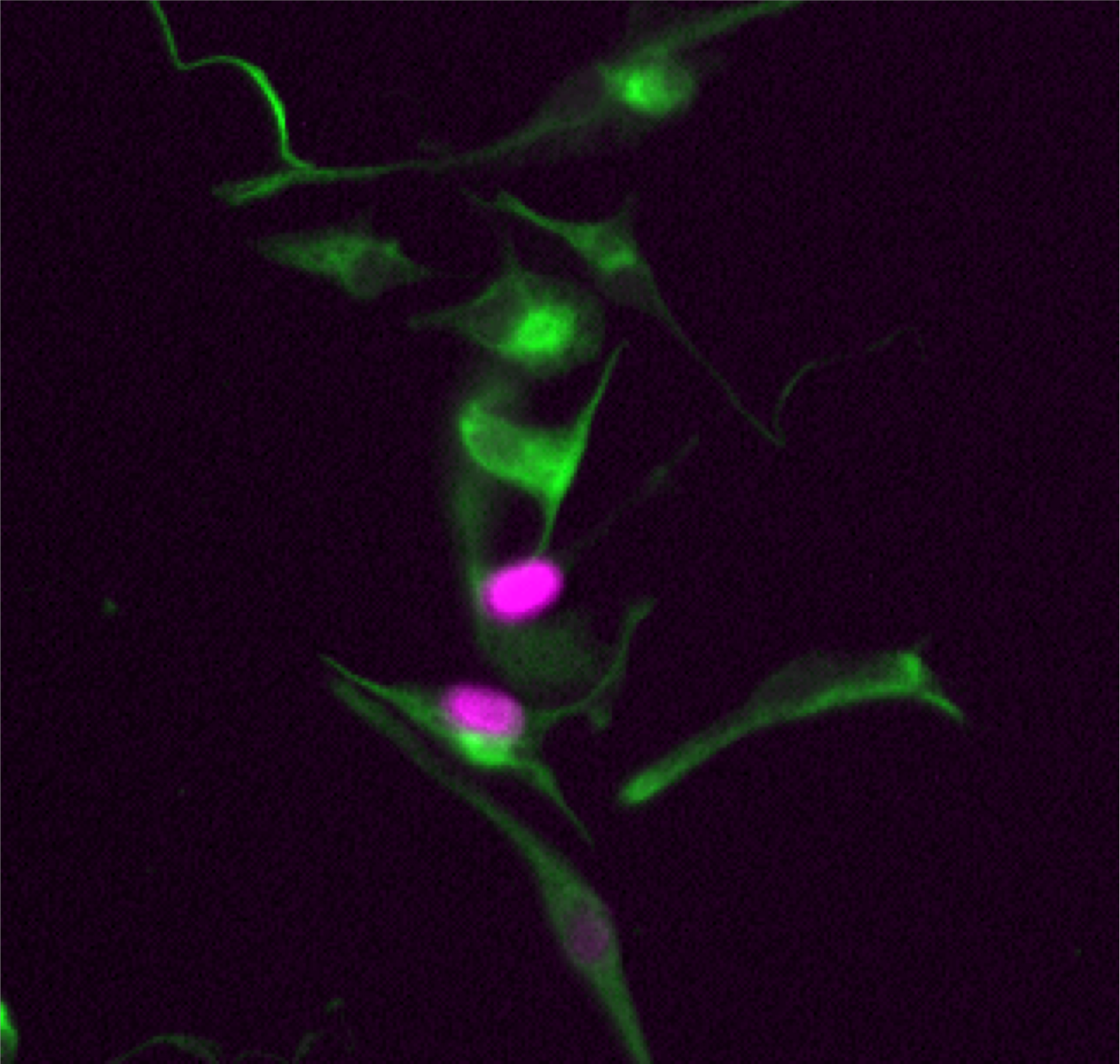

Supplement: Supplementary file 14 — Appendix Figure Source Data sd_S9 [file 44318_2025_455_MOESM14_ESM.zip › S9/C/S8iDMSO/S9_C_S8iDMSO_4.tiff]

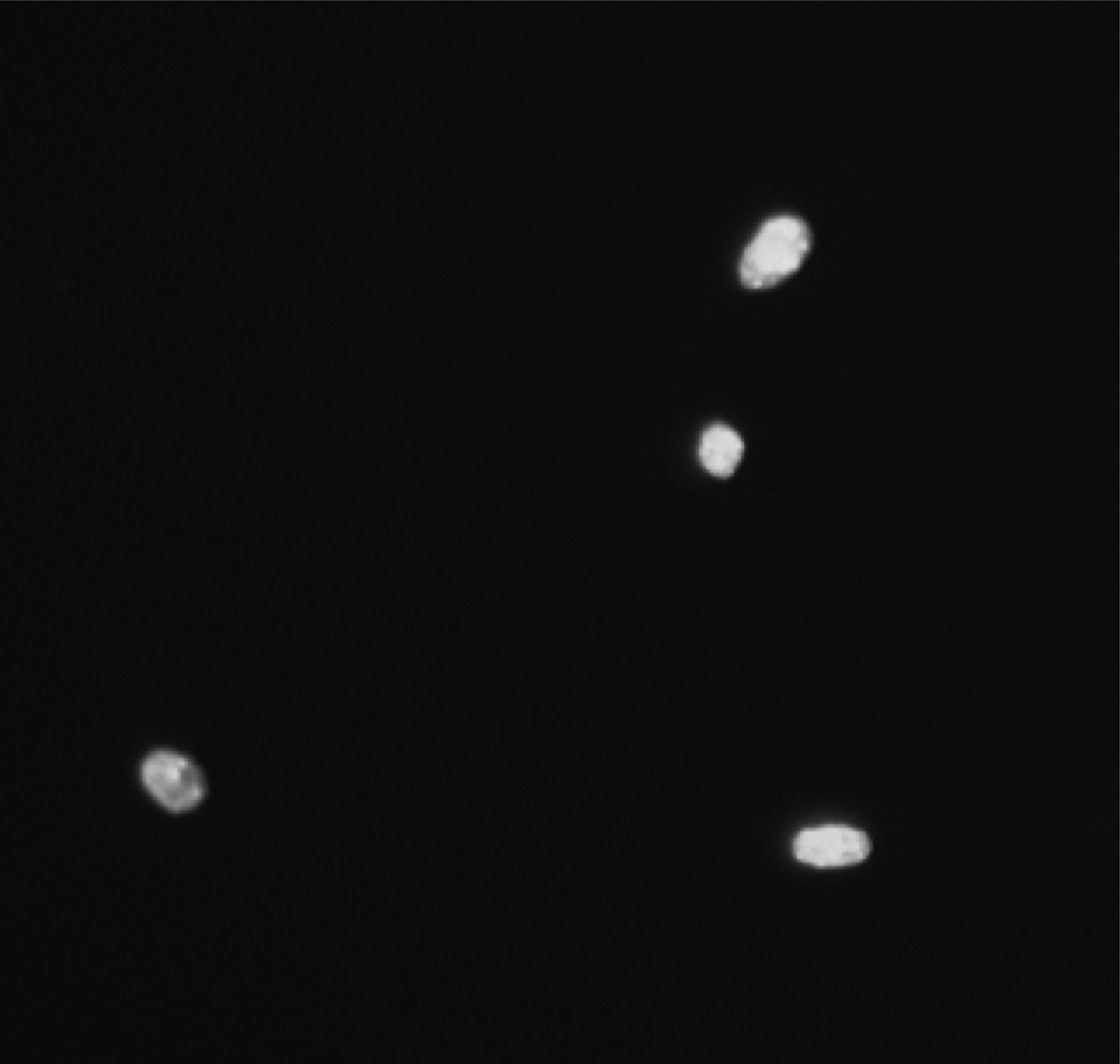

Supplement: Supplementary file 14 — Appendix Figure Source Data sd_S9 [file 44318_2025_455_MOESM14_ESM.zip › S9/C/S8i/S9_C_S8i_1.tiff]

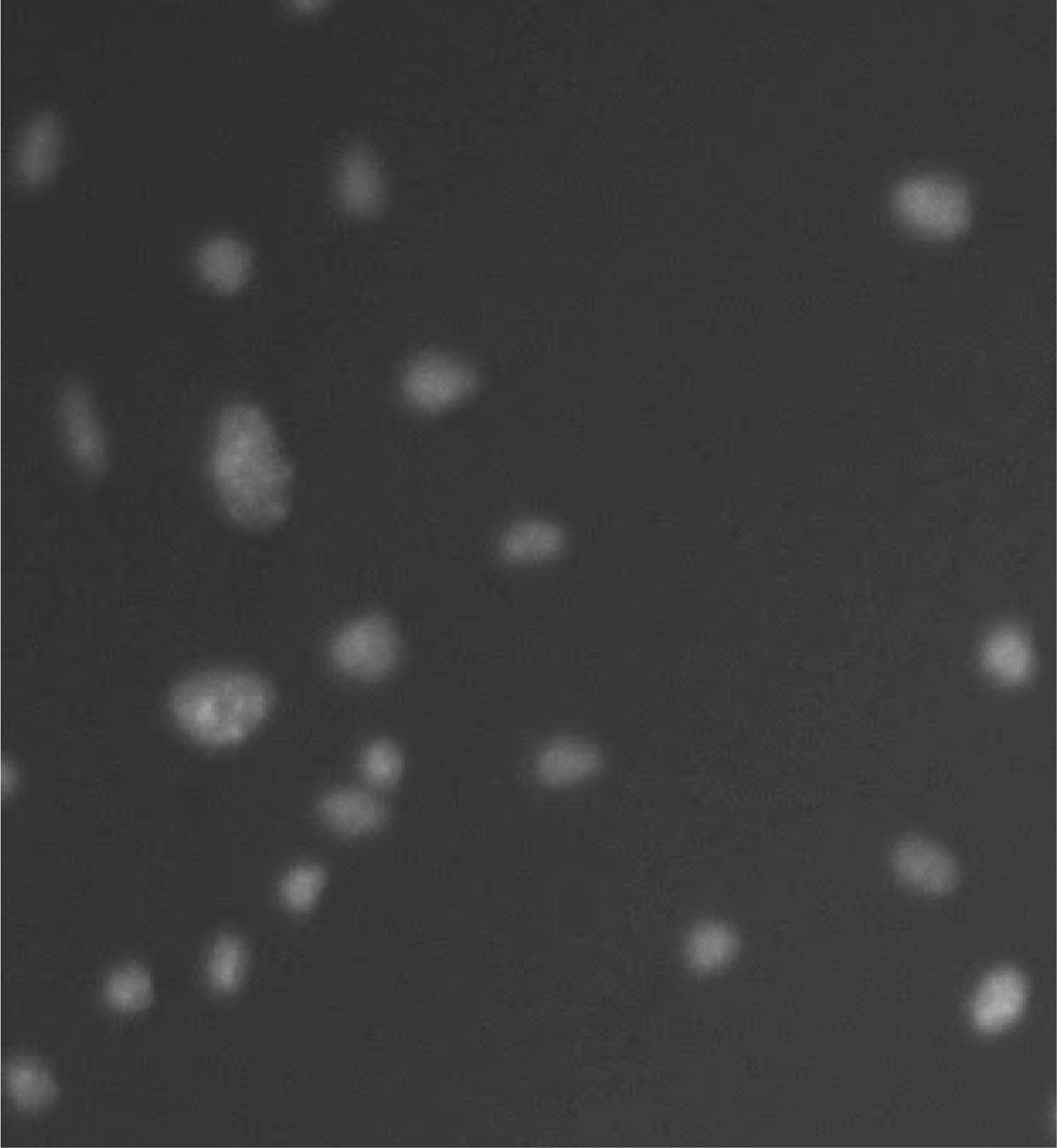

Supplement: Supplementary file 14 — Appendix Figure Source Data sd_S9 [file 44318_2025_455_MOESM14_ESM.zip › S9/B/DMSO/S9_B_DMSO_1.tiff]

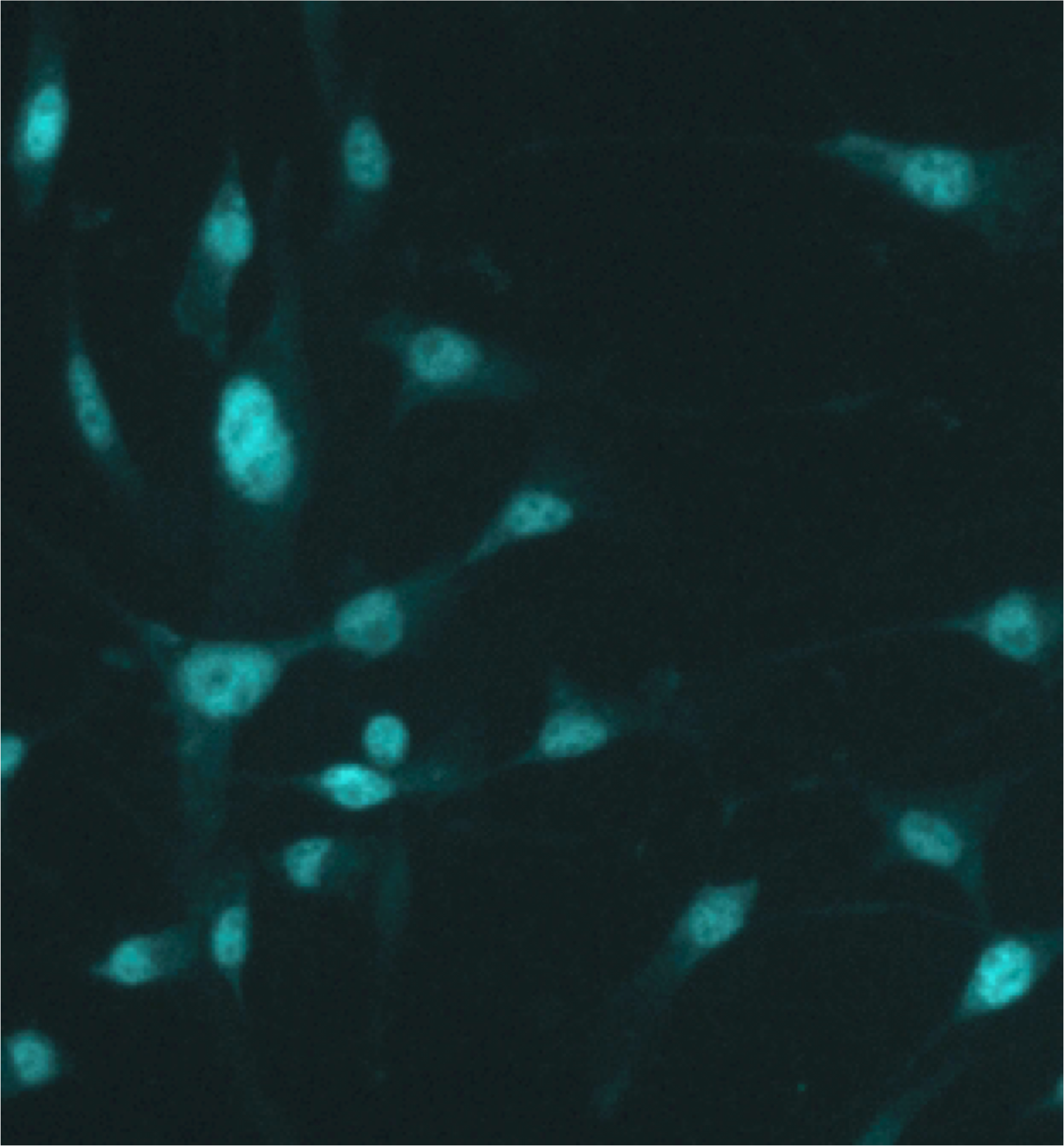

Supplement: Supplementary file 14 — Appendix Figure Source Data sd_S9 [file 44318_2025_455_MOESM14_ESM.zip › S9/B/DMSO/S9_B_DMSO_3.tiff]

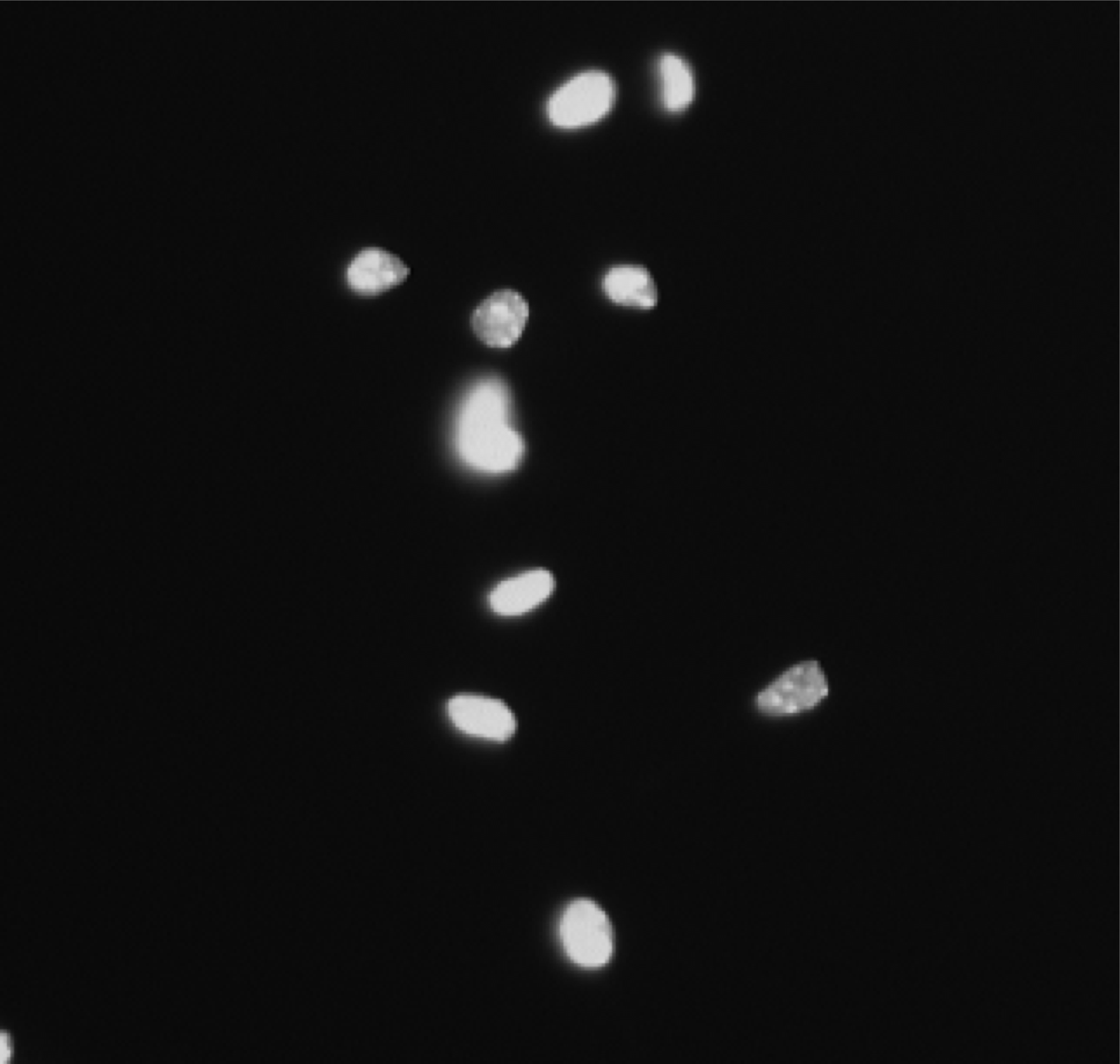

Supplement: Supplementary file 14 — Appendix Figure Source Data sd_S9 [file 44318_2025_455_MOESM14_ESM.zip › S9/C/S8iDMSO/S9_C_S8iDMSO_1.tiff]

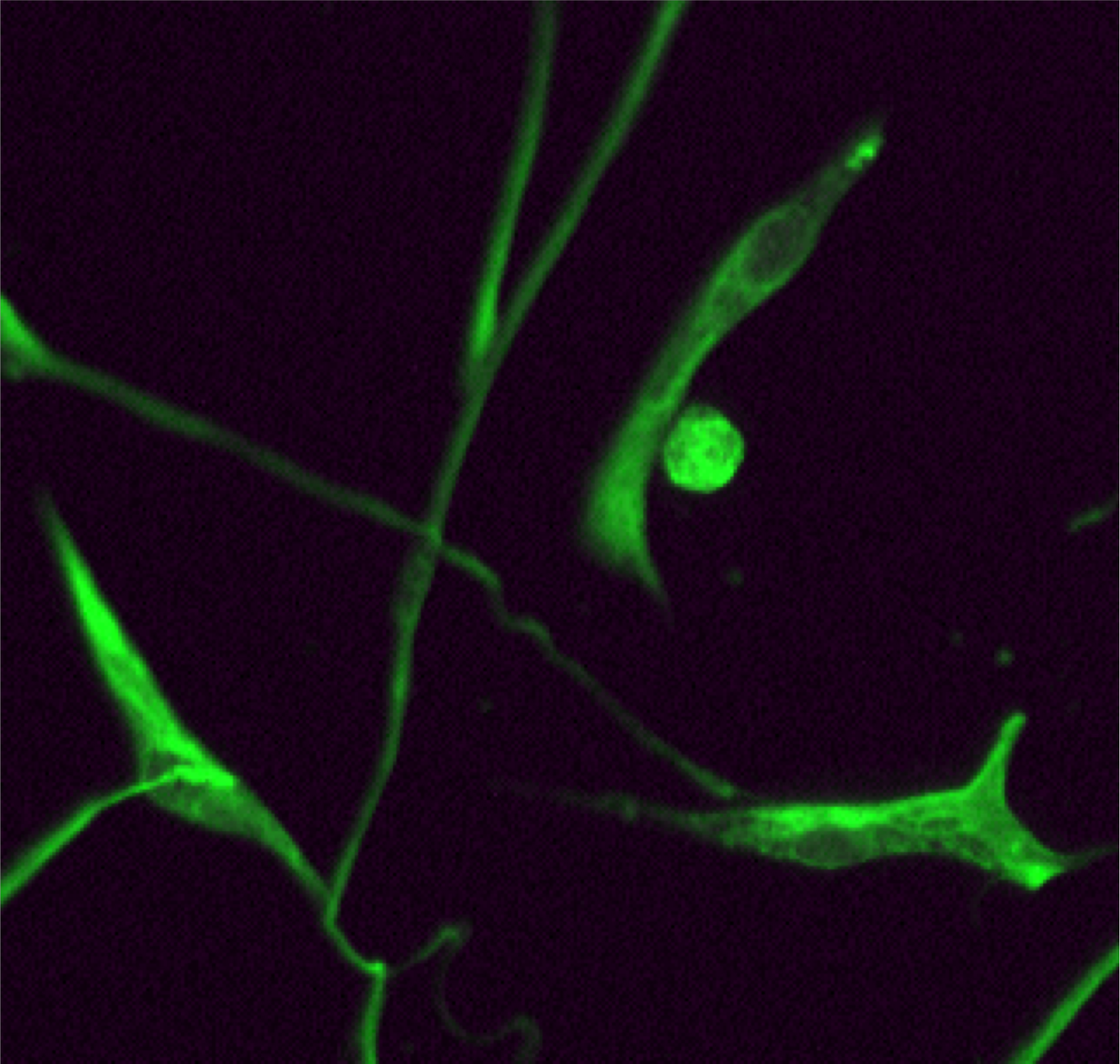

Supplement: Supplementary file 14 — Appendix Figure Source Data sd_S9 [file 44318_2025_455_MOESM14_ESM.zip › S9/C/S8i/S9_C_S8i_4.tiff]

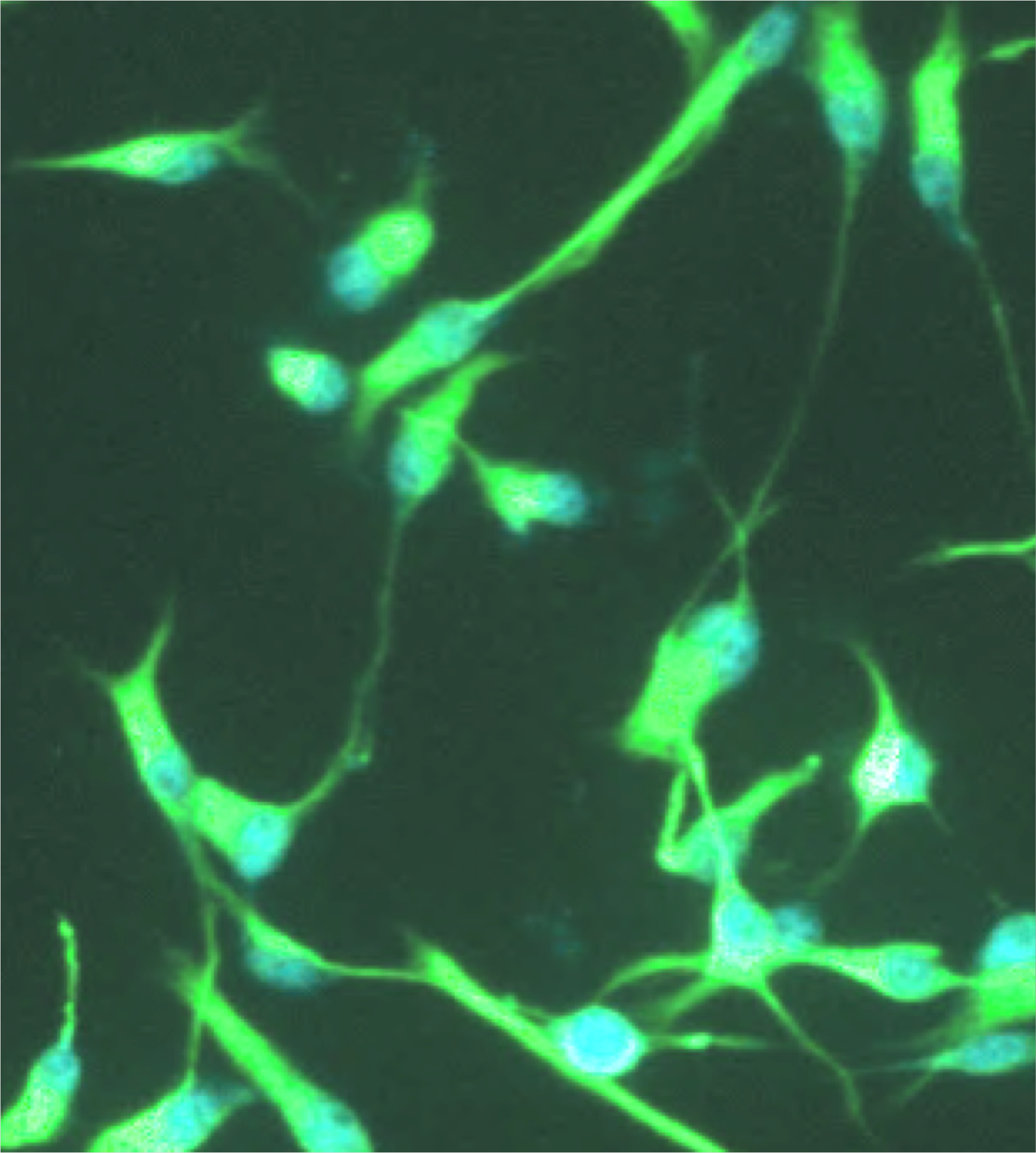

Supplement: Supplementary file 14 — Appendix Figure Source Data sd_S9 [file 44318_2025_455_MOESM14_ESM.zip › S9/B/S8iDMSO/S9_B_S8iDMSO_2.tiff]

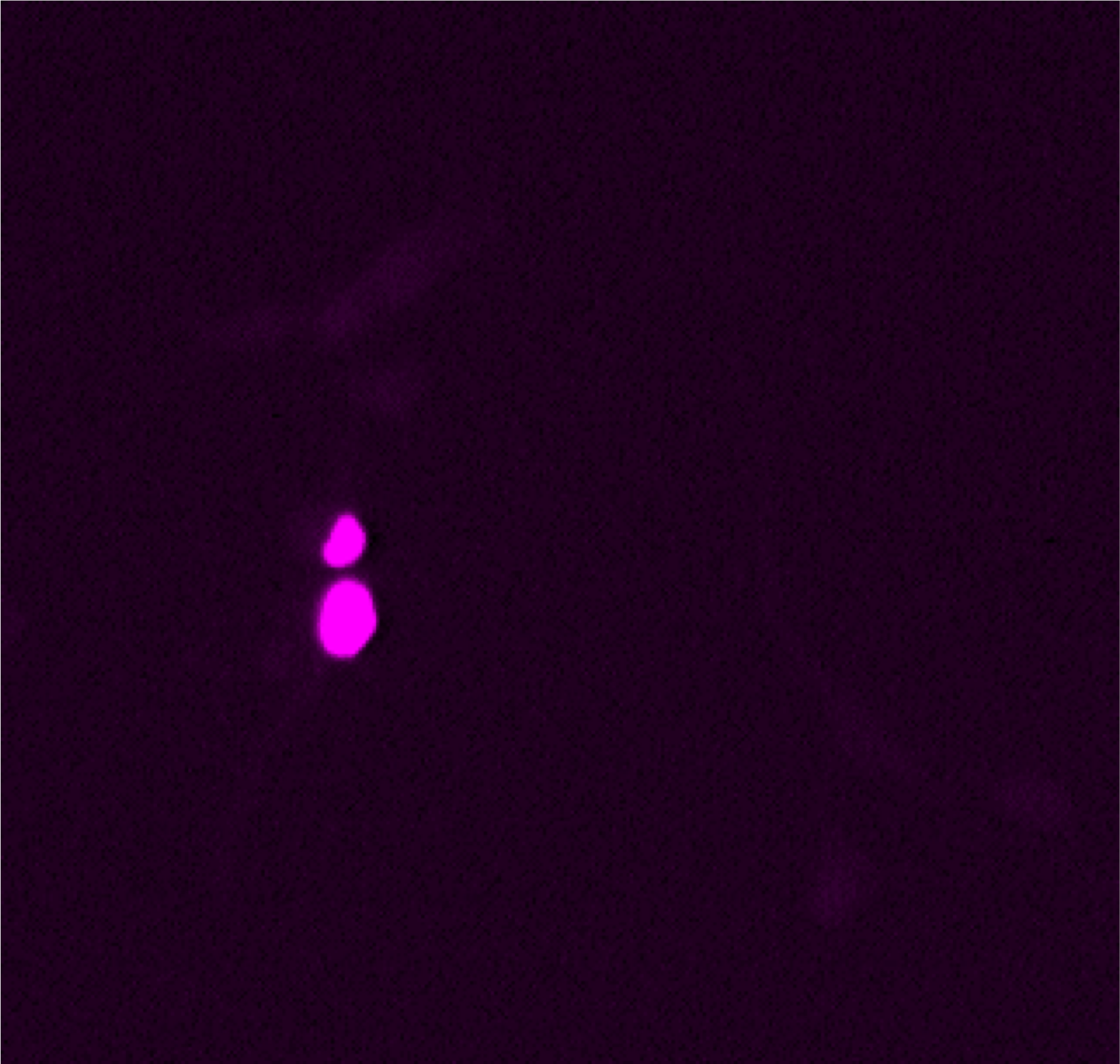

Supplement: Supplementary file 14 — Appendix Figure Source Data sd_S9 [file 44318_2025_455_MOESM14_ESM.zip › S9/C/Ctrl/S9_C_DMSO_3.tiff]

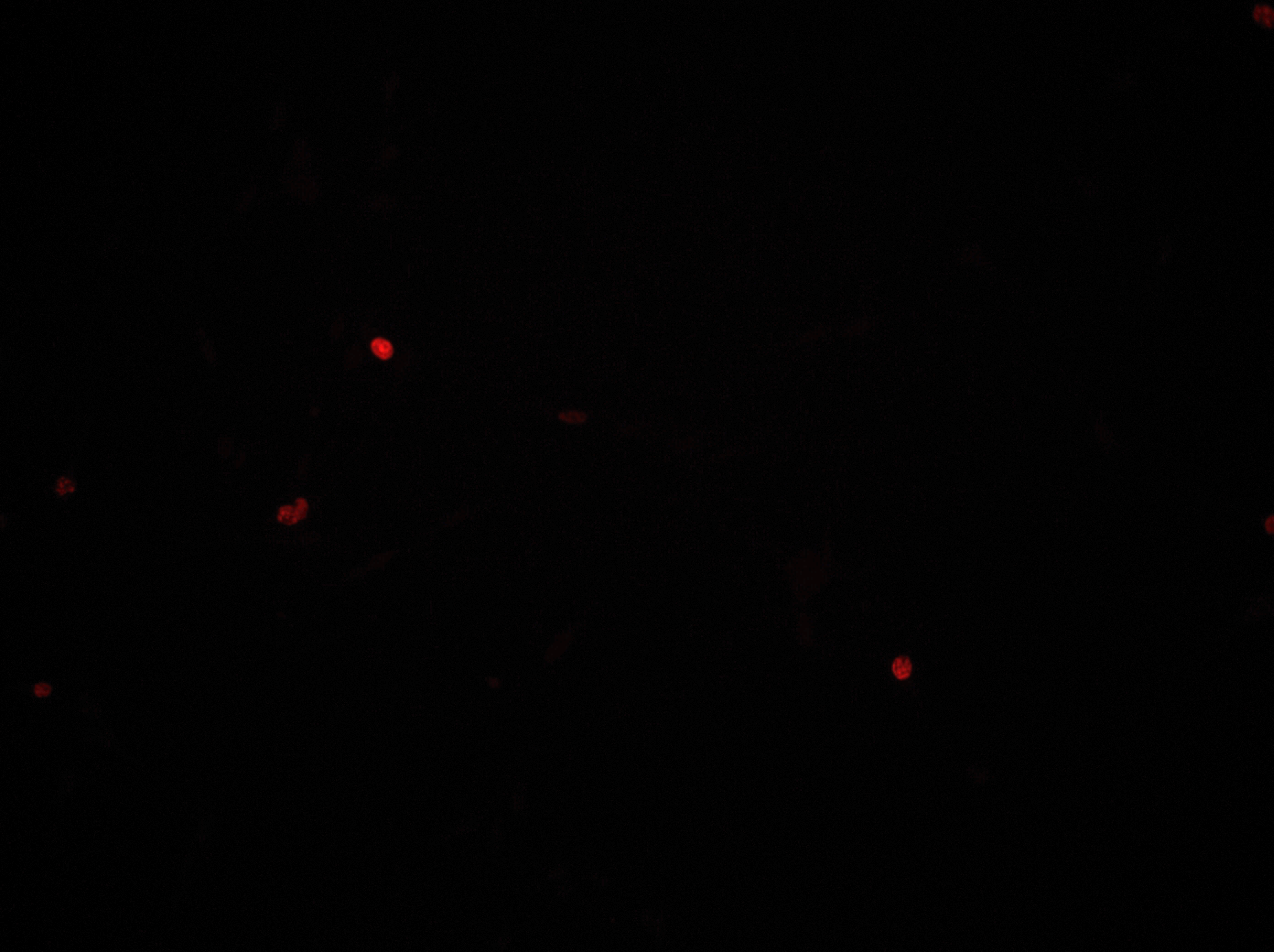

Supplement: Supplementary file 14 — Appendix Figure Source Data sd_S9 [file 44318_2025_455_MOESM14_ESM.zip › S9/E/S8i/S9_E_S8i_2.tiff]

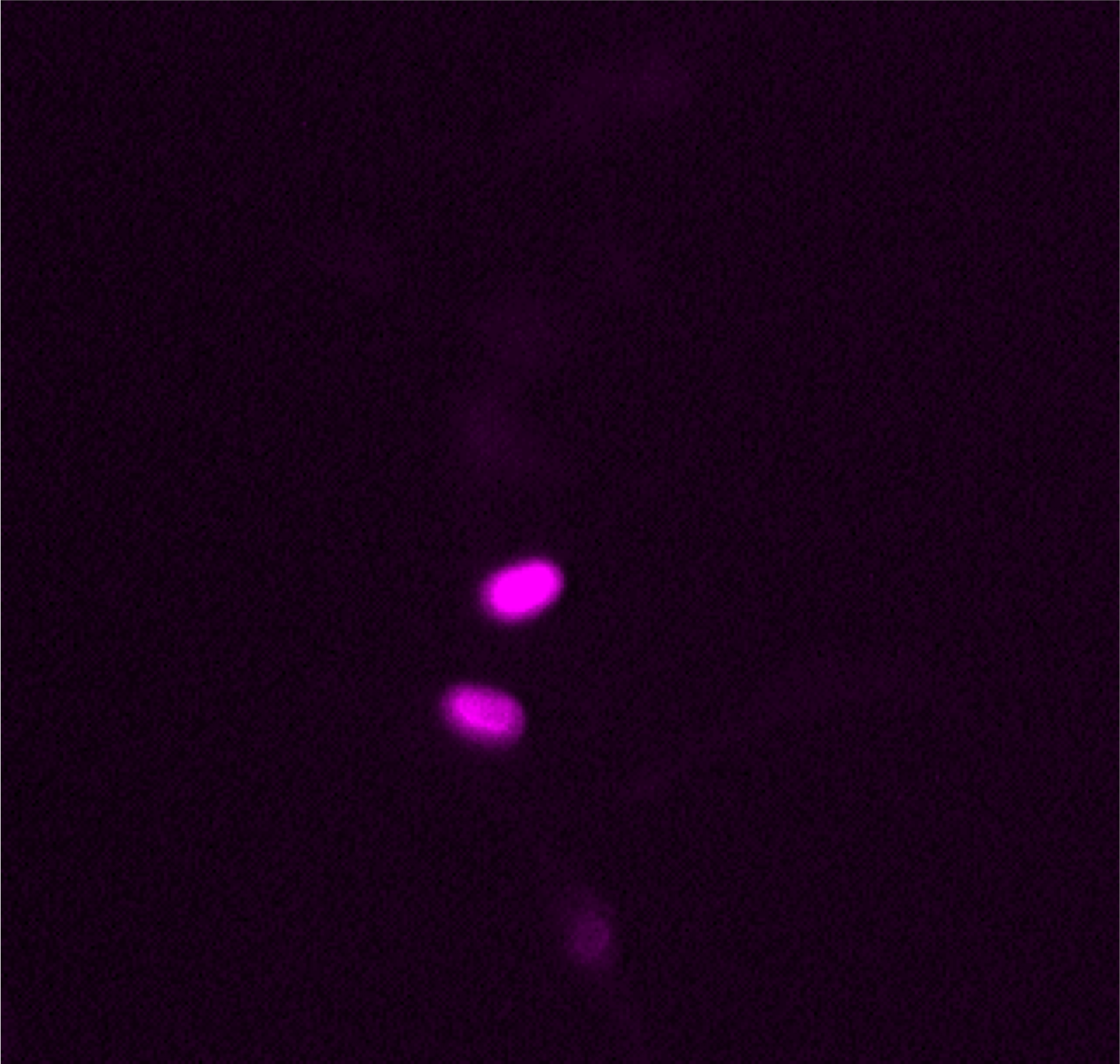

Supplement: Supplementary file 14 — Appendix Figure Source Data sd_S9 [file 44318_2025_455_MOESM14_ESM.zip › S9/C/S8iDMSO/S9_C_S8iDMSO_3.tiff]

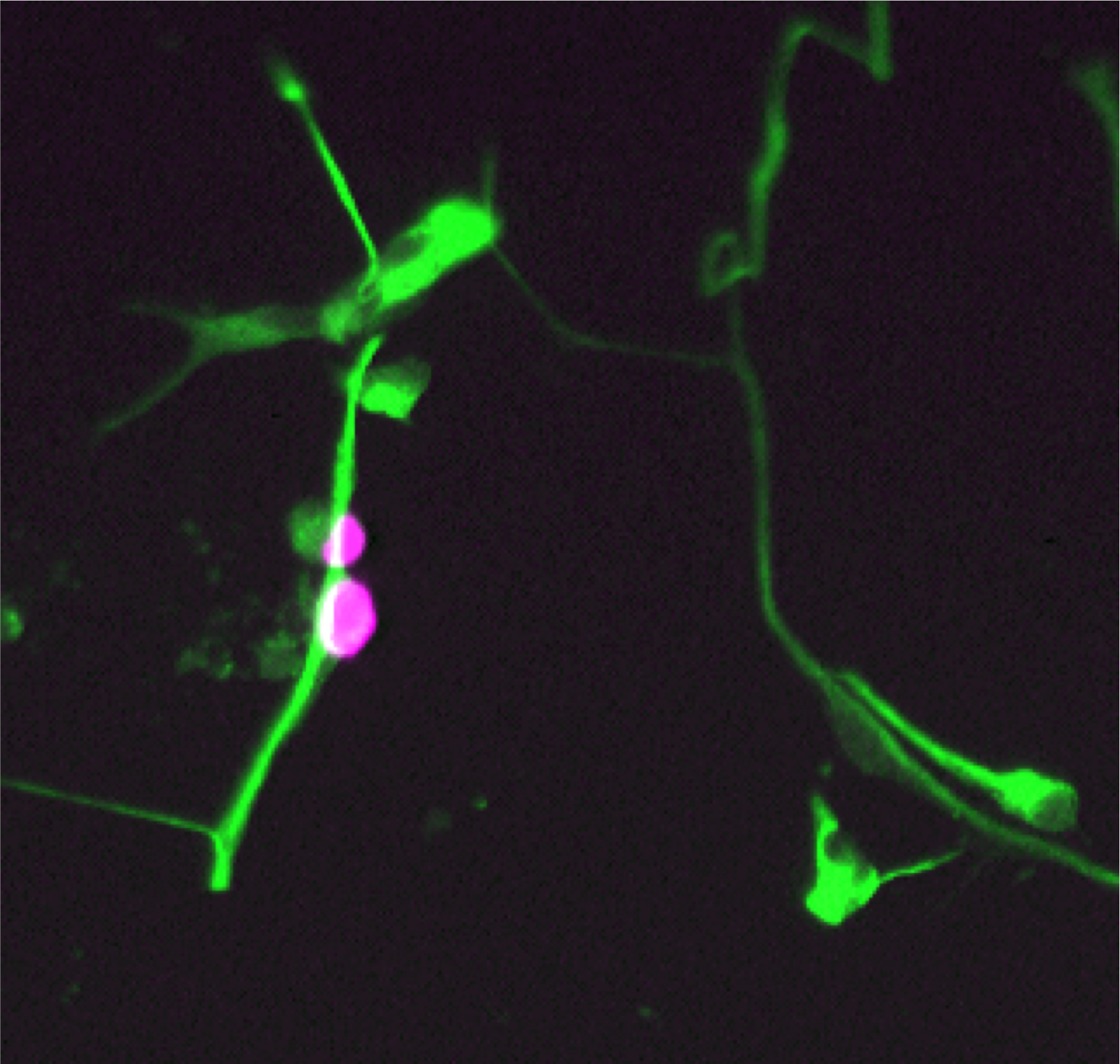

Supplement: Supplementary file 14 — Appendix Figure Source Data sd_S9 [file 44318_2025_455_MOESM14_ESM.zip › S9/C/Ctrl/S9_C_DMSO_4.tiff]

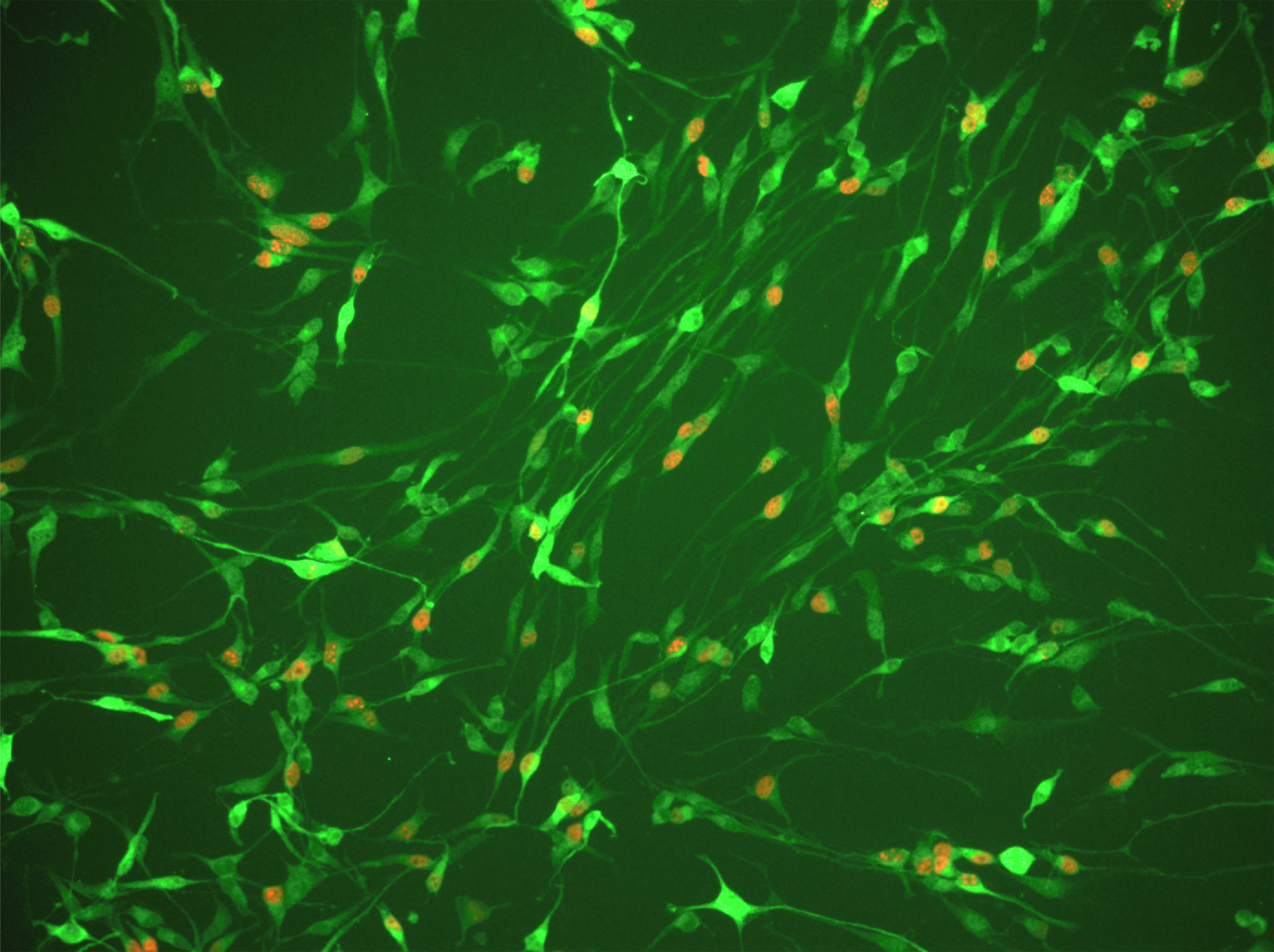

Supplement: Supplementary file 14 — Appendix Figure Source Data sd_S9 [file 44318_2025_455_MOESM14_ESM.zip › S9/E/Ctrl/S9_E_DMSO_3.tiff]
