# Supplementary figures and images for: Epigenetic regulation of neural stem cell aging in the mouse hippocampus by Setd8 downregulation (part 2 of 2)
Source: EMBO J. 2025 Jun 3;44(13):3645–68. doi: 10.1038/s44318-025-00455-8 (PMC12218407; doi:10.1038/s44318-025-00455-8)

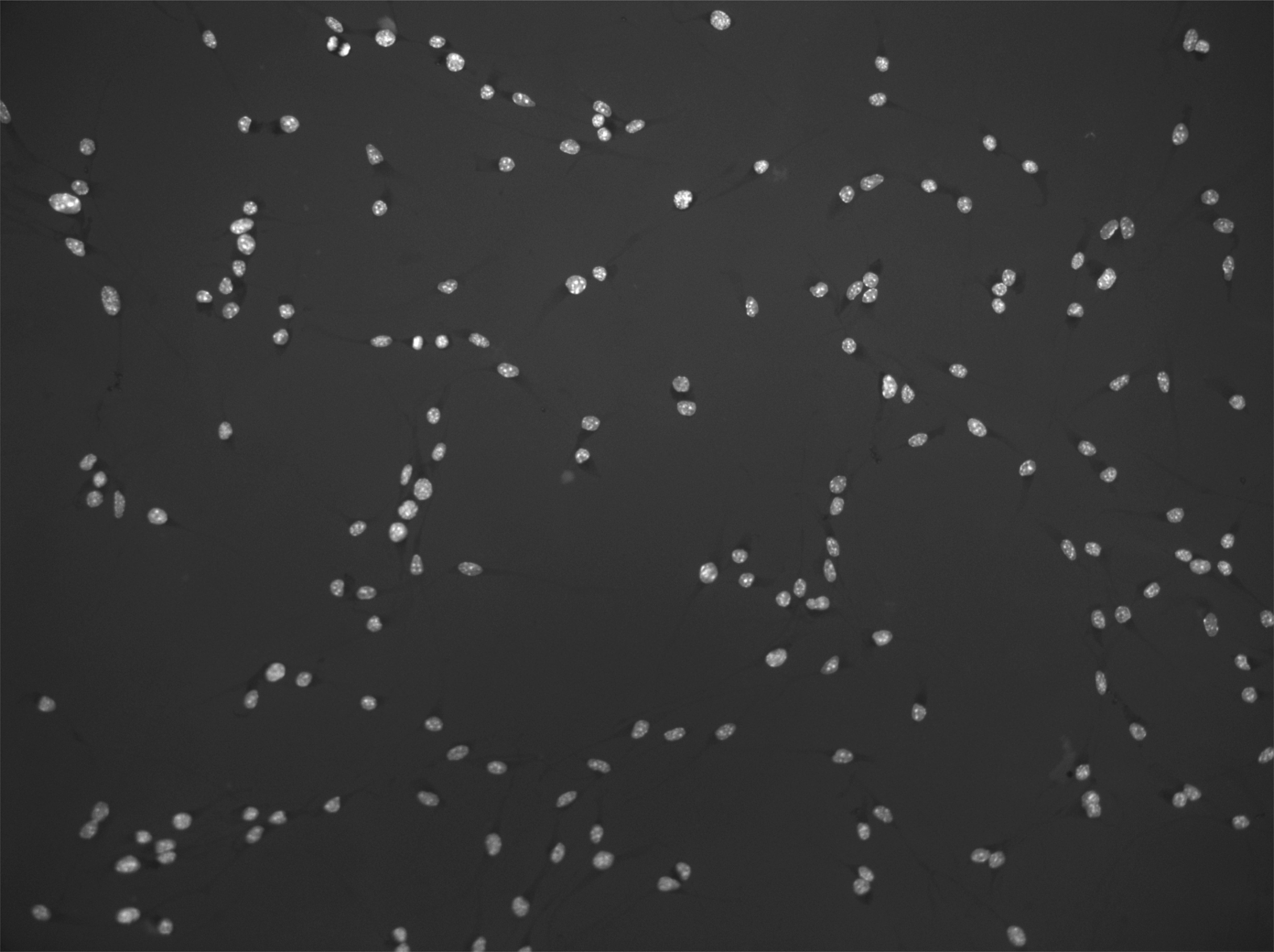

Supplement: Supplementary file 14 — Appendix Figure Source Data sd_S9 [file 44318_2025_455_MOESM14_ESM.zip › S9/E/S8iDMSO/S9_E_S8iDMSO_1.tiff]

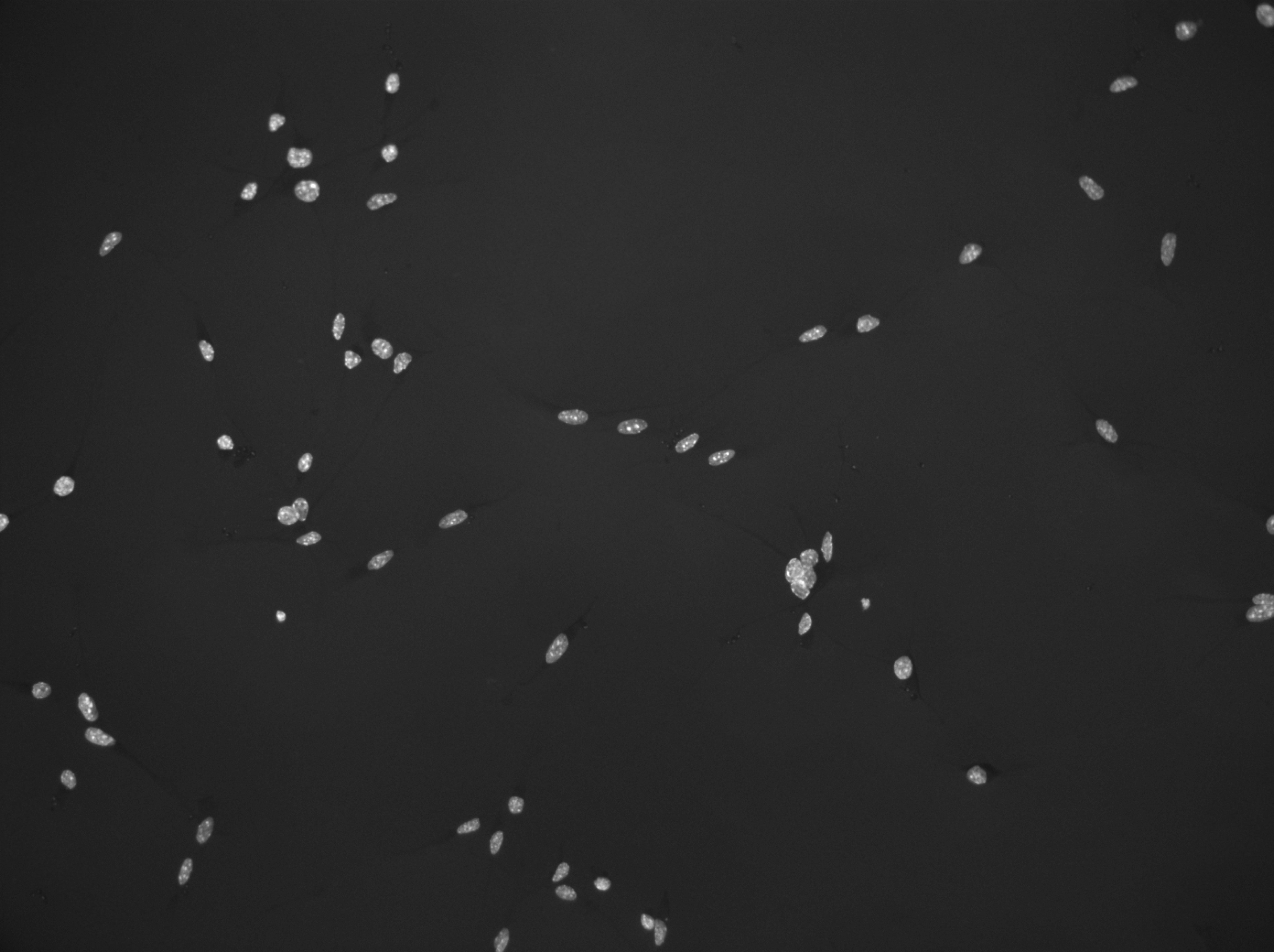

Supplement: Supplementary file 14 — Appendix Figure Source Data sd_S9 [file 44318_2025_455_MOESM14_ESM.zip › S9/E/S8i/S9_E_S8i_1.tiff]

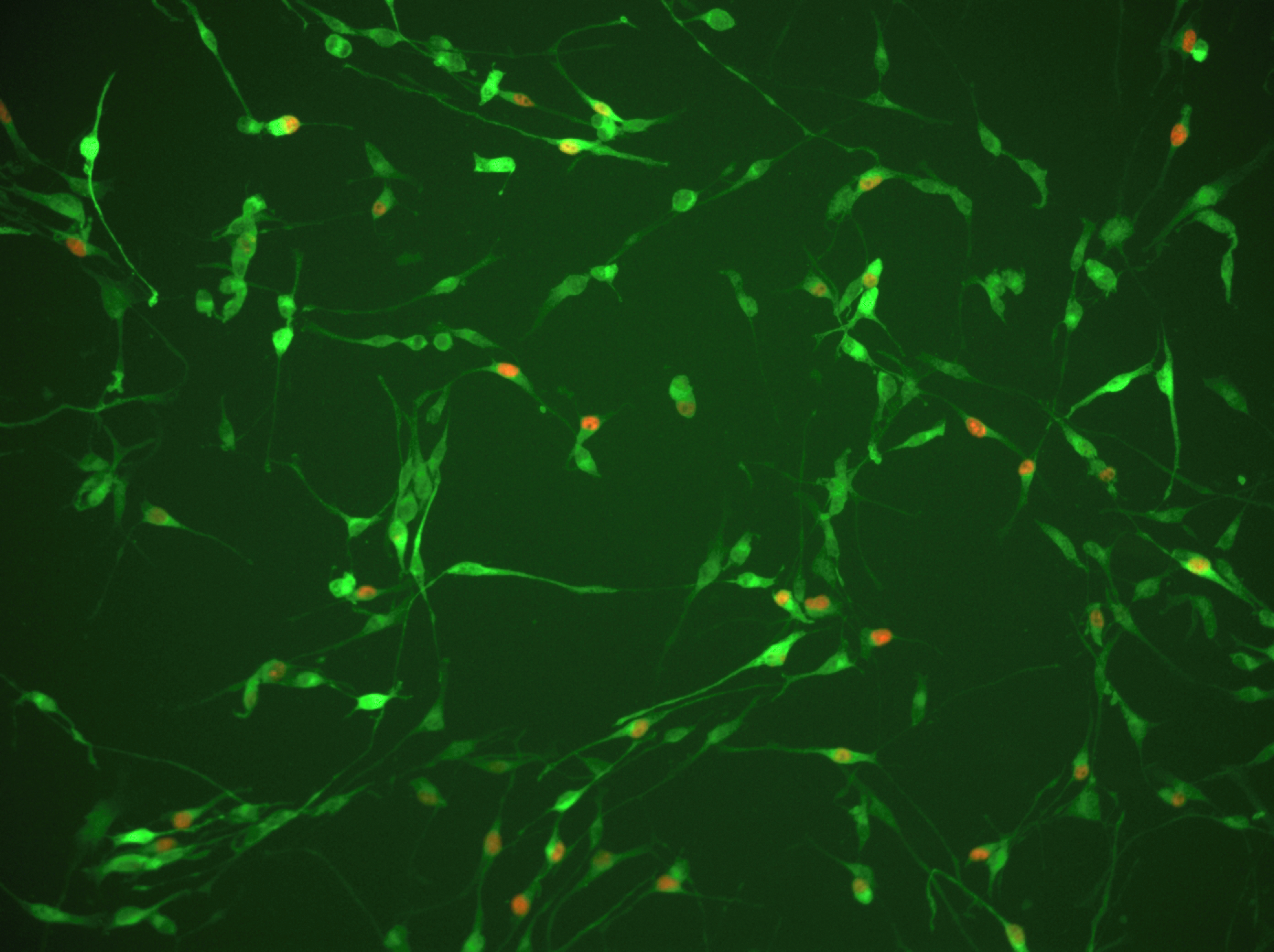

Supplement: Supplementary file 14 — Appendix Figure Source Data sd_S9 [file 44318_2025_455_MOESM14_ESM.zip › S9/E/S8iDMSO/S9_E_S8iDMSO_3.tiff]

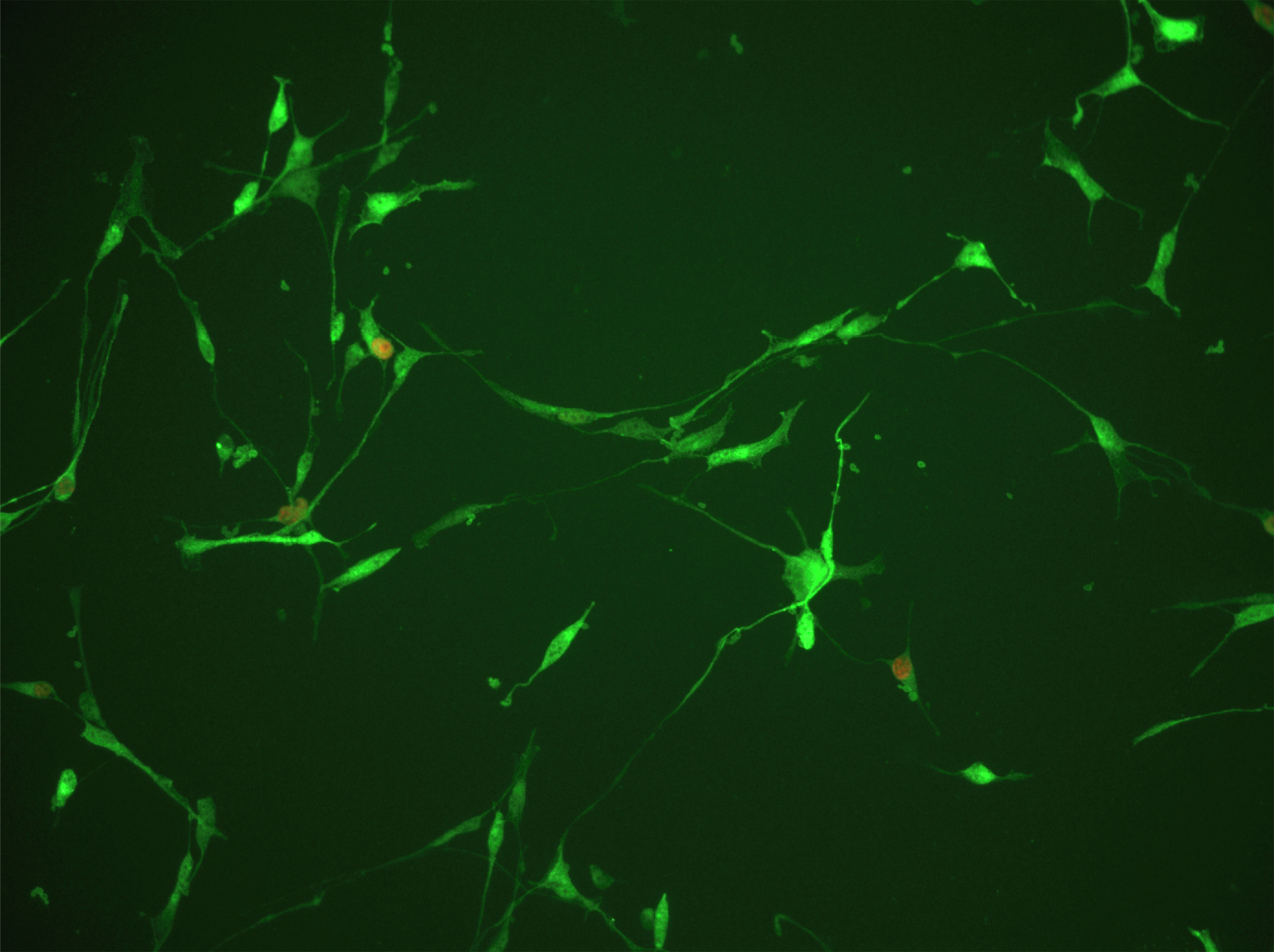

Supplement: Supplementary file 14 — Appendix Figure Source Data sd_S9 [file 44318_2025_455_MOESM14_ESM.zip › S9/E/S8i/S9_E_S8i_3.tiff]

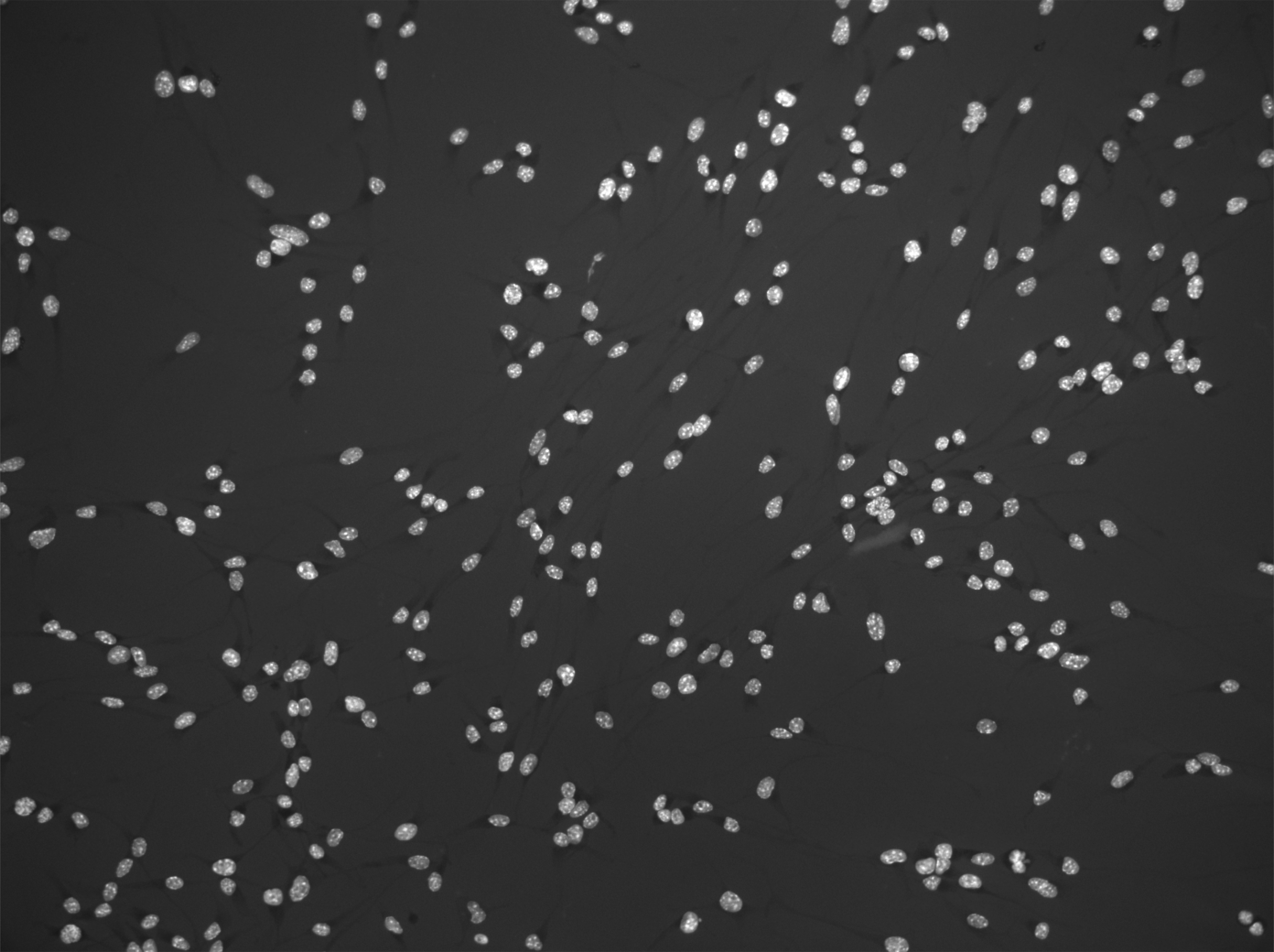

Supplement: Supplementary file 14 — Appendix Figure Source Data sd_S9 [file 44318_2025_455_MOESM14_ESM.zip › S9/E/Ctrl/S9_E_DMSO_1.tiff]

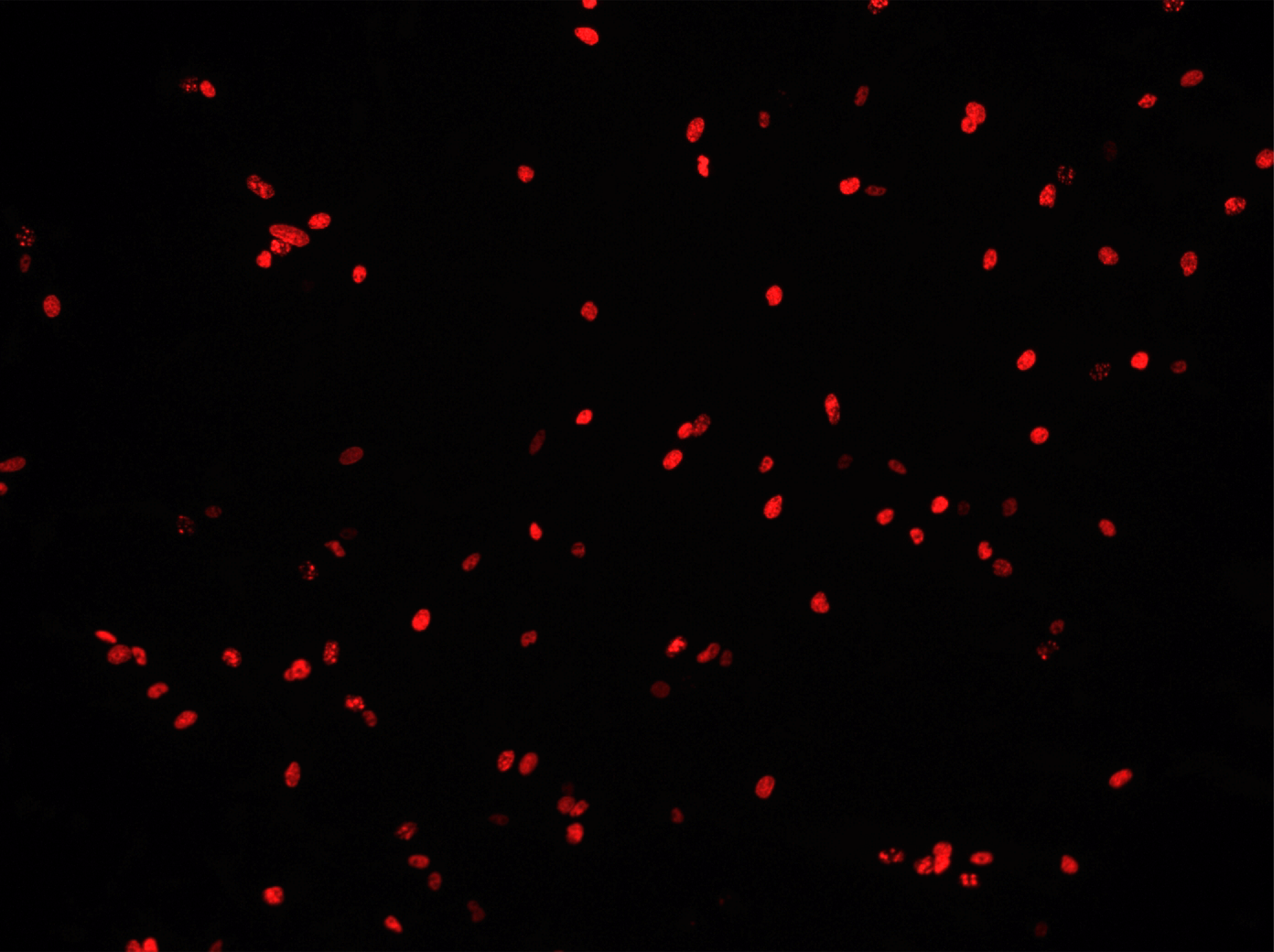

Supplement: Supplementary file 14 — Appendix Figure Source Data sd_S9 [file 44318_2025_455_MOESM14_ESM.zip › S9/E/Ctrl/S9_E_DMSO_2.tiff]

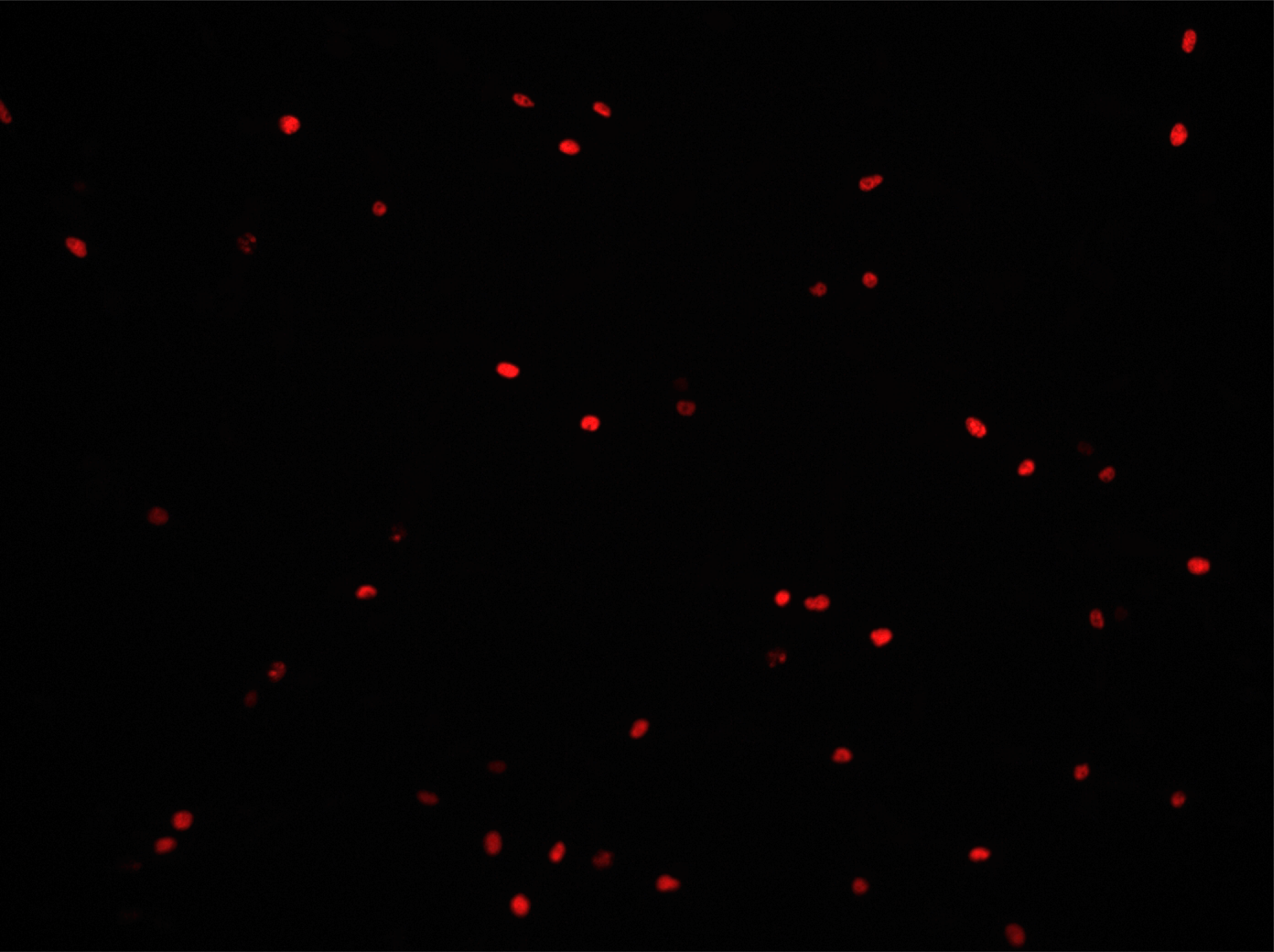

Supplement: Supplementary file 14 — Appendix Figure Source Data sd_S9 [file 44318_2025_455_MOESM14_ESM.zip › S9/E/S8iDMSO/S9_E_S8iDMSO_2.tiff]
